# Population-enriched innate immune variants may identify candidate gene targets at the intersection of cancer and cardio-metabolic disease

**DOI:** 10.3389/fendo.2023.1286979

**Published:** 2024-03-21

**Authors:** Susan Yeyeodu, Donia Hanafi, Kenisha Webb, Nikia A. Laurie, K. Sean Kimbro

**Affiliations:** ^1^ Julius L Chambers Biomedical/Biotechnology Institute (JLC-BBRI), North Carolina Central University, Durham, NC, United States; ^2^ Charles River Discovery Services, Morrisville, NC, United States; ^3^ Department of Microbiology, Biochemistry, and Immunology, Morehouse School of Medicine, Atlanta, GA, United States

**Keywords:** innate immune variants, pleiotropic actions, cancer disparities, cardio-metabolic disparities, population-enriched variants, candidate protein targets

## Abstract

Both cancer and cardio-metabolic disease disparities exist among specific populations in the US. For example, African Americans experience the highest rates of breast and prostate cancer mortality and the highest incidence of obesity. Native and Hispanic Americans experience the highest rates of liver cancer mortality. At the same time, Pacific Islanders have the highest death rate attributed to type 2 diabetes (T2D), and Asian Americans experience the highest incidence of non-alcoholic fatty liver disease (NAFLD) and cancers induced by infectious agents. Notably, the pathologic progression of both cancer and cardio-metabolic diseases involves innate immunity and mechanisms of inflammation. Innate immunity in individuals is established through genetic inheritance and external stimuli to respond to environmental threats and stresses such as pathogen exposure. Further, individual genomes contain characteristic genetic markers associated with one or more geographic ancestries (ethnic groups), including protective innate immune genetic programming optimized for survival in their corresponding ancestral environment(s). This perspective explores evidence related to our working hypothesis that genetic variations in innate immune genes, particularly those that are commonly found but unevenly distributed between populations, are associated with disparities between populations in both cancer and cardio-metabolic diseases. Identifying conventional and unconventional innate immune genes that fit this profile may provide critical insights into the underlying mechanisms that connect these two families of complex diseases and offer novel targets for precision-based treatment of cancer and/or cardio-metabolic disease.

## 1 Introduction

### 1.1 Double-edged swords: important factors connecting metabolic disorders and cancer development

The following perspective was written in response to an invited *Frontiers* research topic to explore methods, mechanisms, and hypotheses that may ultimately identify and exploit biological processes contributing to complex disease progression and molecular interactions enabling cross-talk between cancer and cardio-metabolic disease. Based on our hypothesis that innate immunity differences contribute to observed population disease disparities in cancer and metabolic disorders, we apply a functional genomics approach to identify specific innate immune genes as potential therapeutic targets at the intersection of these two complex disease families.

### 1.2 Framing precision drug target discovery in the context of health disparities

#### 1.2.1 Defining health disparities

The US National Institute on Minority Health and Health Disparities (NIMHD) defines health disparities as “a health difference (compared with the general population), based on one or more health outcomes (such as the overall rate of disease incidence, prevalence, morbidity, mortality or survival) that adversely affect disadvantaged populations.” In the US, such populations include Blacks/African Americans, Hispanics/Latinos, Asians, American Indians/Alaska Natives, and Native Hawaiians/other Pacific Islanders) (1). Diverse sources, from sponsored websites (such as 2 and associated links) to peer-reviewed articles summarizing disparities in one or more diseases between two or more populations, provide ample evidence for differences in cancer (3), cardio-metabolic disease (4) and overall health risks and outcomes (5) based on ethnic background/geographic ancestry. By way of illustration, **Tables 1**, **2** summarize disparities in cancer incidence and mortality among US ethnic populations (adapted from 6) and population differences in overall mortality rates of cancer and cardio-metabolic diseases (adapted from 7), respectively.

**Table 1:** Ethnic Disparities in US Cancer Incidence and Mortality.

	EA	AA	ASN/PI	NA/AN	HISP
**Cancer Incidence**	breast	prostate	*stomach*	colon kidney liver lung stomach uterine	*uterine*
**Cancer Mortality**	lung	breast colon prostate uterine	*stomach*	kidney liver stomach	*liver*

adapted from "Table 9. Incidence and Mortality Rates for Selected Cancer by Race and Ethnicity, US" (6).

standard font indicates most frequently occuring cancer among aggregate populations; italics indicate most frequently occuring cancer for a specific ethnic group (not aggregate).

EA, European American, non-Hispanic White; AA, African American, non-Hispanic Black; ASN/PI, Asian American/Pacific Islander; NA/AN, Native American/Alaskan Native; HISP, Hispanic/Latino.

**Table 2:** Deaths from Cancer, Cardio-Metabolic, and Infectious Diseases in the US as of 2018.

Cause of Death	Aggregate	EA	AA	NA/AN	ASN	PI	HISP
**heart disease**	23.1%	23.4%	**23.6%**	*18.0%*	21.3%	23.5%	19.8%
**cancer**	21.1%	21.2%	20.4%	*16.8%*	**25.1%**	21.9%	20.5%
**stroke**	5.2%	5.1%	5.7%	*3.6%*	**7.6%**	6.2%	5.5%
**diabetes**	3.0%	*2.5%*	4.5%	5.6%	4.1%	**7.3%**	4.6%
**infection (flu, pneumonia)**	2.1%	2.1%	*1.8%*	2.3%	**3.3%**	2.2%	2.1%
**kidney disease**	1.8%	*1.6%*	**2.8%**	1.8%	2.1%	2.2%	2.1%
**liver disease**	*	1.4%	1.0%	**6.2%**	*0.9%*	1.3%	3.2%
**hypertension**	*	*1.1%*	1.9%	*1.1%*	**2.1%**	1.4%	1.4%

adapted from Tables C & D in "Deaths: Leading Causes for 2018." Heron, M. National Vital Statistics Reports 70(4) (7).

bold indicates highest mortality rate for given cause of death.

italics indicates lowest mortality rate for given cause of death.

*aggregate data were only available for top ten causes of death.

Assessing health differences between populations is complicated because results may vary depending on the size and granular composition of the populations being compared. On the one hand, evaluating larger, more heterogeneous populations improves statistical reliability, but this approach may mask disparities among subpopulations. For example, among Asians in the US (8) and Asia (9), the incidence of liver cancer varies widely based on geography and/or geographic ancestry. Further, trends in incidence and/or mortality may change due to cohort variations in age, exposure to risk, and geographic location, as is the case for liver (10) and breast cancer incidence (11) in the US and for global cancer mortality rates (e.g., 12).

Defining/distinguishing populations is a critical aspect of evaluating health disparities. Many analyses have been based on self-identified ethnicity; it stands to reason that this approach is likely to align more closely with social determinants of health. In contrast, a relatively precise biological assessment of geographic ancestry can be obtained using genetic markers to identify ethnic origins. In this approach, selected ancestry informative markers (AIMs) were initially used to evaluate genetic admixture and geographic ancestry and provide valuable background information when comparing individuals representing different populations (13). Improved methods and more extensive and complete reference datasets have further refined admixture mapping (14).

For the purposes of this perspective, we will refer to populations as they are defined by individual authors; populations in Section 3 are defined according to Karczewski (15). The interested reader is referred to a recent book chapter entitled “Using Population Descriptors in Genetics and Genomics Research: A New Framework for an Evolving Field” written by the National Academies of Sciences’ Committee on the Use of Race, Ethnicity, and Ancestry as Population Descriptors in Genomics Research (16) for a thorough treatment of this subject.

#### 1.2.2 Past and present challenges to advancing research in the biology of health disparities

Cancer and cardio-metabolic disease disparities have multifactorial etiologies, including biological, behavioral, environmental, and social components. There is ample evidence that these disparate etiological factors are not adequately understood in isolation from one another. The interested reader is referred to reviews on the impact of physical, social, and chemical environments on the biology of health disparities (17–19) and on the biological impacts of stress (20), including racism-induced stress and increased allostatic load (21–23), all of which are beyond the scope of this perspective.

The relative contribution of biology to cancer and cardio-metabolic disparities continues to be a matter of debate among scientists in various disciplines and even among biologists themselves (24). The hesitation to consider geographic ancestral differences in biology among some mainstream biomedical scientists is just one of several obstacles that have hindered a rigorous study of the biology of health disparities.

Social forces continue to hinder the participation of minority populations in medical research and to limit their access to medical care. For example, an entrenched and well-founded mistrust of the medical establishment in the US exists among minority populations due to a long history of abuses (25). Limited access to healthcare and subpar healthcare quality further exacerbate health disparities in minority populations, leading to lower life expectancy in American Hispanic and Black populations (26).

Traditional research approaches and the most widely available resources in the biomedical sciences have also unintentionally hindered a rigorous characterization of the biological differences that underlie health disparities. *In vitro* studies employ samples and cell lines obtained most often from individuals of European descent (27, 28) and the majority of clinical trials disproportionately enroll individuals from this same population (29, 30). Thus, at multiple stages in the drug research and development cycle, biases exist towards agents optimized for those of European ancestry. Fortunately, the need to increase the diversity of human samples and cell lines and to engage diverse study populations in biomedical research and clinical trials has recently gained the attention and enthusiastic support of pre-eminent scientists (29, 31–33) and the NIH (34).

#### 1.2.3 Considering geographic ancestry in the development of effective treatments

The human genome possesses a high degree of variation. According to a 2016 meta-analysis of 60,706 individuals of diverse ancestries, an average of 1 in 8 bases of the coding sequence were variants, and 72% of these had not been previously identified and/or characterized (35). Wide genetic variations within populations are at least as diverse as genetic variations between populations (36). This finding implies that not all genetic variations contribute to putative biological differences between populations.

Genetic differences associated with geographic ancestry, such as AIMs, may result in the uneven among populations distribution of gene variants. In many cases, these variants are uncommon, and/or their impact on protein expression, function, or disease is either insignificant or unknown. However, an intriguing study by Ahsan et al. (37) identified 65 “minor” drug response alleles that were present in more than 50% of individuals in at least one population; in other words, in some populations, the variant was more common than the wild type/canonical protein. Consistent with this is a body of clinical evidence that specific drug responses vary according to geographic ancestry, with outcomes that range from lack of efficacy to drug-related pathology and death in one or more minority populations (38–40). Therefore, we sought to identify population-specific potential therapeutic targets at the intersection of cancer and cardio-metabolic disease, in part by hand-curating gene variants with “minor” alleles that were common in at least one major population (as defined by 15) but that were significantly less common in at least one other major population.

### 1.3 Innate immunity as a biological driver of health disparities

Gene variants that confer protective immunity are retained in each population to optimize survival. For example, in the case of those with African ancestry, gene variants retained in the pan-African genome have been identified that provide defense against indigenous pathogens such as malaria and trypanosomiasis (African sleeping sickness/Chagas disease). The selective pressure imposed by pathogens on gene variation is impressive; in the case of malaria, variants of at least 40 different genes are thought to protect against one or more species of *Plasmodium* (41, 42).

Unfortunately, immune protection frequently involves a trade-off where protective innate immune variants may introduce new pathologies. For example, among the gene variants that protect against malaria, *HbS* also promotes sickle cell anemia, *HbE* promotes thalassemia, *G6PD* variants promote hemolytic anemia, and Duffy antigen receptor (*DARC*) variants are associated with increased breast cancer metastasis and mortality (43, 44). Similarly, the same *APOL1* variants shown to protect against severe trypanosomiasis are also associated with nephropathy (45, 46).

Several lines of evidence affirm that innate immune genes are highly adaptable and optimized to respond to local pathogens. First, within the human genome, genes associated with immunity are under the strongest selective pressure (47, 48). Second, selective pressure on immune genes is pathogen-driven (49, 50). Third, the geographic distribution of populations bearing the highest frequency of *HbS* (51) and *DARC* (52) gene variants closely resemble the geographic distribution of the malarial strains they protect against. Finally, according to their geographical ancestry, populations differ in their susceptibility to infectious disease (53), in their immune response to pathogens (54) and even in their macrophage function and circulating cytokine levels (55–57). All of these findings indicate that protective innate immune variants are distributed among individuals based on their geographic ancestry.

It is important to note that genes associated with innate immunity are structurally and functionally diverse. Some are well-characterized participants in inflammation, including but not limited to cytokines, chemokines, and pattern recognition receptors (lectins, Toll-like receptor (TLR) family members, and NLRs) and their related pathways. However, as illustrated by the variety of genes that protect against malaria (summarized in **Table 3**), others are pleiotropic, expressed in non-immune tissues and/or frequently better known for their “day jobs”. Most of the protective variants listed in **Table 3** can be tied directly to immunity. Still, a few (such as *APOE*, *G6PD*, glycophorin (*GYP*), hemoglobin (*HB*), and haptoglobulin (*HP*)) would be considered unconventional innate immune genes.

**Table 3:** Innate immune genes that provide protection against malaria (adapted from 41, 42).

Gene	Name/Function	Expression	Association with Disease (based on titles available in Google Scholar)
*ABO*	ABO blood group	secreted	cancer, cardiovascular disease, diabetes, obesity, NAFLD
*APOE*	apolipoprotein E	secreted	cardiovascular disease, obesity, diabetes, NAFLD, cancer
*CD36*, thrombospondin receptor, scavenger receptor B3	broad specificity receptor for proteins and lipids	adipose, liver, others	cardiometabolic disease, cancer
*CR1, CD35*, C3b/C4b complement receptor, Knops blood group antigen		erythrocytes, leukocytes, glomerular podocytes, splenic DCs	gallbladder and liver cancer, diabetes, kidney disease
*DARC, FY, ACKR1, CD234, CCBP*1	Duffy atypical chemokine receptor	erythrocytes, endothelia	breast cancer, prostate cancer, cardiometabolic disease
*FCGRA2, CD32*	low affinity Fc receptor	phagocytes	breast cancer, cardiovascular events
*G6PD*	glucose-6-phosphatase dehydrogenase rate-limiting step to pentose-phosphate, NADPH	lymphoblasts, granulocytes	cancer, diabetes, cardiovascular disease
*GYPA,B,C, CD235a,b,c*	glycophorin A,B,C sialoglycoproteins	A broad expression	
		B,C erythrocytes	C leukemia, oral cancer
*HBA, HBB*	hemoglobin, O2/CO2 transport	erythrocytes	thalassemia, sickle cell anemia
*HLA-B*	component of MHC class I	broad expression	elimination of infected or transformed cells
*HLA-DR series (A,B1,3,4,5)*	components of MHC class II	antigen presenting cells	elimination of infected or transformed cells
*HP*	haptoglobulin, plasma protein that binds Hb	liver, others	diabetic nephropathy and coronary artery disease
*ICAM1, CD54*	intercellular adhesion molecule 1, receptor for CD11a or b/CD18 integrins and rhinovrius	immune and endothelial cells	cancer, diabetes, obesity
*IFNAR1,2*	interferon alpha (and beta) receptor, subunits 1 and 2	broad expression	1 gastric, colorectal, breast cancer; 2 lung cancer, diabetes
*IFNG*	interferon gamma	circulating	cancer, diabetes
*IFNGR1,2*	IFN gamma receptor 1 (CD119), 2	broad expression	1,2 cancer
*IL1A/IL1B*	interleukin 1A, 1B	circulating	A, B cancer, obesity; B diabetes
*IL1RN*	IL1 receptor antagonist	secreted	cardiovascular disease, cancer, obesity, diabetes
*IL10*	interleukin 10	circulating	cancer, obesity, diabetes, atherosclerosis
*IL10RB*	IL10 receptor, subunit beta	broad expression	obesity
*IL12B*	interleukin 12, beta subunit	circulating	diabetes, cancer
*IL4*	interleukin 4	circulating	cancer, diabetes
*IRF1*	interferon regulatory factor 1	broad expression	cancer
*MBL2*	mannose binding lectin 2, collectin 1	circulating	cancer, diabetes, atherosclerosis
*MST1, HGFL*	macrophage stimulating 1, hepatocyte growth factor like	secreted	cancer, diabetes, NAFLD, cardiovascular disease
*NCR3, CD337*	natural cytotoxicity triggering receptor 3	NK cells	cancer
*NOS2A*	nitric oxide synthase 2	liver, retina, bone, lung, cartilage, fat	cancer, diabetes
*PECAM1, CD31*	platelet-endothelial cell-adhesion molelcule 1	immune and endothelial cells	cancer, cardiovascular disease, diabetes
*PSMB9*	proteosome 20S subunit beta 9	MHC II expressing tissues	cancer, diabetes
*SCL4A1, CD233*, erythrocyte band 3 prot.	chloride/bicarbonate exchanger, Diego blood group	erythrocytes, kidney, bone, others	
	CO2 transport from tissues to lungs, structural protein		cardiovascular disease, colorectal cancer
*SELE, CD62E*	selectin E	endothelia	cancer
*TCRB*	T-cell receptor, beta subunit	T-cells	diabetes, cancer
*TIRAP, MAL*, Myd88-2	TIR domain containing adapter protein	broad expression	cancer, diabetes, NAFLD
*TL4*	Toll-like receptor 4	broad expression	cancer, obesity, diabetes, NAFLD, cardiovascular
*TAP1, ABCB2*	transporter 1, ATP binding cassette, subfamily B	broad expression	cancer, diabetes
*TNF*	tumor necrosis factor	circulating	cancer, obesity, diabetes, NAFLD, cardiovascular disease
*TNFSF5*	CD40 ligand, CD154, TNF superfamily member 5	circulating	diabetes, cancer, cardiovascular disease, obseity

### 1.4 Inflammation is a component of cancer and cardio-metabolic diseases

#### 1.4.1 Cancer and inflammation

Hanahan and Weinberg, in their seminal review, describe six hallmarks of cancer, many of which are enabled by mechanisms of immunity, including inflammation (58). Their observations are particularly relevant to this perspective since further research in the field has established that reprogrammed energy metabolism and immune evasion are additional hallmarks (58, 59).

In a previously published perspective, we presented evidence for an association between breast and prostate cancer disparities in African Americans (AAs) and classic innate immune gene variants (interleukins, Toll-like receptors, monocyte activity) more commonly found in AAs (60). Since 2019, Google Scholar (accessed 4/18/23) has listed more than 18,000 publications with titles that include “cancer” and “inflammation,” “infection”, “immune,” “immunity,” or “innate”; these publications address a wide range of topics, including immune escape by cancer cells, the contribution of chronic inflammation to tumor progression, and immune-based cancer therapies, that are beyond the scope of this perspective. Notably, less than 40 of these publications (< 0.2%) include the terms “disparity” or “disparities” in their titles. Among this small set of publications are descriptions of population differences in tumor microenvironment and immune signatures in breast (61, 62), head and neck (63–65), lung (66, 67), and colorectal (68, 69) cancers, as well as cancer generally (70). Of particular interest is a recent exploration of the link between racial differences in mitochondrial metabolism and the tumor immune microenvironment (71).

#### 1.4.2 Cardio-metabolic disease and inflammation

The constellation of inter-related cardio-metabolic diseases has been collectively referred to as metabolic syndrome (MetS), and their cumulative effect on global health is massive (reviewed in 72–74). Clinical definitions of MetS vary depending on which disease(s) are of primary interest (reviewed in 75–77). The National Heart Lung and Blood Institute (NHLBI) lists the following MetS risk factors as abdominal obesity and/or insulin resistance, elevated triglycerides and LDL-cholesterol, reduced HDL-cholesterol, hypertension, elevated glucose and pro-thrombotic or pro-inflammatory states (78). Several metabolic diseases have been associated with these risk factors, including hypertension, obesity, atherosclerotic cardiovascular disease, type 2 diabetes (T2D), non-alcoholic fatty liver disease (NAFLD), and stroke.

Genetic and environmental factors impact cardio-metabolic diseases, and their risk, morbidity, and mortality vary with age, gender, and race/ethnicity (4, 76). Unfortunately, the effects of MetS are not confined to cardio-metabolic co-morbidities, given that MetS is also associated with increases in the incidence and/or mortality of arthritis, chronic kidney disease, schizophrenia, depression and cancer, as noted in references (79, 80).

Inflammation is a key contributor to MetS and associated co-morbidities (81–83), just as MetS pathologies impact inflammation (c.f. 84). In general, low-grade chronic inflammation evoked during metabolic disease stimulates the production of pro-inflammatory cytokines, immuno-modulatory proteins, lipids, and other mediators of inflammation that impact systemic and/or localized tissue inflammation (82, 85). Unfortunately, the treatment of metabolic diseases is complicated by the cross-talk between pro- and anti-inflammatory mechanisms at work among MetS co-morbidities (c.f. 77, 86–88). Further, inflammation from one metabolic disease can also exacerbate other MetS co-morbidities.

As with almost all tissues, organs that regulate systemic metabolism possess innate immune response capabilities. Notably, some organs that regulate overall metabolic homeostasis also impact systemic inflammation. In the case of both adipose tissue (89–91) and liver (92–94), these organs harbor and partner with resident macrophages (ATMs and Kupffer cells, respectively) in inflammation. Further, adipose tissue and liver produce unique immunologically active biomolecules, such as adipokines (86, 95) and bile acids (96–99). Perhaps less appreciated are two additional organs associated with metabolic homeostasis that control systemic levels of immunologically active biomolecules: the gallbladder regulates bile acid levels and the pancreas controls insulin, which levels of insulin, with its known anti-inflammatory effects (100).

Just as mediators of metabolism can impact inflammation, mediators of immunity can impact metabolism. For example, innate immune receptors have demonstrated roles in metabolic disease progression (101), and pro-inflammatory cytokines produced in the adipose tissue of obese individuals contribute to the development of T2D (102). Significantly, biomolecules such as adipokines, insulin, and bile acids mediate metabolism and inflammation. Further, besides their widely recognized role in lipid transport and cellular metabolic homeostasis, serum lipids and lipoproteins also provide innate immune protection (103, 104).

## 2 A functional genomics approach to novel target discovery

Using functional genomics, we and others have observed associations between specific innate immune gene variants and cancer or metabolic disease risk or outcome that differ according to geographic ancestry (57, 60, 105). Given that immunity including inflammation contributes to the progression of both complex disease families, we have hypothesized that population differences in genetic (and epigenetic) innate immune programs contribute to complex disease disparities between populations. Based on this conceptual framework, this perspective seeks to identify innate immune gene candidates associated with both cancer and cardio-metabolic disease that differ between populations.

Genome wide association studies (GWAS) in general (106) and the Genome Aggregation Database (gnomAD) in particular (107) provide researchers with the capacity to compare thousands of complete genomes from individuals among all largely-grouped populations. These resources catalog gene variations called single nucleotide polymorphisms (SNPs) across the entire genome of each individual. SNPs are located not only in protein coding genes (including coding exons as well as non-coding introns and remote, up-, down-, and mid-stream regulatory sites), but also across regions associated with short and long non-coding RNAs, chromosomal architecture, and other essential functions that have been previously underappreciated and mislabeled as “junk DNA” (108). The number of genes and the percentage of the human genome they occupy varies depending on their definition (109). Notably, most SNPs associated with disease states or changes in phenotype (95%) are located outside coding exons (110).

Nevertheless, in this perspective, we will focus on widely occurring gene variants that code for changes in the canonical amino acid (aa) sequence, also referred to as missense variants or nonsynonymous SNPs, as a first step towards accelerating the development of optimally safe and active drugs that target understudied protein variants widely found in patients with diverse geographical ancestries. Importantly, nonsynonymous SNPs have the potential to impact protein conformation, activity and/or protein-protein interactions, potentially altering disease states and phenotypes. For simplicity, we have also excluded synonymous SNPs (exonic point mutations that do not alter aa sequence), in spite of mounting evidence that suggests they can function in isoform selection (protein size and sequence), transcript expression levels and stability, translational folding rate, overall conformation, and posttranslational modifications, all of which possess potential functional consequences on cell behavior and disease risk (111–113).

This perspective identifies conventional and unconventional innate immune genes (summarized in Section 3) that meet the following criteria. First, there is evidence that each gene participates in, is a target of, or is associated with innate immunity including inflammation. Second, there is evidence that each gene is associated with at least one form of cancer and at least one cardio-metabolic disease. Finally, each gene occurs among the global population as at least one population-enriched variant, which we define as a widely occurring missense variant distributed unevenly among populations.

We have employed a hand-curated discovery process to identify population-specific innate immune genes at the intersection of cancer and metabolic disease. From the primary and secondary literature, gene lists associated with innate immunity (49, 114, 115), cancer (116, 117), or cardio-metabolic disease (118, 119) were vetted for the following characteristics:

1) Evidence in the primary or secondary literature (accessed through Google Scholar) indicated that the candidate gene was involved in all three disease categories: innate immunity/inflammation, cancer, and cardio-metabolic disease.
2) Indication in gnomAD that the candidate gene occurs as at least one nonsynonymous SNP/missense variant with
  - a high minor allele frequency (MAF ≥ 0.2 in at least one of the six major populations defined by 15): African/African American (AFR/AA), East Asian (E ASN), non-Finnish European (EUR), Latino/Latina (LAT), Middle Eastern (MID E), and South Asian (S ASN),
  - a difference in MAF among significant populations of ≥ 0.2 from the highest to lowest frequency.

Note that among genes with missense variants, we chose only those with common variants that occur widely among individuals in one or more populations, i.e., missense variants that occurred in at least 20% of individuals in one or more populations (by definition, having a minor allele frequency (MAF) ≥ 0.20 and varying widely in the frequency of their occurrence among populations. This approach was based on our rationale that variants selected and retained in the human genome provide a survival benefit for the population(s) in which they occur, even as they may also paradoxically contribute to complex disease as discussed above for *HbS* and *APOL1* variants (see Section 1.3).

## 3 Candidate innate immune genes at the intersection of cancer and cardio-metabolic disease disparities

Among the candidate innate immune genes that we identified at the intersection of cancer and cardio-metabolic disease, we found both “conventional” innate immune genes, such as cytokines and cytokine receptors, pattern recognition receptors, and other genes that have widely acknowledged roles in immune cell function, and “unconventional genes” with pleiotropic functions that include innate immunity, such apolipoproteins, biomolecule transporters, and transcription regulators. Using the approach described in Section 2, three lists of innate immune genes implicated in cancer and cardio-metabolic disease were generated. Each gene listed in the three tables below possesses at least one population-enriched variant with an amino acid replacement that differs in its distribution among populations, suggesting its potential role in both cancer and cardio-metabolic disparities. The 52 genes identified provide a representative but not exhaustive list of candidate genes, thus serving as preliminary data for further investigation.

Section 3.1 summarizes conventional innate immune genes and their corresponding population-enriched variants previously shown to impact disease or biological function. Similarly, Section 3.2 summarizes unconventional innate immune genes (better known for their non-immune functions) and their corresponding population-enriched variants that have been previously shown to impact disease or biological function. Finally, Section 3.3 summarizes genes associated with innate immunity, cancer, and cardio-metabolic diseases and their corresponding population-enriched variants whose impact on disease or biological function has not yet been established.

### 3.1 Conventional innate immune genes with previously characterized population-enriched variants

**Table 4** includes 14 genes best known for their roles in immunity, including inflammation, that are present as at least one population-enriched variant shown to impact biological function. Among these are cytokines and cytokine receptors, including macrophage inhibitory cytokine 1 (*MIC-1/GDF15*), interleukin 3 and the alpha subunit of its receptor (*IL3* and *IL3RA*), along with subunits for interleukin 4, 6 and 7 receptors (*IL4R, IL6R*, and *IL7R*), and the leptin adipokine receptor (*LEPR*). Additional immune receptors include the soluble receptor for MHC I antigens I (leukocyte Ig-like receptor A3, *LILRA3/CD85E*) and two pattern recognition receptors, the intracellular pattern recognition receptor nucleotide-binding oligomerization domain containing 2 (*NOD2*) and the five transmembrane stimulator of interferon response CGAMP interactor 1 (*STING1/TMEM173*). Also included were the catalytic enzyme in the rate-limiting step of the kynurenine pathway during inflammation indoleamine 2,3-dioxygenase 2 (*IDO2*), the temperature-sensitive cation channel *TRPM8*, and two adhesion molecules, one expressed in lymphocytes (integrin alpha L, *ITGAL/LFA-1/CD11A*) and the other expressed in leukocytes (junctional adhesion molecule-like, *JAML/AMICA*).

**Table 4:** Candidate Conventional Innate Immune Genes at the Intersection of Cancer and Cardio-Metabolic Disease.

Gene (SNP)	Affected Transcripts	Name/Function	Population MAF Range	Associated w/ Immunity	Ref	Associated w/ Cancer	Ref	Associated w/ Metabolic Disease	Ref
***GDF15***		growth differentiation factor 15, macrophage inhibitory cytokine 1		induced by HCV	120	regulates hepatocellular carcinoma genes	120	stress, metabolic and cardiovascular disease	121
		MIC-1		mediates tissue tolerance	122	pro- and anti-tumor activity	121	deficiency protects against atherosclerosis	123
19-18386331-T-A (rs1059369)	2 (2)	Ser48Thr	0.38 E ASN to 0.14 MID E	systemic lupus erythematosus (SLE) risk in Chinese population	124				
***IDO2***		indoleamine 2,3-dioxygenase 2		immunomodulator	125	multiple cancers	126	NAFLD	118
8-39982715-A-G (rs4736794)	3 (2)	Ile140Val (2 of 4 transcripts)	E ASN 0.34 to 0.03 AFR/AA	major depressive disorder symptoms	127				
8-40005362-C-T (rs10109853)	3 (2)	Arg248Trp (2 of 4 transcripts)	S ASN 0.54 to 0.25 E ASN			multiple myeloma risk in a small Japanese cohort	128		
***IL3***		interleukin 3		hematopoietic growth factor, mast-cell growth factor, multipotential colony stimulating factor	129 130 131	colon cancer risk	132	T2D in obese AA women	133
5-132060785-C-T (rs40401)	1 (1)	Pro27Ser	AFR/AA 0.53 to 0.22 EUR	protection against malaria	134				
***IL3RA***		interleukin 3 receptor, CD123		production and differentiation of hematopoietic progenitor cells	135	leukemia	136, 137	ligand IL3 implicated in T2D in obese AA women	133
X-1378751-G-C (rs17883366)	2 (2)	Val323Leu	MID E 0.26 to 0.06 AFR/AA			colorectal cancer treatment response	138		
***IL4R***		interleukin 4 receptor, CD124, IL4RA, IL13 receptor		ligand IL4 provides protection against malaria, schistosomiasis and helminths	139–141	IL4R overexpressed on the surface of multiple cancer types (breast, lung, etc.)	reviewed in 142	IL-4 dysregulation caused decreased lipid metabolism, decreased lipolysis and increased adipogenesis leading to diseases such as obesity and Type 2 Diabetes	143
16-27362551-A-C (rs1805011)	5 (3)	Glu400Ala	0.53 AFR/AA to 0.06 S ASN	allergy, asthma	144, 145	lung cancer response to radiation	146	type I diabetes?	yes: 147 no: 148, 149
***IL6R***		IL6 receptor, CD126, Gp80		receptor for pleiotropic cytokine IL6	150	lung and other cancers	151, 152	cardiometabolic disease	153
1-154454494-A-C (rs2228145)	3 (2)	Asp358Ala	0.49 LAT to 0.14 AFR/AA			liver cancer	154		
***IL7R***		interleukin 7 receptor, CD127, IL7RA		variants involved in autoimmunity and infectious disease	155	reduced in breast cancer	156	type I diabetes	157
5-35860966-T-C (rs1494558)	5 (4)	Ile66Thr	0.75 AFR/AA to 0.42 E ASN					post-transplantation diabetes	158
5-35871088-G-A (rs1494555)	3 (3)	Val138Ile	0.87 AFR/AA to 0.48 E ASN			gastic cancer in EUR, increase lung cancer	159, 160	BMI in lymphoma patients	161
5-35876172-A-G (rs3194051)	3 (1)	Ile356Val	0.34 AFR/AA to 0.07 E ASN					severe liver disease	162
***ITGAL***		integrin alpha L, LFA-1, CD11A		lymphocyte function associated antigen		renal cancer, gastric cancer prognostic marker	163, 164	bioinformatic assn w aortic valve calcification in metabolic syndrome	165
16-30506720-G-C (rs2230433)	6 (3)	Arg791Thr	0.66 S ASN to 0.14 E ASN			protection against renal cell carcinoma	166		
						risk of IDC breast carcinoma in Han women	167		
***JAML***		junctional adhesion molecule-like, AMICA		regulates inflammatory cell migration	168, 169	lung cancer	151, 170	diabetic nephropathy	171, 172
11-118198037-T-C (rs2298831)	8 (5)	Ile322Met	0.36 AFR/AA to 0.07 S ASN	steroid interaction with Duchenne muscular dystrophy in a multi-center European cohort (n=301 cases)	173				
***LEPR***		leptin receptor, CD295, OBR		required for lymphopoiesis				regulation of fat metabolism, obesity	174, 175
				leptin (ligand) produced by lymphocytes, NK cells, monocytes	176	susceptibility to HBV induced hepatocellular carcinoma	177	NAFLD	86
1-65570758-A-G (rs1137100)	7 (6)	Lys109Arg	0.81 E ASN to 0.10 MID E			colorectal cancer risk	178	early atherosclerosis	179
1-65592830-A-G (rs1137101)	7 (6)	Gln223Arg	0.88 E ASN to 0.34 MID E					obesity in Pacific Islanders	180
***LILRA3***		leukocyte Ig-like receptor A3, CD85E		soluble receptor for MHC I antigens	181	benign prostatic risk hyperplasia	182	elevated plasma HDL	183
					184	lymphomagenesis risk	185	downregulated in obesity, metabolic syndrome	186
19-54803504-A-C (rs6509862)*	3 (3)	Leu107Arg	0.79 AFR/AA to 0.12 EUR	statin intolerance	187				
***NOD2***		nucleotide binding oligomerization domain containing 2, CARD15, NLRC2, BLAU, IBD1		immune response, inflammation	188	triple negative breast cancer, therapeutic target	114, 189	deficiency promotes diabetes and NAFLD in mice	190, 191
16-50710713-C-T (rs2066842)	7 (4)	Pro241Ser	0.28 MID E to 0.01 E ASN			assn with follicular lymphoma survival	192		
***TMEM173***		STING1, stimulator of interferon genes, MPYS		activates IFN innate immune response genes	193, 194	multiple cancers	193, 195	cardiovascular and metabolic disease	196
5-139477397-C-T (rs7380824)	17 (4)	Arg293Gln	0.41 E ASN to 0.14 EUR	LOF, decreased response to bacterial ligands, poxviruses	197–199				
5-139478340-C-G (rs78233829)	18 (4)	Gly230Ala	0.41 E ASN to 0.14 EUR	altered c-di-GMP lid conformation	198				
5-139481493-C-T (rs11554776)	16 (5)	Arg71His	0.41 E ASN to 0.03 AFR/AA	large effect on loss of function	197, 198				
***TRPM8***		transient receptor potential cation channel		immune response, inflammation, temperature regulation	200, 201	multiple cancers	202, 203	obesity, blood pressure	204, 205
2-233955144-G-A (rs7593557)	4 (2)	Ser419Asn	0.55 AFR/AA to 0.05 EUR	cold-induced hyperresponsiveness in bronchial asthma	206			blood lipid profile, BMI in Russian population	207

Genes listed have been associated with innate immunity/inflammation, cancer, and cardio-metabolic disease and have at least one variant in the human genome that occurs in at least 20% (Minor Allele Frequency (MAF) ≥ 0.2) of one or more populations. Missense variants are described by their location in the GRCh38 reference genome (accessed from gnomAD v3.1.2), rs number (reference SNP cluster ID), and amino acid location numbers and identities of the original and coded replacement. Populations are defined by Karczewski 2020 (15): African/African American (AFR/AA), East Asian (E ASN), non-Finnish European (EUR), Latino/Latina (LAT), Middle Eastern (MID E), and South Asian (S ASN). The number of affected transcripts listed include total transcripts (first number) and transcripts with missense mutations (in parentheses) that contain the gene variant, but do not include transcripts of any overlapping genes.

#### 3.1.1 Interleukin 3 and interleukin 3 receptor alpha chain

IL-3 is a growth factor produced by activated T-cells (129) that regulates the growth of hematopoietic progenitor cells and activates mature neutrophils and macrophages (208). IL-3 is also implicated in priming (131) and activating (130) basophils. Intriguingly, increased serum levels of IL-3 have recently been associated with the onset of type 2 diabetes in African American women as determined by serum levels of glucose and HbA1c (133). Genetic variations in *IL3* have been noted in colon and rectal cancers (132). The Pro27Ser variant (5-132060785-C-T) has been associated with protection against malaria (134) but also with an increase in miscarriages following *in vitro* fertilization (IVF) in women of various populations (209).

The interleukin 3 receptor is a heterodimer comprised of an interleukin 3-specific alpha chain (IL-3RA, CD123) and the common cytokine beta chain CSF2RB, another candidate listed below in Section 3.3, that also forms dimers with the alpha chains of both GM-CSF and IL-5 receptors. High-affinity IL-3 binding induces hetero-dimerization of IL-3RA and CSF2RB, and subsequent disulfide linkage of these receptor chains is required for receptor activation and CSF2RB phosphorylation (210). IL-3RA expression varies among CD34+ hematopoietic cell types, with negative/low expression in primitive hematopoietic cells and little or no surface expression in early erythroid progenitors, but high expression in B-lymphoid and myeloid progenitors (135). The X-chromosome-linked *IL3RA* Val323Leu variant (X-1378751-G-C) was associated with non-complete response to neoadjuvant chemotherapy against locally advanced rectal cancer in Hong Kong patients (138).

#### 3.1.2 Interleukin 4 receptor alpha chain

The IL-4R alpha chain (IL4R, CD124) forms heterodimers with at least two partners. Type 1 IL-4 receptors are composed of IL-4R complexed with the common cytokine receptor gamma chain (IL2RG, CD132), which may alternatively dimerize with IL-2, IL-7 and IL-21 cytokine receptors, so that IL-2, IL-7, and IL-21 receptors compete with IL-4R for binding to IL2RG. Type 2 IL-4 receptors are composed of IL-4R complexed with IL-13RA1 (IL13Rα1, CD213A1). Thus, IL-4 activates both Type 1 and Type 2 IL-4 receptors, while IL-13 activates Type 2 IL-4 receptors. Both IL-4 and IL-13 signaling through the IL-4R mediate type 2 (humoral, as opposed to type 1 cellular) immunity against helminths, toxins and tropical parasites such as plasmodium (malaria) and trypanosomes (African sleeping sickness/Chagas disease) (139–141, 211). Both IL-4Rα and IL13-Rα1 have also been implicated in cancer progression and were recently identified as prognostic indicators in soft-tissue sarcoma patients when present in the nucleus. IL-4 regulates lipid metabolism (143), and (142) recent findings highlight an intriguing relationship between non-hematopoietic IL-4Rα activation of a non-canonical signaling pathway that regulates a high-fat, high-carbohydrate diet-driven induction of obesity and impacts the severity of obesity-associated sequelae in mice (212). Numerous genetic epidemiological studies have also shown that *IL4* and *IL4R* and their gene polymorphisms play important roles in asthma in various populations. Notably, individuals carrying one or two copies of the *IL4R* Glu400Ala (16-27362551-A-C) minor allele were at higher risk to suffer from allergy (145) and asthma (144, 213).

#### 3.1.3 Interleukin 7 receptor alpha chain

The integral membrane interleukin 7 receptor (IL-7R) transmits pro-inflammatory signals initiated by IL-7 at the cell surface. The functional IL-7 receptor is a heterodimer comprised of the IL-7 receptor alpha chain (IL7R, IL7Rα, CD127) and the same common cytokine receptor gamma chain (IL2RG, CD132) that dimerizes with the IL-4R alpha chain. The assembled IL-7R recognizes not only IL-7 but also thymic stromal lymphopoietin (TSLP), both cytokines with 4 α-helical strands (214). Multiple transcriptional and post-transcriptional mechanisms exist to regulate expression of the IL-7R protein (215). Some of these mechanisms are homeostatic, molecular and cytokine-mediated, where *IL7Rα* transcription decreases in CD4^+^ and CD8^+^ cells once naïve T cells become activated. Notably, IL-7 binding to IL-7R activates the Janus kinase (JAK/STAT) pathway, which plays an essential role in lipid metabolism (216). However, peripheral blood mononuclear cells (PBMCs) in breast cancer patients show defects in STAT5 phosphorylation and altered expression of IL-7Rα that ultimately impacts memory T cell development (156).

Notably, compared to the canonical gene, the *IL7R* variants 5-35874473-C-T (rs6897932), 5-35860966-T-C (rs1494558) and 5-35871088-G-A (rs1494555) alter the pathology of autoimmune and infectious diseases due to their impact on IL7R expression and alternative splicing (155). Further, all three population-enriched missense variants of *IL7R* identified in **Table 4** show an association with cardio-metabolic disease: Ile66Thr (5-35860966-T-C, rs1494558) with post-transplantation diabetes (158); Val138Ile (5-35871088-G-A, rs1494555) with body mass index (BMI) in lymphoma patients (161), and Ile356Val (5-35876172-A-G, rs3194051) with severe liver disease (162). However, to date only Val138Ile has been associated with increased cancer risk, both in lung (160) and stomach (159).

### 3.2 Unconventional innate immune genes with previously characterized population-enriched variants

**Table 5** includes 18 genes representing several classes of proteins primarily associated with non-immune functions that occur as population-enriched variants shown to impact biological function. These genes include transport membrane proteins, consisting of the multidrug resistance pump (*ABCB1*), the Niemann-Pick cholesterol transporter 1 (*NPC1, SLC65A1*), and the Na+-dependent multivitamin transporter (*SLC5A6*). Among the class of regulatory metabolic enzymes are alcohol dehydrogenase (*ADH1C*), mitochondrial dihydroorotate dehydrogenase (*DHODH*), hydroxysteroid (17-beta) dehydrogenase 4 (*HSD17B4*) involved in peroxisomal fatty acid beta-oxidation, and glycogen phosphorylase B (*PYGB*) involved in regulating glycogen mobilization. Among the genes that participate in signal transduction are the membrane glycoprotein signaling co-receptor neuregulin (*NRG1*), phosphodiesterase 10A (*PDE10A*, which regulates cAMP concentrations), along with the small bioactive neuropeptide neuromedin B (*NMB*). Transcription factors and/or nucleic acid binding protein genes coded as population-enriched variants include hypoxia-inducible factor 2A (*EPAS1, HIF2A*), Iroquois homeobox 2 (*IRX2*), mismatch repair MutL homolog 3 (*MLH3*), the novel intracellular and extracellular ribonuclease T2 (*RNASET2*) and the SURP and G-Patch domain containing 1 (*SUGP1*) splicing factor. Also included are the lipid transport protein apolipoprotein B (*APOB*), the triacylglycerol lipase patatin-like phospholipase domain containing 3 (*PNPLA3*), and the adhesion cadherin family member desmoglein 2 (*DSG2*).

**Table 5:** Candidate Unconventional Innate Immune Genes at the Intersection of Cancer and Cardio-Metabolic Disease.

Gene (SNP)	Affected Transcripts	Name/Function	Population MAF Range	Associated w/ Immunity	Ref	Associated w/ Cancer	Ref	Associated w/ Metabolic Disease	Ref
***ABCB1***		MDR1, P-glycoprotein 1, ATP binding cassette B1		multidrug resistance, xenobiotic protection	217	gallbladder carcinoma, drug resistance	218, 219	Japanese obesity, diabetes and serum lipids in Chinese	220, 221
7-87531302-A-C (rs2032582)	5 (3)	Ser893Ala	0.91 AFR/AA to 0.35 S ASN	drug efficacy	222	increased lung cancer risk	223		
***ADH1C***		alcohol dehydrogenase 1C (class I), gamma		downregulated during inflammation in ulcerative colitis,	224	liver cancer	225, 226	endogenous substrate bile acids involved in lipid, glucose and energy metabolism and impact metabolic syndrome	227, 228
				increased expression reduces IL-6 and IL-8 secretion	224	colorectal cancer	229		
				substrates (estrogen, bile acids) impact innate immunity	96, 230	lung cancer	231, 232		
4-99339632-T-C (rs698)	1 (1)	Ile350Val	0.52 EUR to 0.08 E ASN			increased cancer risk in Africans and Asians	233		
4-99342808-C-T (rs1693482)		Arg272Gln	0.52 EUR to 0.08 E ASN			Japanese upper aerodigestive tract cancer	234		
***APOB***		apolipoprotein B		HCV infection	235	variants associated with NSCLC survival	236	variants in Asian population associated with metabolic syndrome	237
2-21002409-C-T (rs1042034)	1 (1)	Ser4338Asn	0.85 AFR/AA to 0.26 E ASN	HCV infection	235			protective against acute coronary syndrome in Mexican population	238
2-21008652-G-A (rs676210)	1 (1)	Pro2739Leu	0.73 E ASN to 0.15 AFR/AA					stroke	239
2-21028042-G-A (rs679899)	3 (2)	Ala618Val	0.85 E ASN to 0.17 AFR/AA					chronic kidney disease risk among Japanese with hyptertension	240
***DHODH***		dihydroorotate dehydrogenase		defense against bacteria, viruses and protozoa	241–243	multiple cancers, pro-inflammatory ferroptosis	244, 245	glucose metabolism, insulin resistance	246, 247
16-72008783-A-C (rs3213422)	4 (4)	Lys7Gln	0.75 E ASN to 0.34 MID E	rheumatoid arthritis drug response	248–250				
***DSG2***		desmoglein 2		receptor for selected adenovirus serotypes	251	multiple cancers	252	pancreatic islet function, insulin resistance	253, 254
18-31542836-G-A (rs2278792)	1 (1)	Arg773Lys	0.48 E ASN to 0.08 AFR/AA					cardiomyopathy in Yi population	255
***EPAS1***		endothelial PAS domain protein 1, HIF2A, hypoxia-inducible factor 2A		IL31 induction in CD4+ T cells	256	non-small cell lung cancer, colorectal, others	257, 258	dyslipidemia and NAFLD	259
2-46382433-A-C (rs59901247)	2 (1)	Thr766Pro	0.41 AFR/AA to 0.01 S ASN	N-acetylaspartate levels in elite athletes	260				
***HSD17B4***		hydroxysteroid (17-beta) dehydrogenase 4, DBP, MFE-2, MPF-2, SDR8C1		peroxisomal multifunctional protein (detox)	261	overexpressed in prostate cancer	262	peroxisomal fatty acid oxidation	263
						downregulated in non-small cell lung cancer	264	lipid and bile acid metabolism	265
5-119475838-G-A (rs25640)	18 (7)	Arg131His, Arg106Pro or His	LAT 0.56 to AFR/AA 0.17	homozygous D-bifunctional peroxisomal protein disease	266				
5-119525243-T-C (rs11539471)	20 (7)	Trp536Arg	AFR/AA 0.3 to 0.00 E ASN			protective against endometrial cancer	267		
5-119526018-A-G (rs11205)	21 (7)	Ile584Val	LAT 0.53 to 0.29 S ASN			testicular germ cell tumor risk	268		
***IRX2***		Iroquois Homeobox 2		mediates expression of immune regulators MMP9 and VEGF	269, 270	sarcomas, breast, leukemia	271, reviewed in 272	VEGF altered in ischemic stroke and atherosclerosis	reviewed in 273
						nasopharyngeal cancer marker	274		
5-2748943-C-A (rs76906087)	2 (1)	Glu255Asp	0.28 S ASN to 0.03 AFR/AA					present in 5/10 Indian congenital heart defects	275
***MLH3***		mutL homolog protein 3, mismatch repair, HNPCC7		Ig class switch	276	colorectal cancer microsatellite instability	277	mutations only found in breast cancer patients with metabolic disease	278
14-75047125-G-A (rs175080)	4 (3)	Pro844Leu	0.52 MID E to 0.15 E ASN			no assn w CRC in white population	279		
						susceptibility to cervical cancer in Chinese	280		
						hepatocellular carcinoma in Han	281		
***NMB***		neuromedin B		innate immune response to influenza A virus	282	cervical and other cancers	283	highly expressed in adipose, variants related to obesity	284
15-84657289-G-T (rs1051168)	2 (2)	Pro73Ala	0.37 MID E to 0.05 AFR/AA					obesity	284
***NPC1***		Niemann Pick cholesterol transporter, SLC65A1		NKT cell development	285	breast cancer	286	obesity	287, 288
				endosomal entry receptor for ebolavirus	289			type 2 diabetes	290
18-23540480-T-C (rs1805082)	3 (2)	Ile858Val	0.63 E ASN to 0.30 MID E					obesity	291
18-23560468-T-C (rs1805081)	2 (1)	His215Arg	0.41 EUR to 0.08 AFR/AA					cardiovascular disease (Iranian)	292
***NRG1***		neuregulin		macrophage response to yeast	293	NRG1 gene fusions drive multiple solid tumors	294	regulates insulin sensitivity	295
8-32595840-G-A (rs3924999)	21 (17)	Arg30Gln	0.77 E ASN to 0.11 AFR/AA	susceptibility to schizophrenia in Chinese Han	296				
				Fin susceptilibity to reward dependence in major depression	297				
***PDE10A***		phosphodiesterase 10A		mediator of lung and vascular inflammation	298, 299	ovarian cancer target	300	diabetes, diet-induced obesity, insulin sensitivity	301
6-165654841-C-G (rs880121)	10 (2)	Glu15Asp	0.63 MID E to 0.04 E ASN	sporadic Parkinson's in Chinese Han	302				
***PNPLA3***		Patatin Like Phospholipase Domain Containing 3, adipnutrin		platelet and monocyte levels	303	hepatic cancer (Europeans, Han Chinese)	304, 305	NAFLD	118, 303
22-43928847-C-G (rs738409)	4 (2)	Ile148Met (2 of 4 transcripts)	0.42 LAT to 0.14 AFR/AA			hepatic cancer	304, 305	NAFLD	303
***PYGB***		glycogen phosphorylase B		TCR activation stimulates PYGB-dependent glycogenolysis	306	prostate, gastric, non-small cell lung cancers	307–309	carbohydrate metabolism	310
20-25278370-G-T (rs2228976)	1 (1)	Ala303Ser (1 transcript)	0.34 E ASN to 0.08 AFR/AA			present in European desmoid tumors in familiall adenomatous polyposis (FAP)	311		
***RNASET2***		ribonuclease T2, RNASE6PL		degrades microbial RNAs for recognition by TRL8	312, 313	tumor suppressor in lung and ovarian cancers	151, 313	myocardial lipotoxicity in obesity	314
6-166938616-C-A (rs3777722)	14 (1)	Arg226Met	0.4 E ASN to 0.04 AFR/AA	putative association with preterm birth	315				
***SLC5A6***		Na+ dependent multivitamin transporter		anti-inflammatory in murine gut	316	gastric cancer	317	lymphocyte metabolic programming	318
				B lymphocyte maturation	318				
2-27201768-G-A (rs1395)	7 (2)	Ser481Phe	0.86 E ASN to 0.24 AFR/AA					serum levels of glucose (during fasting) and pantothenate	319, 320
***SUGP1***		SURP And G-Patch Domain Containing 1, Splicing Factor 4		altered splicing in innate immunity	321	pan-cancer	322	NAFLD	118
19-19302283-C-T (rs17751061)	8 (1)	Arg290His (1 of 8 transcripts)	0.26 MID E to 0.00 E ASN	serum IgE levels	323			waist-hip ratio fasting insulin and glucose	324 325

Genes listed have been associated with innate immunity/inflammation, cancer, and cardio-metabolic disease and have at least one variant in the human genome that occurs in at least 20% (Minor Allele Frequency (MAF) ≥ 0.2) of one or more populations. Missense variants are described by their location in the GRCh38 reference genome (accessed from gnomAD v3.1.2), rs number (reference SNP cluster ID), and amino acid location numbers and identities of the original and coded replacement. Populations are defined by Karczewski 2020 (15): African/African American (AFR/AA), East Asian (E ASN), non-Finnish European (EUR), Latino/Latina (LAT), Middle Eastern (MID E), and South Asian (S ASN). The number of affected transcripts listed include total transcripts (first number) and transcripts with missense mutations (in parentheses) that contain the gene variant, but do not include transcripts of any overlapping genes.

#### 3.2.1 Multidrug resistance gene

The ATP binding cassette subfamily B member 1 (*ABCB1*) gene is commonly known as the first of two multidrug resistance (*MDR1*) genes in humans and is one of 48 ABC family members (217). ABCB1 functions at the plasma membrane as a 170 kDa monomer with 12 transmembrane domains (TMs), is glycosylated on the first extracellular loop (between TM1 and TM2), and has two intracellular ATP binding sites (one located between TMs 6 and 7, and the other in the carboxy terminus downstream of TM12). ABCB1 is expressed in a wide range of tissues (such as intestine, colon, placenta, liver, and blood-brain barrier) to protect against the intracellular build-up of xenobiotic molecules in vulnerable cells and organs by expelling toxins, including chemotherapeutics, from the cell interior. Thus, ABCB1 has become a widely-known source of and marker for chemoresistance (c.f. 219). ABCB1 also functions as a broad specificity lipid translocase (326). In a Chinese cohort, a variant in the ABCB1 promoter showed pleiotropic effects related to T2D and lipid metabolism (221). Notably, the *ABCB1* Ser893Ala variant (7-87531302-A-C, rs2032582) has been correlated with obesity in a Japanese population (220) and with increased susceptibility to lung cancer in a Spanish cohort (223). This *ABCB1* variant occurs in 91% of Africans/African Americans, but in only 35-62% of other populations (gnomAD) and was shown to impact drug (etanercept) efficacy in the treatment of Chinese Han patients with ankylosing spondylitis (222).

#### 3.2.2 Mismatch repair protein MutL homolog 3

MLH3 is a homolog of the mismatch repair protein MutL. DNA mismatch repair (MMR) proteins play a vital role in maintaining genome integrity and in antibody maturation during class switch DNA recombination and somatic hypermutation (276). In cases of microsatellite instability, tumors often display somatic mutations in MLH3, while hereditary nonpolyposis colorectal cancer type 7 (HNPCC7) has been associated with germline mutations in the same gene (276, 327). Further, reduced MLH3 expression was observed in individuals diagnosed with grade II and III breast cancer, suggesting MLH3 may serve as a reliable susceptibility marker (278, 328). There was no correlation between the *MLH3* Pro844Leu variant (14-75047125-G-A, rs175080, predominantly found in the Middle East) and susceptibility to colorectal cancer in a predominantly white cohort (279). However, in Chinese patients this variant was associated with both cervical cancer (280) and hepatocellular carcinoma (281).

#### 3.2.3 Apolipoprotein B

Lipoproteins enclose otherwise insoluble lipid particles (made up of a central core of cholesterol esters and triglycerides and an outer layer of phospholipids, free cholesterol, and apolipoproteins) for transport through the blood to various tissues (329). Apolipoprotein B (APOB) serves as the primary carrier for several classes of serum lipid particles, including chylomicrons, low-density lipoprotein (LDL), very low-density lipoprotein (VLDL), intermediate-density lipoprotein, and lipoprotein. In LDL particles, APOB interacts with the apoB/E (LDL) receptor, facilitating the removal of LDL cholesterol from the circulation via cellular uptake followed by intracellular LDL breakdown. In a small Japanese study correlating variants of genes related to lipid regulation (including apolipoproteins), the population-enriched missense *APOB* variant 2-21002409-C-T (rs1042034) correlated with HCV infection (235) variant has an allele frequency of 0.85 in African American populations but only 0.26 in East Asian populations (gnomAD). Another population-enriched missense *APOB* variant, 2-21008652-G-A (rs676210) (present in 73% of East Asians vs. 15% of Africans/African Americans (gnomAD)) correlated with the occurrence of initial non-cardioembolic ischemic stroke in a small European cohort (239). A third population-enriched missense *APOB* variant, 2-21028042-G-A (rs679899) (present in 85% of East Asians vs. 17% of Africans/African Americans (gnomAD)) and was protective against acute coronary syndrome in a Mexican population (238). This was associated with both hypertension and chronic kidney disease in a cohort of 3696 Japanese individuals (240).

Functional effects of additional *APOB* missense variants have also been reported. The Arg3638Gln variant (2-21005955-C-T, rs1801701), which is present in no more than 10% of any population, was associated with survival outcomes in non-small cell lung cancer (NSCLC) patients (236). Additionally, two nonsynonymous variants unique to the Asian population, namely 2-21006289-G-A (rs144467873, MAF = 0.001253 and 0.0003594 in East and South Asians, respectively, but < 0.00008 for all other populations (gnomAD v2.1.1) and 2-21029662-G-A (rs13306194, MAF = 0.1343 in East Asians, MAF < 0.007 in all other populations) were evaluated for their association with lipid profiles, metabolic syndrome and risk of diabetes in a large Taiwan Biobank study (237). Both variants were independently associated with total, LDL, and non-HDL cholesterol levels, whereas rs144467873 (Arg3527Trp) was associated with elevated lipid levels and metabolic syndrome, while rs13306194 (Arg532Trp) was linked with serum triglyceride levels.

#### 3.2.4 Dihydroorotate dehydrogenase

Dihydroorotate dehydrogenase (DHODH), which catalyzes the initial and rate-limiting step of the *de novo* pyrimidine pathway, is positioned on the inner mitochondrial membrane (330). DHODH has been a therapeutic target for the treatment of rheumatoid arthritis, psoriasis, autoimmune disorders, and Plasmodium, bacterial and fungal infections (241). For over five decades, elevated DHODH expression has been known to promote tumor progression. *De novo* pyrimidine synthesis becomes essential during increased demands for nucleic acid precursors in rapidly dividing cells making cancer cells highly dependent on DHODH and suggesting that this enzyme is a strategic target for cancer therapy (245). Recently, DHODH was also shown to protect against mitochondrial ferroptosis by preventing the lipid peroxidation that triggers this phenomenon (244). Notably, cancer cells exhibit low levels of glutathione peroxidase 4 (GPX4) and inhibition of DHODH hinders respiration, boosts glycolysis and enhances GLUT4 translocation to the plasma membrane (246). This is further supported by the activation of the tumor suppressor p53, which elevates the levels of GDF15/MIC1 (another candidate listed in **Table 4**), a cytokine known for its appetite-reducing effects and ability to extend lifespan. DHODH inhibition that depletes pyrimidine ribonucleotides is also thought to be responsible for reduced RNA virus replication and decelerated growth in rapidly dividing cells, such as activated T cells and, as just mentioned, cancer cells (243). Interestingly, uridine, a pyrimidine nucleoside present in RNA, has been shown to modulate insulin activity and glycogen synthesis through its interaction with uridine diphosphate (UDP)-glucose (247). The base sequence of the *DHODH* gene is remarkably conserved, with one exception being a prevalent Lys7Gln missense polymorphism (16-72008783-A-C, rs3213422) found in its first exon (248). This variant is found in 75% of individuals in East Asia vs. 34% of individuals in the Middle East (gnomAD) and has been linked with drug (leflunomide) response to rheumatoid arthritis (248–250).

### 3.3 Population-enriched variants with unknown/uncharacterized function

No known effect on gross phenotype or evidence of association with disease has yet been reported among the population-enriched variants identified with the 20 genes listed in **Table 6**. However, a newly released resource, GWAS Central (457), was accessed to provide phenotype associations with a subset of variants in **Table 6**. Further, disease disparities related to the parent gene and/or other variants of the gene were identified and/or the predicted impact of a population-enriched variant on the coded change in protein function were evaluated and listed in **Table 6**.

**Table 6:** Geographic Ancestral Variants with Unknown/Uncharacterized Function.

Gene (SNP)	Affected Transcripts	Name/Function	Population MAF Range	Associated w/ Immunity	Ref	Associated w/ Cancer	Ref	Associated w/ Metabolic Disease	Ref
***ATP10D***		ATPase, class V, type 10D		sphingolipids assoc w/ innate immune response	331	lung cancer	332	controls circulating sphingolipids responsible for atherosclerosis, T2D	333
		transports glucosylceramide, a sphingolipid		downregulated by TGFb in eosinophils	334	colorectal cancer	335		
4-47514685-C-T (rs33995001)	3 (2)	Thr43Ile	0.44 LAT to 0.16 E ASN						
4-47582029-G-A (rs1058793)	4 (1)	Val1240Ile	0.58 E ASN to 0.16 EUR	found in Bulgarian centenarians	336				
4-47591266-G-C (rs4145944)	3 (1)	Ser1389Thr	0.62 AFR/AA to 0.12 E ASN					serum cholesterol	323
		**Disease Disparity:** Sphingolipid levels are elevated in lupus **[** 337 **]** and hepatocellular carcinoma **[** 338 **]**, two diseases with known disparities based on geographic ancestry **[** 339 and 340, respectively]							
***CCDC105***	** **	coiled-coil domain containing 105, tektin like 1, TEKTL1		HBV infection	341	colon, lung cancer	342, 343	interacts with MESD [344], part of WNT pathway in cancer and cardiovascular disease	345, 346
19-15020518-G-A (rs35352238)	1 (1)	Val245Met	0.54 E ASN to 0.11 AFR/AA						
19-15023114-C-A (rs8112667)	1 (1)	Pro499Thr	0.53 E ASN to 0.18 MID E	serum fibrinogen	323				
		**Disease Disparity:** interacts with MAGEA11 **[** 347 **]**, a biomarker for stomach cancer **[** 348 **]**, which shows racial and geographic disparities [reviewed in 349 **]**							
***CRELD2***	** **	cysteine rich with EGF like domains 2		marker in joint infection	350	multiple cancers	350	cardiometabolic disease	350
22-49921715-C-A (rs8139422)	10 (5)	Asp182Glu	0.51 AFR/AA to 0.03 EUR	age-related macular degeneration	351				
		**Disease Disparity:** breast **[** 352 **]** and prostate **[** 353 **]** cancers, reviewed in **[** 60 **]**							
***CSF2RB***		IL3RB, CD131, IL5RB		colony stimulating factor 2 receptor beta surfactant homeostasis	354	variant assoc w leukemia variant assoc w breast cancer	355 356	peptide agonists of EPOR/CD131 heteroreceptor are anti-atherosclerotic	357
22-36930401-G-C (rs16845)	4 (4)	Glu249Gln	0.21 AFR/AA to 0.00 E ASN						
		**Disease Disparity:** breast cancer **[** 60, 352 **]**							
***DNAJB11***	** **	ER-associated DnaJ Hsp40 member B11		immune infiltration in thyroid	358	liver, breast, pancreatic cancer	358	diabetes	119
3-186583914-A-G (rs8147)	3 (2)	Ile264Val	0.47 AFR/AA to 0.15 E ASN	rheumatoid arthritis	359				
		**Disease Disparity:** context-dependent breast cancer **[** 60, 352 **]**							
***DSC1***	** **	desmocollin 1		reduced in pediatric pneumomia	360	head and neck, ovarian, anal	361–363	prevents HDL biogenesis	360
18-31140184-C-T (rs17800159)	2 (2)	ValIle460Ile	0.48 E ASN to 0.03 AFR/AA						
		**Disease Disparity:** ovarian cancer **[** 364 **]**							
***FAM131C***		family with sequence similarity 131 member C		autoimmune target in ApoE KO mice	365	associated with cancer survival	366	upregulated in high fat diet	367
				upregulated in M1 macrophage-rich adipose	368				
1-16058636-C-A (rs1832151)	2 (1)	Ser215Ile	0.33 AFR/AA to 0.0013 E ASN						
1-16060000-C-T (rs71510977)	2 (1)	Arg107Gln	0.78 E ASN to 0.10 AFR/AA						
1-16062531-T-C (rs2863458)	2 (1)	Lys48Glu	0.38 AFR/AA to 0.01 E ASN					waist-hip ratio	324
		**Diseaese Disparity:** interacts with VSNL1 **[** 369 **]**, which is associated with colon cancer **[** 370, 371 **]** and gastric cancer **[** 372 **]**, both cancers that show ethnic disparities **[** 373 **]**							
***ILDR1***	** **	Ig-like domain containing receptor 1		flu virus replication	374	gastric cancer marker	375	diet-induced obesity and hyperglycemia	376
3-121993958-G-C (rs3915061)	5 (3)	Pro264Arg	0.49 S ASN to 0.22 E ASN						
		**Disease Disparity:** gastric cancer [reviewed in 349, 373]							
***ITPRIPL1***	** **	inositol triphosphate interacting protein like 1, KIAA1754L		immune checkpoint inhibition of T-cell activation	377	gene methylation assoc w breast cancer	378	diabetic nephropathy	379
2-96328019-C-T (rs2279105)	4 (4)	Thr463Met	0.69 S ASN to 0.21 E ASN	HbA1c	323				
		**Disease Disparity:** breast cancer **[** 60, 352 **]**							
***PDIA6***		protein disulfide isomerase A6, ERP5 TXNDC7 thioredoxin domain containing 7		lymphoid and myeloid development platelet aggregation and activation	380 381	NSCLC, breast, bladder, gastic, oral, pancreatic cancers	382–387	diabetes	119
2-10790777-T-C (rs4807)	6 (6)	Lys214Arg	0.39 E ASN to 0.125 AFR/AA	serum IgE, HbA1c, rheumatoid arthritis	323, 359			age-related macular degeneration	351
		**Disease Disparity:** breast **[** 60, 352 **]** and gastric cancer [reviewed in 349, 373 **]**							
***RB1***	** **	retinoblastoma 1 transcriptional co-repressor		associated with Treg infiltration in bladder cancer	388	tumor suppressor in multiple cancers	389	negative association with BMI and insulin resistance	390
13-48599402-T-C (rs1887154)	1 (1, non-cannonical)	Leu99Ser (1 transcript)	0.79 AFR/AA to 0.28 MID E						
		**Predicted Impact:** potential phosphorylation site introduced (+Ser): RB1 phosphorylation inactivates this tumor suppessor and promotes tumor progression **[** 389 **],** impacts several regulatory pathways and protein-protein interactions **[** 391 **]**							
***RPAIN***		RPA interacting protein, nuclear transporter, HRIP		variants assoc w/ influenza A virus (RNA) pathogenesis	392 393	alternate splice variants in colon cancer, glioblastoma	394 395	gene expression is associated with BMI	396
17-5422825-C-G (rs12761)	16 (11)	Asn103Lys	0.82 E ASN to 0.22 AFR/AA					BMI	397
		**Disease Disparity:** colon cancer **[** 398 **]**							
***SEMA6D***		sematophorin D6		regulates late phase CD4+ T cells response, anti-inflammatory macrophage polarization	399 400, 401	lung cancer, chemoresponse in breast cancer	151, 402	cardiomyocyte development, immune cell metabolism	401 403
15-47764022-A-G (rs3743279)	9 (9)	Asn307Ser	0.24 AFR/AA to 0.00 EUR	skin pigmentation	404				
15-47765874-G-A (rs532598)	9 (8)	Ser478Asn	0.59 E ASN to 0.34 MID E	partial epilepsies, asthma	405, 406				
		**Disease Disparity:** SEMA6D expression is associated with survival in triple negative breast cancer **[** 407 **]**, which occurs disproportionately among women of African descent **[** 352 **]**							
		**Predicted Impact:** both variants may alter phosphorylation status (+/- Ser) with the potential to alter activity, stability and/or protein-protein interactions							
***SIPA1L2***		signal induced prolif assoc 1 like 2, SPAR2, SPAL2, KIAA1389		assoc w/ H2O2 release from healthy Caucasian lymphoblastoids	408	metastatic clear cell kidney carcinoma (EUR)	409	identified by bioinformatics in type 2 diabetes	410
				inactivates RAP1 (involved in inflammatory response)	410	varying correlation with 23 cancers	411	gene expression assoc with NAFLD	412
1-232403473-C-T (rs2275303)	9 (8)	Gly1639Ser	0.48 E ASN to 0.00 AFR/AA	Alzheimer's in Asian population	413				
1-232439175-T-C (rs2275307)	9 (8)	Thr1322Ala	0.48 E ASN to 0.22 EUR						
		**Disease Disparity:** gene shows highest correlation with cancers with known ethnic disparities **[** 411 **]**, including bladder **[** 414 **]**, esophageal **[** 415 **]**, hepatocellular carcinoma **[** 416 **]**, and ovarian **[** 364 **]**							
		**Predicted Impact:** both variants may alter phosphorylation status (+Ser and -Thr) with the potential to alter activity, stability and/or protein-protein interactions							
***TBC1D4***	** **	AS160 Akt substrate of 160 kD		delivery to chlamydial inclusions	417	breast cancer, multiple myeloma	418, 419	nonsense variant confers insulin resistance and T2D in Greenlandic population	420
13-75286865-A-G (rs557337)	4 (4)	Val1275Ala	0.49 AFR/AA to 0.00 E ASN	fibrinogen	323				
13-75481466-G-A (rs77685055)	3 (3)	Ala101Val	0.29 E ASN to 0.02 AFR/AA	RBC count mean corp. Hb, hematocrit	421–423				
		**Disease Disparity:** gene associated with cancers that show ethnic disparities, including breast **[** 60, 352 **]** and multiple myeloma **[** 424 **]**							
***TESPA1***		HSPC257, thymocyte expressed, positive selection associated 1		development and maturation of T cells TCR regulation	425	pan-cancer prognostic	426	mito-assoc ER mb proteins are assoc w/ cardiovascular disease	427
12-54950349-C-G (rs2171497)	7 (2, non-canonical)	Leu103Phe	0.64 E ASN to 0.05 AFR/AA	ulcerative colitis	428			BMI	397
12-54961249-C-T (rs997173)	8 (5)	Leu496Lys	0.63 E ASN to 0.06 AFR/AA	Kuru and sCJD (prion diseases)	429				
		**Disease Disparity:** although TESPA1 expresssion is upregulated in several cancers, the most dramatic increase in expression occurs in acute myeloid leukemia (AML) **[** 426 **],** a cancer which shows ethnic disparities **[** 430 **]**							
***TVP23C***		TGN vesicle protein 23 homolog C, FAM18B2		gene is an integration site for HBV in liver cancer	431	plasma protein assoc w/ colorectal cancer	432	bioinformatic feature gene assoc w/ ischemic stroke	433
				platelet granule secretion, chronic immune thrombocytopenia	434	data mining prognostic marker for liver cancer	435	readthrough translation with CDRT4 downregulated in obese individuals	436
				associated with CD4 Tex (exhausted T) cells	437	fusion CDRT4 found in pancreatic cancer	438		
17-15502927-A-C (rs73289533)	5 (1)	Ser256Arg	0.24 AFR/AA to 0.00 E ASN						
17-15540420-A-G (rs200768112)	12 (1)	Trp202Arg	0.54 E ASN to 0.28 S ASN			choriocarcinoma	439		
17-15540428-C-G (rs2302252)	12 (1)	Ser199Thr	0.54 E ASN to 0.28 S ASN						
		**Disease Disparity:** Choriocarcinoma incidence rate 10-fold higher in Southeast Asia than in the West **[** 439 **]**							
***ZNF23***	** **	KOX16, ZNF359, ZNF612		correlation with pathogenic environment	49	downregulated in cancer	440	mitochondrial dysregulation in melanoma	441
16-71453303-T-C (rs2070832)	9 (7)	Ser28Gly	0.94 AFR/AA to 0.29 E ASN	partial epilepsies	405			BMI	323
		**Disease Disparity:** reduced expression of this tumor repressor gene in ovarian and endometrial cancers **[** 440 **];** oviarian cancers show ethnic disparities **[** 364 **]**							
		**Predicted Impact:** variant occurs in a putative N-terminal strong transcriptional repressor KRAB domain **[** 442 **]**, loss of Ser may alter activity and/or binding interactions							
		**Note:** ZNF23 KRAB domain is truncated and does not appear to alter repressor activity **[** 440 **]**, however not all ZNF23 interactors (such as mitochondrial ATPAF2. keratin-associated KRTAP10-8, myelin-associated MOBP, growth factor signaling regulators SPRED1 and SPRY1, and TNFR associated adaptor TRAF1), are transcription factors							
***ZNF267***	** **	Zinc Finger Protein 267, HZF2		P. gingivalis infection	443	hepatic, colorectal cancer, B-cell lymphoma	444–446	liver disease (cirhhosis), NAFLD	447, 448
16-31915298-G-A (rs3850114)	2 (1)	Cys350Tyr	1.0 E ASN to 0.61 AFR/AA	serum IgE	323				
		**Disease Disparity:** hepatic and colorectal cancers show ethnic disparities **[** 340, 449 **]**							
***ZNF628***	** **	Zinc Finger Protein 628, ZEC		target gene protamine inhibits microbial infection	450–452	protamine 1 marker for leukemia and colorectal cancer	453, 454	protamine alters BP, mitochondrial function	455
19-55481893-A-G (rs34864744)	2 (2)	Thr234Ala	0.93 AFR/AA to 0.45 E ASN						
		**Disease Disparity:** ethnic disparities observed in colorectal cancers **[** 449 **]**							
		**Predicted Impact:** variant alters potential phosphorylation status (-Thr) in a disordered region of this transcription activator **[** 344 **]** between two zinc finger clusters of the canonical protein that bind DNA independently **[** 456 **]**							

Genes listed have been associated with innate immunity/inflammation, cancer, and cardio-metabolic disease and have at least one variant in the human genome that occurs in at least 20% (Minor Allele Frequency (MAF) ≥ 0.2) of one or more populations. Missense variants are described by their location in the GRCh38 reference genome (accessed from gnomAD v3.1.2), rs number (reference SNP cluster ID), and amino acid location numbers and identities of the original and coded replacement. Populations are defined by Karczewski 2020 (15): African/African American (AFR/AA), East Asian (E ASN), non-Finnish European (EUR), Latino/Latina (LAT), Middle Eastern (MID E), and South Asian (S ASN). The number of affected transcripts listed include total transcripts (first number) and transcripts with missense mutations (in parentheses) that contain the gene variant, but do not include transcripts of any overlapping genes.

#### 3.3.1 Understudied genes SIPA1L2 and TVP23C

Among the 20 genes in **Table 6**, six of these remain understudied, including the exosomal CCDC105/TEKTL1, the putative protein disulfide isomerase CRELD2, the FAM131C protein with unknown function, the putative immune checkpoint ITPRIPL1 membrane protein, the presumptive neural GTPase activator SIPA1L2, and the putative vesicular protein transporter TVP23C. Notably, evidence of an impact on function does exist for one of two population-enriched variants of *SIPA1L2* and one of three population-enriched variants of *TVP23C*. In the case of *SIPA1L2*, both characterized and uncharacterized variants occur at the same high frequency (MAF = 0.48) in East Asians, but Gly1639Ser increases the number of potential phosphorylation sites, whereas Thr1322Ala reduces them, which may result in different functional outcomes (e.g. changes in activation status and/or protein-protein interactions). In both *SIPA1L2* variants, eight of nine possible transcripts code for missense mutations, whereas with *TVP23C*, only in the canonical transcript does the variant result in a missense mutation among five (Ser256Arg) or twelve (Trp202Arg and Ser199Thr) possible isoforms, some of which are read-through fusions with CDRT4 (CMT1A Duplicated Region Transcript 4). It is likely that the *TVP23C* Trp202Arg and Ser199Thr variants commonly co-occur, given their proximity to one another on the gene and their matching frequency distribution, as both have MAFs that range from 0.54 in East Asians to 0.28 in South Asians. Thus, one might speculate that the unknown functional impact of Ser199Thr matches that of Trp202Arg, which was found in a choriocarcinoma patient (458). Notably, choriocarcinoma shows a geographical disparity as it occurs at a ten-fold greater frequency in Southeast Asia than in the West (reviewed in 439). The third *TVP23C* variant Ser256Arg is most common among Africans/African Americans (MAF = 0.24) and involves the loss of a potential phosphorylation site about 50 amino acid residues downstream of the other two *TVP23C* variants.

#### 3.3.2 Additional representative genes of interest

The remaining 14 genes in **Table 6** are better characterized; notably, many have pleiotropic functions beyond the functions initially attributed to them. ATPase Phospholipid Transporting 10D (*ATP10D*) codes for the catalytic subunit of a glycoslyceramide flippase complex at the endoplasmic reticulum (ER), nucleoplasm, and plasma membrane. DnaJ Heat Shock Protein Family (Hsp40) Member B11 (*DNAJB11*) codes for an ER-resident and secreted co-chaperone of BiP/GRP78/HSPA5. Desmocollin 1 (*DSC1*) codes for an adhesive glycoprotein cadherin family member. The Immunoglobulin Like Domain Containing Receptor 1 protein (ILDR1) maintains structural barriers in epithelia and auditory neurosensory hair cells (459), mediates fatty acid and lipoprotein-stimulated cholecystokinin secretion in the small intestine (460), regulates water homeostasis in kidney (461), and interferes with phospholipid scramblase (PLSCR1) anti-viral activity (374). Protein Disulfide Isomerase Family A Member 6 (PDIA6) inhibits intracellular aggregation of misfolded proteins and extracellular aggregation of platelets (381). Replication Protein A Interacting Protein (RPAIN) participates in DNA metabolism, nuclear import, and response to UV light. The Semaphorin 6D (*SEMA6D*) gene codes for an integral membrane protein member of the semaphorin family whose members collectively sculpt axonal paths, branches, conduction, and target selection; the distribution of nine *SEMA6D* transcript isoforms varies according to developmental stage and tissue type. Tre-2/BUB2/CDC16 (TBC) Domain Family Member 4 (TBC1D4, also referred to as Akt Substrate of 160 kD or AS160) is a Rab-GTPase activator with multiple transcript variants; isoform 2 promotes SLC2A4/GLUT4 presentation at the plasma membrane to increase cellular glucose uptake (344). Thymocyte Expressed, Positive Selection Associated 1 (*TESPA1*) interacts with COP9 and TCR signalsomes and participates in T cell differentiation and T cell receptor signaling. Three zinc finger (ZNF) proteins ZNF23, ZNF267, and ZNF628 localize to the nucleus and regulate transcription. Parent genes and the corresponding population-enriched variants of the common cytokine receptor beta chain *CSF2RB* and the transcription co-repressor *RB1* are both discussed below.

##### 3.3.2.1 CSF2RB

Colony stimulating factor 2 receptor beta (CSF2RB, CD131) forms dimers with the alpha receptor subunits for cytokines IL-3, IL-5, and GM-CSF (CSF2). As noted above, a population-enriched variant of the IL3RA subunit also exists, although the population distributions of these two variants are very different: the Val323Leu *IL3RA* variant is found least frequently among Africans/African Americans (MAF = 0.06, **Table 4**), whereas the Glu249Gln *CSF2RB* variant is more predominant in Africans/African Americans than any other population (MAF = 0.21).

*CSF2RB* is associated with pulmonary alveolar proteinosis (PAP), which involves the accumulation of surfactant and macrophage dysfunction in alveoli (reviewed in 462). Although studies so far have not suggested geographic or population differences in PAP occurrence, the most common PAP co-morbidities include cardiovascular disease, type 2 diabetes, and hypertension, all of which are unevenly distributed among populations. Further, a rare Arg461Cys *CSF2RB* variant (MAF< 0.001, not listed in **Table 6**) was found in individual patients with leukemia (355) and breast cancer (356). Notably, both of these cancers show racial and ethnic disparities [430 and 352 respectively].

##### 3.3.2.2 RB1

Retinoblastoma (RB1) was one of the first tumor suppressors to be identified. Alterations in the expression and sequence of the *RB1* gene have been implicated in several cancers besides retinoblastoma where they were originally characterized (reviewed in 391). More than 40 years of extensive research indicates that regulation of and by RB1 is highly complex, linked with multiple signaling pathways, and varies with context. Not surprisingly, the number of proteins shown to interact with RB1 is more than 30 as curated by UniProt (344) and more than 150 as curated in BioGRID (463) and IntAct (464). The functional diversity of the binding partners of RB1 is consistent with its pleiotropic effects, which extend beyond transcription and cell cycle control to include progenitor maturation, terminal differentiation, and immune evasion (391).

Five protein coding transcripts of *RB1* have been identified. These include 1) the MANE select (canonical) protein composed of 27 exons encoding a total of 928 aa residues; 2) a closely related transcript that is 5 as shorter and differs from the canonical protein by 18 of its last 19 C-terminal residues; and 3) three much shorter transcripts (coding for 53, 103 or 110 aa peptides) which include all or portions of only 2 or 3 exons of the canonical protein. Of these shorter transcripts, the two shortest are derived from the N-terminal portion of RB1. In contrast, the 110 aa non-canonical transcript codes for an unidentified N-terminal residue equivalent to the Ser501 residue of the canonical protein and then aligns with all canonical residues up through Ser565; the remaining non-canonical aa residues 66-110 are located downstream of the canonical C-terminal residue 928. It is in this extra-exonic portion of the non-canonical 110 aa *RB1* isoform that the Leu99Ser population-enriched variant, which introduces a potential phosphorylation site, is found. In spite of the high number of aa residues (n ≥ 105) in the canonical RB1 protein that are known to be post-translationally modified, within the aa 501-565 residue range that overlaps with the first 65 residues of the 110 aa isoforms, only two potential ubiquitination sites have been identified in the vicinity of aa 550) (391).

## 4 Conclusion

Population studies have traditionally focused on querying individual diseases or combinations of diseases, including cancer and cardio-metabolic disease, which frequently show disparate prevalence and/or severity in non-European populations. In this perspective, we have introduced a complementary approach that explores the intersection of innate immunity, cancer, and cardio-metabolic diseases. The effective elimination of disease disparities will involve not only addressing the profound social and behavioral determinants of health, but also identifying and treating the biological contributors of disease that include novel genes as well as previously characterized genes that participate in novel pathways.

We suggest that careful evaluation of population differences in conventional and unconventional innate immune genes and their related pathways will provide key insights into the underlying mechanisms that connect cancer and cardio-metabolic diseases. At the same time, the genes we have identified in this study that are associated with both cancer and cardio-metabolic diseases may play critical roles in under-appreciated facets of innate immunity and their contribution to disease disparities. Further, we predict that the geographic ancestral distribution of innate immune gene variants will match the geographical distribution of the environmental stressors (including but not limited to infectious agents) that they are designed to mitigate as described above for *HbS* and *DARC* variants with malaria (Section 1.3).

The genes we have identified serve as potential targets for diagnostics and/or therapeutic interventions. Notably, the development and clinical use of therapeutics targeting these candidate genes is likely to require a nuanced approach since variations in these genes across different global populations are likely to alter the activity and/or expression of their coded proteins, with the subsequent potential to impact therapeutic outcomes. Assessing the prevalence of specific target variants in one or more major populations and, more precisely, the presence of these specific target variants in individuals is a consequential step towards increasing the safety and effectiveness of emerging therapies. This perspective highlights the importance of 1) considering genetic diversity in identifying and developing treatments and 2) continuing to incorporate ongoing GWAS projects as they identify and characterize new or understudied genes and their population-enriched variants associated with complex and infectious diseases.

## Author contributions

SY: Conceptualization, Data curation, Writing – original draft. DH: Data curation, Writing – original draft. KW: Data curation, Writing – original draft. NL: Writing – review & editing. KSK: Conceptualization, Funding acquisition, Supervision, Writing – review & editing.

## References

[1] Alvidrez J Castille D Laude-Sharp M Rosario A Tabor D. The national institute on minority health and health disparities research framework. Am J Public Health (2019) 109(S1):S16–20. doi: 10.2105/AJPH.2018.304883 PMC6356129 30699025

[2] Lutz R. Health Disparities Among African-Americans (2022). Available at: https://www.pfizer.com/news/articles/health_disparities_among_african_americans.

[3] Deshmukh SK Azim S Ahmad A Zubair H Tyagi N Srivastava SK. Biological basis of cancer health disparities: resources and challenges for research. Am J Cancer Res (2017) 7(1):1–12. 28123843 PMC5250676

[4] Zhang R Sun J Wang C Wang X Zhao P Yuan Y. The racial disparities in the epidemic of metabolic syndrome with increased age: A study from 28,049 Chinese and American adults. Front Public Health (2021) 9:797183. doi: 10.3389/fpubh.2021.797183 35178373 PMC8843927

[5] Cullen MR Lemeshow AR Russo LJ Barnes DM Ababio Y Habtezion A. Disease-specific health disparities: A targeted review focusing on race and ethnicity. Healthc (Basel) (2022) 10(4):603. doi: 10.3390/healthcare10040603 PMC9025451 35455781

[6] American Cancer Society (ACS). Cancer Facts & Figures 2022. Atlanta: American Cancer Society (2022).

[7] Heron M. Deaths: Leading causes for 2018. Hyattsville, MD: Center for Disease Control (2021). 34029179

[8] Pham C Fong TL Zhang J Liu L. Striking racial/ethnic disparities in liver cancer incidence rates and temporal trends in California 1988-2012. J Natl Cancer Inst (2018) 110(11):1259–69. doi: 10.1093/jnci/djy051 PMC7191878 29617913

[9] Zhang CH Cheng Y Zhang S Fan J Gao Q. Changing epidemiology of hepatocellular carcinoma in Asia. Liver Int (2022) 42(9):2029–41. doi: 10.1111/liv.15251 35319165

[10] Salvatore M Jeon J Meza R. Changing trends in liver cancer incidence by race/ethnicity and sex in the US: 1992-2016. Cancer Causes Control (2019) 30(12):1377–88. doi: 10.1007/s10552-019-01237-4 31606852

[11] Ellington T Miller J Henley S Wilson R Wu M Richardson L. Trends in breast cancer incidence, by race, ethnicity, and age among women aged ≥20 years — United States 1999–2018. Morbidity Mortality Weekly Rep (MMWR) (2022). doi: 10.15585/mmwr.mm7102a2 PMC8757618 35025856

[12] Hashim D Boffetta P La Vecchia C Rota M Bertuccio P Malvezzi M. The global decrease in cancer mortality: trends and disparities. Ann Oncol (2016) 27(5):926–33. doi: 10.1093/annonc/mdw027 26802157

[13] Kosoy R Nassir R Tian C White PA Butler LM Silva G. Ancestry informative marker sets for determining continental origin and admixture proportions in common populations in America. Hum Mutat (2009) 30(1):69–78. doi: 10.1002/humu.20822 18683858 PMC3073397

[14] Shriner D. Overview of admixture mapping. Curr Protoc (2023) 3(2):e677. doi: 10.1002/cpz1.677 36786670

[15] Karczewski KJ Francioli LC Tiao G Cummings BB Alföldi J Wang Q. The mutational constraint spectrum quantified from variation in 141,456 humans. Nature (2020) 581(7809):434–43. doi: 10.1038/s41586-020-2308-7 PMC7334197 32461654

[16] National Academies of Sciences, Engineering, and Medicine Division of Behavioral and Social Sciences and Education Health and Medicine Division Committee on Population Board on Health Sciences Policy Committee on the Use of Race, Ethnicity, and Ancestry as Population Descriptors in Genomics Research. 1, Population Descriptors in Human Genetics Research: Genesis, Evolution, and Challenges. In: Using Population Descriptors in Genetics and Genomics Research: A New Framework for an Evolving Field. Washington (DC: National Academies Press (US (2023). Available at: https://www.ncbi.nlm.nih.gov/books/NBK592850/. 36989389

[17] Olden K White SL. Health-related disparities: influence of environmental factors. Med Clinics North America (2005) 89(4):721–38. doi: 10.1016/j.mcna.2005.02.001 15925646

[18] Tung EL Cagney KA Peek ME Chin MH. Spatial context and health inequity: reconfiguring race, place, and poverty. J Urban Health (2017) 94(6):757–63. doi: 10.1007/s11524-017-0210-x PMC5722735 29134325

[19] Ruiz D Becerra M Jagai JS Ard K Sargis RM. Disparities in environmental exposures to endocrine-disrupting chemicals and diabetes risk in vulnerable populations. Diabetes Care (2018) 41:193–205. doi: 10.2337/dc16-2765 29142003 PMC5741159

[20] Vitlic A Lord JM Phillips AC. Stress, ageing and their influence on functional, cellular and molecular aspects of the immune system. Age (Dordr) (2014) 36(3):9631. doi: 10.1007/s11357-014-9631-6 24562499 PMC4082590

[21] Borrell LN Rodriguez-Alvarez E Dallo FJ. Racial/ethnic inequities in the associations of allostatic load with all-cause and cardiovascular-specific mortality risk in US adults. PloS One (2020) 15(2):e0228336. doi: 10.1371/journal.pone.0228336 32053626 PMC7018050

[22] Van Dyke ME Baumhofer NK Slopen N Mujahid MS Clark CR Williams DR. Pervasive discrimination and allostatic load in African American and white adults. Psychosom Med (2020) 82(3):316–23. doi: 10.1097/PSY.0000000000000788 PMC7138744 32108740

[23] Miller HN LaFave S Marineau L Stephens J Thorpe RJ. The impact of discrimination on allostatic load in adults: An integrative review of literature. J Psychosomatic Res (2021) 146:110434. doi: 10.1016/j.jpsychores.2021.110434 PMC8172431 33810863

[24] Fine MJ Ibrahim SA Thomas SB. The role of race and genetics in health disparities research. Am J Public Health (2005) 95(12):2125–8. doi: 10.2105/AJPH.2005.076588 PMC1449495 16257933

[25] Scharff DP Mathews KJ Jackson P Hoffsuemmer J Martin E Edwards D. More than Tuskegee: understanding mistrust about research participation. J Health Care Poor Underserved (2010) 21(3):879–97. doi: 10.1353/hpu.0.0323 PMC4354806 20693733

[26] 2022 National Healthcare Quality and Disparities Report. Rockville, MD: Agency for Healthcare Research and Quality (2022). Available at: https://www.ahrq.gov/research/findings/nhqrdr/nhqdr22/index.html. 36475568

[27] Dutil J Chen Z Monteiro AN Teer JK Eschrich SA. An interactive resource to probe genetic diversity and estimated ancestry in cancer cell lines. Cancer Res (2019) 79(7):1263–73. doi: 10.1158/0008-5472.CAN-18-2747 PMC6445675 30894373

[28] Hooker SE Jr. Woods-Burnham L Bathina M Lloyd S Gorjala P Mitra R. Genetic ancestry analysis reveals misclassification of commonly used cancer cell lines. Cancer Epidemiol Biomarkers Prev (2019) 28(6):1003–9. doi: 10.1158/1055-9965.EPI-18-1132 PMC6548687 30787054

[29] Sirugo G Williams SM Tishkoff SA. The missing diversity in human genetic studies. Cell (2019) 177(1):26–31. doi: 10.1016/j.cell.2019.02.048 30901543 PMC7380073

[30] Cavazzoni P Anagnostiadis E Lolic M. 2020 Drug Trials Snapshots Summary Report. Center for Drug Evaluation and Research (CDER), editor. Silver Spring, MD: Administration USFaD (20b21). Available at: www.fda.gov.

[31] Popejoy AB Fullerton SM. Genomics is failing on diversity. Nature (2016) 538:161–4. doi: 10.1038/538161a PMC5089703 27734877

[32] Rajman I Knapp L Morgan T Masimirembwa C. African genetic diversity: implications for cytochrome P450-mediated drug metabolism and drug development. EBioMedicine (2017) 17:67–74. doi: 10.1016/j.ebiom.2017.02.017 28237373 PMC5360579

[33] Denny JC Collins FS. Precision medicine in 2030-seven ways to transform healthcare. Cell (2021) 184(6):1415–9. doi: 10.1016/j.cell.2021.01.015 PMC9616629 33740447

[34] Collins FS Adams AB Aklin C Archer TK Bernard MA Boone E. Affirming NIH’s commitment to addressing structural racism in the biomedical research enterprise. Cell (2021) 184(12):3075–9. doi: 10.1016/j.cell.2021.05.014 34115967

[35] Lek M Karczewski KJ Minikel EV Samocha KE Banks E Fennell T. Analysis of protein-coding genetic variation in 60,706 humans. Nature (2016) 536(7616):285–91. doi: 10.1038/nature19057 PMC5018207 27535533

[36] Witherspoon DJ Wooding S Rogers AR Marchani EE Watkins WS Batzer MA. Genetic similarities within and between human populations. Genetics (2007) 176(1):351–9. doi: 10.1534/genetics.106.067355 PMC1893020 17339205

[37] Ahsan T Urmi NJ Sajib AA. Heterogeneity in the distribution of 159 drug-response related SNPs in world populations and their genetic relatedness. PloS One (2020) 15(1):e0228000. doi: 10.1371/journal.pone.0228000 31971968 PMC6977754

[38] Burroughs VJ Maxey RW Levy RA. Racial and ethnic differences in response to medicines: towards individualized pharmaceutical treatment. J Natl Med Assoc (2002) 94(10 Suppl):1–26. PMC2594139 12401060

[39] Johnson JA. Ethnic differences in cardiovascular drug response: potential contribution of pharmacogenetics. Circulation (2008) 118(13):1383–93. doi: 10.1161/CIRCULATIONAHA.107.704023 PMC2730023 18809808

[40] De T Park CS Perera MA. Cardiovascular pharmacogenomics: does it matter if you’re black or white? Annu Rev Pharmacol Toxicol (2019) 59:577–603. doi: 10.1146/annurev-pharmtox-010818-021154 30296897 PMC7482366

[41] Kwiatkowski DP. How malaria has affected the human genome and what human genetics can teach us about malaria. Am J Hum Genet (2005) 77(2):171–92. doi: 10.1086/432519 PMC1224522 16001361

[42] Sirugo G Hennig BJ Adeyemo AA Matimba A Newport MJ Ibrahim ME. Genetic studies of African populations: an overview on disease susceptibility and response to vaccines and therapeutics. Hum Genet (2008) 123(6):557–98. doi: 10.1007/s00439-008-0511-y 18512079

[43] Wang J Ou ZL Hou YF Luo JM Shen ZZ Ding J. Enhanced expression of Duffy antigen receptor for chemokines by breast cancer cells attenuates growth and metastasis potential. Oncogene (2006) 25(54):7201–11. doi: 10.1038/sj.onc.1209703 16785997

[44] Mangano VD Modiano D. An evolutionary perspective of how infection drives human genome diversity: the case of malaria. Curr Opin Immunol (2014) 30:39–47. doi: 10.1016/j.coi.2014.06.004 24996199

[45] Genovese G Friedman DJ Ross MD Lecordier L Uzureau P Freedman BI. Association of trypanolytic apoL1 variants with kidney disease in African-Americans. Science (2010) 329(5993):841–5. doi: 10.1126/science.1193032 PMC2980843 20647424

[46] Freedman BI Limou S Ma L Kopp JB. APOL1-associated nephropathy: A key contributor to racial disparities in CKD. Am J Kidney Dis (2018) 72(5 Suppl 1):S8–S16. doi: 10.1053/j.ajkd.2018.06.020 30343724 PMC6200346

[47] Quintana-Murci L. Human immunology through the lens of evolutionary genetics. Cell (2019) 177(1):184–99. doi: 10.1016/j.cell.2019.02.033 30901539

[48] Deschamps M Laval G Fagny M Itan Y Abel L Casanova JL. Genomic signatures of selective pressures and introgression from archaic hominins at human innate immunity genes. Am J Hum Genet (2016) 98(1):5–21. doi: 10.1016/j.ajhg.2015.11.014 26748513 PMC4716683

[49] Fumagalli M Sironi M Pozzoli U Ferrer-Admetlla A Pattini L Nielsen R. Signatures of environmental genetic adaptation pinpoint pathogens as the main selective pressure through human evolution. PloS Genet (2011) 7(11):e1002355. doi: 10.1371/journal.pgen.1002355 22072984 PMC3207877

[50] Barreiro LB Quintana-Murci L. From evolutionary genetics to human immunology: how selection shapes host defence genes. Nat Rev Genet (2010) 11(1):17–30. doi: 10.1038/nrg2698 19953080

[51] Piel FB Patil AP Howes RE Nyangiri OA Gething PW Williams TN. Global distribution of the sickle cell gene and geographical confirmation of the malaria hypothesis. Nat Commun (2010) 1:104. doi: 10.1038/ncomms1104 21045822 PMC3060623

[52] Howes RE Patil AP Piel FB Nyangiri OA Kabaria CW Gething PW. The global distribution of the Duffy blood group. Nat Commun (2011) 2:266. doi: 10.1038/ncomms1265 21468018 PMC3074097

[53] Karlsson EK Kwiatkowski DP Sabeti PC. Natural selection and infectious disease in human populations. Nat Rev Genet (2014) 15(6):379–93. doi: 10.1038/nrg3734 PMC4912034 24776769

[54] Nedelec Y Sanz J Baharian G Szpiech ZA Pacis A Dumaine A. Genetic ancestry and natural selection drive population differences in immune responses to pathogens. Cell (2016) 167(3):657–669 e621. doi: 10.1016/j.cell.2016.09.025 27768889

[55] Nahid P Jarlsberg LG Kato-Maeda M Segal MR Osmond DH Gagneux S. Interplay of strain and race/ethnicity in the innate immune response to M. tuberculosis. PloS One (2018) 13(5):e0195392. doi: 10.1371/journal.pone.0195392 29787561 PMC5963792

[56] Yao S Hong CC Ruiz-Narvaez EA Evans SS Zhu Q Schaefer BA. Genetic ancestry and population differences in levels of inflammatory cytokines in women: Role for evolutionary selection and environmental factors. PloS Genet (2018) 14(6):e1007368. doi: 10.1371/journal.pgen.1007368 29879116 PMC5991662

[57] Li Y Oosting M Smeekens SP Jaeger M Aguirre-Gamboa R Le KTT. A functional genomics approach to understand variation in cytokine production in humans. Cell (2016) 167(4):1099–1110 e1014. doi: 10.1016/j.cell.2016.10.017 27814507

[58] Hanahan D Weinberg RA. Hallmarks of cancer: the next generation. Cell (2011) 144:646–74. doi: 10.1016/j.cell.2011.02.013 21376230

[59] Hanahan D. Hallmarks of cancer: new dimensions. Cancer Discovery (2022) 12(1):31–46. doi: 10.1158/2159-8290.CD-21-1059 35022204

[60] Yeyeodu ST Kidd LR Kimbro KS. Protective innate immune variants in racial/ethnic disparities of breast and prostate cancer. Cancer Immunol Res (2019) 7(9):1384–9. doi: 10.1158/2326-6066.CIR-18-0564 PMC6730653 31481520

[61] Chen CH Lu YS Cheng AL Huang CS Kuo WH Wang MY. Disparity in tumor immune microenvironment of breast cancer and prognostic impact: Asian versus Western populations. Oncologist (2020) 25(1):e16–23. doi: 10.1634/theoncologist.2019-0123 PMC6964121 31371522

[62] Zajac KK Malla S Babu RJ Raman D Tiwari AK. Ethnic disparities in the immune microenvironment of triple negative breast cancer and its role in therapeutic outcomes. Cancer Rep (2023) Suppl 1(Suppl 1):e1779. doi: 10.1002/cnr2.1779 PMC10440847 36632988

[63] Chaudhary S Ganguly K Muniyan S Pothuraju R Sayed Z Jones DT. Immunometabolic alterations by HPV infection: new dimensions to head and neck cancer disparity. JNCI: J Natl Cancer Institute (2019) 111(3):233–44. doi: 10.1093/jnci/djy207 PMC6410958 30615137

[64] Chaudhary S Dam V Ganguly K Sharma S Atri P Chirravuri-Venkata R. Differential mutation spectrum and immune landscape in African Americans versus Whites: A possible determinant to health disparity in head and neck cancer. Cancer Lett (2020) 492:44–53. doi: 10.1016/j.canlet.2020.07.029 32738272 PMC8432304

[65] Guerrero-Preston R Lawson F Rodriguez-Torres S Noordhuis MG Pirini F Manuel L. JAK3 variant, immune signatures, DNA methylation, and social determinants linked to survival racial disparities in head and neck cancer patients. Molecular disparities in black and non-latino white HNSCC. Cancer Prev Res (2019) 12(4):255–70. doi: 10.1158/1940-6207.CAPR-17-0356 PMC6800037 30777857

[66] Byrne CA Gomez SL Kim S Oddo VM Koh TJ Fantuzzi G. Disparities in inflammation between non-Hispanic black and white individuals with lung cancer in the Greater Chicago Metropolitan area. Front Immunol (2022) 13:1008674. doi: 10.3389/fimmu.2022.1008674 36544783 PMC9760905

[67] Xu Y Zhang L Thaiparambil J Mai S Perera DN Zhang J. Patients with lung cancer of different racial backgrounds harbor distinct immune cell profiles. Cancer Res Commun (2022) 2(8):884–93. doi: 10.1158/2767-9764.CRC-22-0057 PMC10010305 36923308

[68] Curran T Sun Z Gerry B Findlay VJ Wallace K Li Z. Differential immune signatures in the tumor microenvironment are associated with colon cancer racial disparities. Cancer Med (2021) 10(5):1805–14. doi: 10.1002/cam4.3753 PMC7940243 33560598

[69] Ahmad S Ashktorab H Brim H Housseau F. Inflammation, microbiome and colorectal cancer disparity in African-Americans: Are there bugs in the genetics? World J Gastroenterol (2022) 28(25):2782. doi: 10.3748/wjg.v28.i25.2782 35978869 PMC9280725

[70] Kiely M Lord B Ambs S. Immune response and inflammation in cancer health disparities. Trends Cancer (2022) 8(4):316–27. doi: 10.1016/j.trecan.2021.11.010 PMC8930618 34965905

[71] Kalyanaraman B. Exploiting the tumor immune microenvironment and immunometabolism using mitochondria-targeted drugs: Challenges and opportunities in racial disparity and cancer outcome research. FASEB J (2022) 36(4):e22226. doi: 10.1096/fj.202101862R 35233843 PMC9242412

[72] Younossi Z Anstee QM Marietti M Hardy T Henry L Eslam M. Global burden of NAFLD and NASH: trends, predictions, risk factors and prevention. Nat Rev Gastroenterol Hepatol (2018) 15(1):11–20. doi: 10.1038/nrgastro.2017.109 28930295

[73] Saeedi P Petersohn I Salpea P Malanda B Karuranga S Unwin N. Global and regional diabetes prevalence estimates for 2019 and projections for 2030 and 2045: Results from the International Diabetes Federation Diabetes Atlas, 9(th) edition. Diabetes Res Clin Pract (2019) 157:107843. doi: 10.1016/j.diabres.2019.107843 31518657

[74] Malik VS Willet WC Hu FB. Nearly a decade on - trends, risk factors and policy implications in global obesity. Nat Rev Endocrinol (2020) 16(11):615–6. doi: 10.1038/s41574-020-00411-y PMC7461756 32873971

[75] Huang PL. A comprehensive definition for metabolic syndrome. Dis Model Mech (2009) 2(5-6):231–7. doi: 10.1242/dmm.001180 PMC2675814 19407331

[76] Rochlani Y Pothineni NV Kovelamudi S Mehta JL. Metabolic syndrome: pathophysiology, management, and modulation by natural compounds. Ther Adv Cardiovasc Dis (2017) 11(8):215–25. doi: 10.1177/1753944717711379 PMC5933580 28639538

[77] Godoy-Matos AF Silva Junior WS Valerio CM. NAFLD as a continuum: from obesity to metabolic syndrome and diabetes. Diabetol Metab Syndr (2020) 12:60. doi: 10.1186/s13098-020-00570-y 32684985 PMC7359287

[78] Grundy SM Cleeman JI Daniels SR Donato KA Eckel RH Franklin BA. Diagnosis and management of the metabolic syndrome: an American Heart Association/National Heart, Lung, and Blood Institute Scientific Statement. Circulation (2005) 112(17):2735–52. doi: 10.1161/CIRCULATIONAHA.105.169404 16157765

[79] Chan KL Cathomas F Russo SJ. Central and peripheral inflammation link metabolic syndrome and major depressive disorder. Physiol (Bethesda Md.) (2019) 34(2):123–33. doi: 10.1152/physiol.00047.2018 PMC6586832 30724127

[80] Akinyemiju T Do AN Patki A Aslibekyan S Zhi D Hidalgo B. Epigenome-wide association study of metabolic syndrome in African-American adults. Clin Epigenet (2018) 10:49. doi: 10.1186/s13148-018-0483-2 PMC5891946 29643945

[81] Al Rifai M Silverman MG Nasir K Budoff MJ Blankstein R Szklo M. The association of nonalcoholic fatty liver disease, obesity, and metabolic syndrome, with systemic inflammation and subclinical atherosclerosis: The Multi-Ethnic Study of Atherosclerosis (MESA). Atherosclerosis (2015) 239(2):629–33. doi: 10.1016/j.atherosclerosis.2015.02.011 PMC4406399 25683387

[82] Hotamisligil GS. Inflammation, metaflammation and immunometabolic disorders. Nature (2017) 542(7640):177–85. doi: 10.1038/nature21363 28179656

[83] Denis GV Sebastiani P Bertrand KA Strissel KJ Tran AH Slama J. Inflammatory signatures distinguish metabolic health in African American women with obesity. PloS One (2018) 13(5):e0196755. doi: 10.1371/journal.pone.0196755 29738558 PMC5940209

[84] Berbudi A Rahmadika N Tjahjadi AI Ruslami R. Type 2 diabetes and its impact on the immune system. Curr Diabetes Rev (2020) 16(5):442–9. doi: 10.2174/1573399815666191024085838 PMC7475801 31657690

[85] Brestoff JR Artis D. Immune regulation of metabolic homeostasis in health and disease. Cell (2015) 161(1):146–60. doi: 10.1016/j.cell.2015.02.022 PMC4400287 25815992

[86] Adolph TE Grander C Grabherr F Tilg H. Adipokines and non-alcoholic fatty liver disease: multiple interactions. Int J Mol Sci (2017) 18(8):1649. doi: 10.3390/ijms18081649 28758929 PMC5578039

[87] Zatterale F Longo M Naderi J Raciti GA Desiderio A Miele C. Chronic adipose tissue inflammation linking obesity to insulin resistance and type 2 diabetes. Front Physiol (2019) 10:1607. doi: 10.3389/fphys.2019.01607 32063863 PMC7000657

[88] Wu H Ballantyne CM. Metabolic inflammation and insulin resistance in obesity. Circ Res (2020) 126(11):1549–64. doi: 10.1161/CIRCRESAHA.119.315896 PMC7250139 32437299

[89] Coats BR Schoenfelt KQ Barbosa-Lorenzi VC Peris E Cui C Hoffman A. Metabolically activated adipose tissue macrophages perform detrimental and beneficial functions during diet-induced obesity. Cell Rep (2017) 20(13):3149–61. doi: 10.1016/j.celrep.2017.08.096 PMC5646237 28954231

[90] Li Y Yun K Mu R. A review on the biology and properties of adipose tissue macrophages involved in adipose tissue physiological and pathophysiological processes. Lipids Health Dis (2020) 19(1):164. doi: 10.1186/s12944-020-01342-3 32646451 PMC7350193

[91] Tong X Wei L Wang T Han R. Remodeling of Macrophages in White Adipose Tissue under the Conditions of Obesity as well as Lipolysis. Oxid Med Cell Longev (2021) 2021:9980877. doi: 10.1155/2021/9980877 34504646 PMC8423577

[92] Gao B. Basic liver immunology. Cell Mol Immunol (2016) 13(3):265–6. doi: 10.1038/cmi.2016.09 PMC4856812 27041634

[93] Robinson MW Harmon C O’Farrelly C. Liver immunology and its role in inflammation and homeostasis. Cell Mol Immunol (2016) 13(3):267–76. doi: 10.1038/cmi.2016.3 PMC4856809 27063467

[94] Kubes P Jenne C. Immune responses in the liver. Annu Rev Immunol (2018) 36:247–77. doi: 10.1146/annurev-immunol-051116-052415 29328785

[95] Gonzalez FB Villar SR Toneatto J Pacini MF Marquez J D’Attilio L. Immune response triggered by Trypanosoma cruzi infection strikes adipose tissue homeostasis altering lipid storage, enzyme profile and adipokine expression. Med Microbiol Immunol (2019) 208(5):651–66. doi: 10.1007/s00430-018-0572-z 30413884

[96] Fiorucci S Biagioli M Zampella A Distrutti E. Bile acids activated receptors regulate innate immunity. Front Immunol (2018) 9:1853. doi: 10.3389/fimmu.2018.01853 30150987 PMC6099188

[97] Guo C Xie S Chi Z Zhang J Liu Y Zhang L. Bile acids control inflammation and metabolic disorder through inhibition of NLRP3 inflammasome. Immunity (2016) 45(4):802–16. doi: 10.1016/j.immuni.2016.09.008 27692610

[98] Chen ML Takeda K Sundrud MS. Emerging roles of bile acids in mucosal immunity and inflammation. Mucosal Immunol (2019) 12(4):851–61. doi: 10.1038/s41385-019-0162-4 30952999

[99] Camilleri M. Bile acid detergency: permeability, inflammation, and effects of sulfation. Am J Physiol Gastrointest Liver Physiol (2022) 322(5):G480–8. doi: 10.1152/ajpgi.00011.2022 PMC8993532 35258349

[100] Chang YW Hung LC Chen YC Wang WH Lin CY Tzeng HH. Insulin reduces inflammation by regulating the activation of the NLRP3 inflammasome. Front Immunol (2020) 11:587229. doi: 10.3389/fimmu.2020.587229 33679687 PMC7933514

[101] Jin C Henao-Mejia J Flavell RA. Innate immune receptors: key regulators of metabolic disease progression. Cell Metab (2013) 17(6):873–82. doi: 10.1016/j.cmet.2013.05.011 23747246

[102] Burhans MS Hagman DK Kuzma JN Schmidt KA Kratz M. Contribution of adipose tissue inflammation to the development of type 2 diabetes mellitus. Compr Physiol (2018) 9(1):1–58. doi: 10.1002/cphy.c170040 30549014 PMC6557583

[103] Bernardi S Marcuzzi A Piscianz E Tommasini A Fabris B. The complex interplay between lipids, immune system and interleukins in cardio-metabolic diseases. Int J Mol Sci (2018) 19(12):4058. doi: 10.3390/ijms19124058 30558209 PMC6321433

[104] Pirillo A Bonacina F Norata GD Catapano AL. The interplay of lipids, lipoproteins, and immunity in atherosclerosis. Curr Atheroscler Rep (2018) 20(3):12. doi: 10.1007/s11883-018-0715-0 29445885

[105] Prohaska A Racimo F Schork AJ Sikora M Stern AJ Ilardo M. Human disease variation in the light of population genomics. Cell (2019) 177(1):115–31. doi: 10.1016/j.cell.2019.01.052 30901534

[106] Uffelmann E Huang QQ Munung NS de Vries J Okada Y Martin AR. Genome-wide association studies. Nat Rev Methods Primers (2021) 1(1):59. doi: 10.1038/s43586-021-00056-9

[107] Gudmundsson S Singer-Berk M Watts NA Phu W Goodrich JK Solomonson M. Variant interpretation using population databases: Lessons from gnomAD. Hum Mutat (2022) 43(8):1012–30. doi: 10.1002/humu.24309 PMC9160216 34859531

[108] Pennisi E. ENCODE project writes eulogy for junk DNA. Science (2012) 337(6099):1159–61. doi: 10.1126/science.337.6099.115 22955811

[109] Salzberg SL. Open questions: How many genes do we have? BMC Biol (2018) 16(1):94. doi: 10.1186/s12915-018-0564-x 30124169 PMC6100717

[110] Maurano MT Humbert R Rynes E Thurman RE Haugen E Wang H. Systematic localization of common disease-associated variation in regulatory DNA. Science (2012) 337(6099):1190–5. doi: 10.1126/science.1222794 PMC3771521 22955828

[111] Sharma Y Miladi M Dukare S Boulay K Caudron-Herger M Gross M. A pan-cancer analysis of synonymous mutations. Nat Commun (2019) 10(1):2569. doi: 10.1038/s41467-019-10489-2 31189880 PMC6562042

[112] Walsh IM Bowman MA Soto Santarriaga IF Rodriguez A Clark PL. Synonymous codon substitutions perturb cotranslational protein folding in *vivo* and impair cell fitness. Proc Natl Acad Sci U.S.A. (2020) 117(7):3528–34. doi: 10.1073/pnas.1907126117 PMC7035613 32015130

[113] Herreros E Janssens X Pepe D Keersmaecker KD. SNPs ability to influence disease risk: breaking the silence on synonymous mutations in cancer. In: Single Nucleotide Polymorphisms. (Cham, Switzerland: Springer) (2022). p. 77–96.

[114] Velloso FJ Campos AR Sogayar MC Correa RG. Proteome profiling of triple negative breast cancer cells overexpressing NOD1 and NOD2 receptors unveils molecular signatures of Malignant cell proliferation. BMC Genomics (2019) 20(1):152. doi: 10.1186/s12864-019-5523-6 30791886 PMC6385390

[115] Mackinnon MJ Ndila C Uyoga S Macharia A Snow RW Band G. Environmental correlation analysis for genes associated with protection against malaria. Mol Biol Evol (2016) 33(5):1188–204. doi: 10.1093/molbev/msw004 PMC4839215 26744416

[116] Liang B Ding H Huang L Luo H Zhu X. GWAS in cancer: progress and challenges. Mol Genet Genomics (2020) 295(3):537–61. doi: 10.1007/s00438-020-01647-z 32048005

[117] Nazarian A Arbeev KG Yashkin AP Kulminski AM. Genome-wide analysis of genetic predisposition to common polygenic cancers. J Appl Genet (2022) 63(2):315–25. doi: 10.1007/s13353-021-00679-4 PMC8983541 34981446

[118] Anstee QM Darlay R Cockell S Meroni M Govaere O Tiniakos D. Genome-wide association study of non-alcoholic fatty liver and steatohepatitis in a histologically characterised cohort(☆). J Hepatol (2020) 73(3):505–15. doi: 10.1016/j.jhep.2020.04.003 32298765

[119] Liu GM Zeng HD Zhang CY Xu JW. Key genes associated with diabetes mellitus and hepatocellular carcinoma. Pathol Res Pract (2019) 215(11):152510. doi: 10.1016/j.prp.2019.152510 31591054

[120] Si Y Liu X Cheng M Wang M Gong Q Yang Y. Growth differentiation factor 15 is induced by hepatitis C virus infection and regulates hepatocellular carcinoma-related genes. PloS One (2011) 6(5):e19967. doi: 10.1371/journal.pone.0019967 21625435 PMC3100307

[121] Emmerson PJ Duffin KL Chintharlapalli S Wu X. GDF15 and growth control. Front Physiol (2018) 9:1712. doi: 10.3389/fphys.2018.01712 30542297 PMC6277789

[122] Luan HH Wang A Hilliard BK Carvalho F Rosen CE Ahasic AM. GDF15 is an inflammation-induced central mediator of tissue tolerance. Cell (2019) 178(5):1231–1244 e1211. doi: 10.1016/j.cell.2019.07.033 31402172 PMC6863354

[123] de Jager SC Bermudez B Bot I Koenen RR Bot M Kavelaars A. Growth differentiation factor 15 deficiency protects against atherosclerosis by attenuating CCR2-mediated macrophage chemotaxis. J Exp Med (2011) 208(2):217–25. doi: 10.1084/jem.20100370 PMC3039852 21242297

[124] Xu W-D Huang Q Yang C Li R Huang A-F. GDF-15: A potential biomarker and therapeutic target in systemic lupus erythematosus. Front Immunol (2022) 13:926373. doi: 10.3389/fimmu.2022.926373 35911685 PMC9332889

[125] Prendergast GC Metz R Muller AJ Merlo LM Mandik-Nayak L. IDO2 in immunomodulation and autoimmune disease. Front Immunol (2014) 5:585. doi: 10.3389/fimmu.2014.00585 25477879 PMC4238401

[126] Li P Xu W Liu F Zhu H Zhang L Ding Z. The emerging roles of IDO2 in cancer and its potential as a therapeutic target. Biomed Pharmacother (2021) 137:111295. doi: 10.1016/j.biopha.2021.111295 33550042

[127] Cutler JA Rush AJ McMahon FJ Laje G. Common genetic variation in the indoleamine-2,3-dioxygenase genes and antidepressant treatment outcome in major depressive disorder. J Psychopharmacol (2012) 26(3):360–7. doi: 10.1177/0269881111434622 22282879

[128] Kasamatsu T Hashimoto N Sakaya N Awata-Shiraiwa M Ishihara R Murakami Y. IDO2 rs10109853 polymorphism affects the susceptibility to multiple myeloma. Clin Exp Med (2021) 21:323–9. doi: 10.1007/s10238-020-00681-w 33709342

[129] Guba SC Stella G Turka LA June CH Thompson CB Emerson SG. Regulation of interleukin 3 gene induction in normal human T cells. J Clin Invest (1989) 84(6):1701–6. doi: 10.1172/jci114352 PMC304045 2556442

[130] Hong L Tang Y Pan S Xu M Shi Y Gao S. Interleukin 3-induced GITR promotes the activation of human basophils. Cytokine (2020) 136:155268. doi: 10.1016/j.cyto.2020.155268 32889153

[131] Okayama Y Begishvili TB Church MK. Comparison of mechanisms of IL-3 induced histamine release and IL-3 priming effect on human basophils. Clin Exp Allergy (1993) 23(11):901–10. doi: 10.1111/j.1365-2222.1993.tb00274.x 10779277

[132] Bondurant KL Lundgreen A Herrick JS Kadlubar S Wolff RK Slattery ML. Interleukin genes and associations with colon and rectal cancer risk and overall survival. Int J Cancer (2013) 132(4):905–15. doi: 10.1002/ijc.27660 PMC3470814 22674296

[133] Williams A Greene N Kimbro K. Increased circulating cytokine levels in African American women with obesity and elevated HbA1c. Cytokine (2020) 128:154989. doi: 10.1016/j.cyto.2020.154989 32004791 PMC7058975

[134] Meyer CG Calixto Fernandes MH Intemann CD Kreuels B Kobbe R Kreuzberg C. IL3 variant on chromosomal region 5q31-33 and protection from recurrent malaria attacks. Hum Mol Genet (2011) 20(6):1173–81. doi: 10.1093/hmg/ddq562 21224257

[135] Huang S Chen Z Yu JF Young D Bashey A Ho AD. Correlation between IL-3 receptor expression and growth potential of human CD34+ Hematopoietic cells from different tissues. Stem Cells (Dayton Ohio) (1999) 17(5):265–72. doi: 10.1002/stem.170265 10527461

[136] Agger K Miyagi S Pedersen MT Kooistra SM Johansen JV Helin K. Jmjd2/Kdm4 demethylases are required for expression of Il3ra and survival of acute myeloid leukemia cells. Genes Dev (2016) 30(11):1278–88. doi: 10.1101/gad.280495.116 PMC4911927 27257215

[137] Yan J Hou Z Ren Y Hu J. EP300-ZNF384 rearrangement drive B-cell acute lymphoblastic leukemia development by activating IL3-IL3RA signaling. Blood (2022) 140(Supplement 1):11508–8. doi: 10.1182/blood-2022-168535

[138] Bagaria J Kim K-O Bagyinszky E An SSA Baek J-H. Discriminating potential genetic markers for complete response and non-complete response patients to neoadjuvant chemotherapy with locally advanced rectal cancer. Int J Environ Res Public Health (2022) 19(7):4008. doi: 10.3390/ijerph19074008 35409691 PMC8997875

[139] Carvalho LH Sano GI Hafalla JC Morrot A De Lafaille MAC Zavala F. IL-4-secreting CD4+ T cells are crucial to the development of CD8+ T-cell responses against malaria liver stages. Nat Med (2002) 8(2):166–70. doi: 10.1038/nm0202-166 11821901

[140] Burke ML Jones MK Gobert GN Li YS Ellis MK McManus DP. Immunopathogenesis of human schistosomiasis. Parasite Immunol (2009) 31(4):163–76. doi: 10.1111/j.1365-3024.2009.01098.x 19292768

[141] Harris NL. Recent advances in type-2-cell-mediated immunity: insights from helminth infection. Immunity (2017) 47(6):1024–36. doi: 10.1016/j.immuni.2017.11.015 29262347

[142] Kim KM Hussein UK Park SH Moon YJ Zhang Z Ahmed AG. Expression of IL4Rα and IL13Rα1 are associated with poor prognosis of soft-tissue sarcoma of the extremities, superficial trunk, and retroperitoneum. Diagn Pathol (2021) 16(1):1–12. doi: 10.1186/s13000-020-01066-z 33419470 PMC7796579

[143] Tsao CH Shiau MY Chuang PH Chang YH Hwang J. Interleukin-4 regulates lipid metabolism by inhibiting adipogenesis and promoting lipolysis. J Lipid Res (2014) 55(3):385–97. doi: 10.1194/jlr.M041392 PMC3934724 24347527

[144] Battle NC Choudhry S Tsai HJ Eng C Kumar G Beckman KB. Ethnicity-specific gene–gene interaction between IL-13 and IL-4Rα among African Americans with asthma. Am J Respir Crit Care Med (2007) 175(9):881–7. doi: 10.1164/rccm.200607-992OC PMC1899298 17303794

[145] Narożna B Hoffmann A Sobkowiak P Schoneich N Bręborowicz A Szczepankiewicz A. Polymorphisms in the interleukin 4, interleukin 4 receptor and interleukin 13 genes and allergic phenotype: A case control study. Adv Med Sci (2016) 61(1):40–5. doi: 10.1016/j.advms.2015.07.003 26426602

[146] Hildebrandt MA Komaki R Liao Z Gu J Chang JY Ye Y. Genetic variants in inflammation-related genes are associated with radiation-induced toxicity following treatment for non-small cell lung cancer. PloS One (2010) 5(8):e12402. doi: 10.1371/journal.pone.0012402 20811626 PMC2928273

[147] Erlich HA Lohman K Mack SJ Valdes AM Julier C Mirel D. Association analysis of SNPs in the IL4R locus with type I diabetes. Genes Immun (2009) 10 Suppl 1(Suppl 1):S33–41. doi: 10.1038/gene.2009.89 PMC2810494 19956098

[148] Maier LM Chapman J Howson JM Clayton DG Pask R Strachan DP. No evidence of association or interaction between the IL4RA, IL4, and IL13 genes in type 1 diabetes. Am J Hum Genet (2005) 76(3):517–21. doi: 10.1086/428387 PMC1196402 15660293

[149] Qu HQ Tessier MC Fréchette R Bacot F Polychronakos C. Lack of association of type 1 diabetes with the IL4R gene. Diabetologia (2006) 49(5):958–61. doi: 10.1007/s00125-006-0199-2 16538488

[150] Kang S Tanaka T Narazaki M Kishimoto T. Targeting interleukin-6 signaling in clinic. Immunity (2019) 50(4):1007–23. doi: 10.1016/j.immuni.2019.03.026 30995492

[151] McKay JD Hung RJ Han Y Zong X Carreras-Torres R Christiani DC. Large-scale association analysis identifies new lung cancer susceptibility loci and heterogeneity in genetic susceptibility across histological subtypes. Nat Genet (2017) 49(7):1126–32. doi: 10.1038/ng.3892 PMC5510465 28604730

[152] Lippitz BE Harris RA. Cytokine patterns in cancer patients: A review of the correlation between interleukin 6 and prognosis. Oncoimmunology (2016) 5(5):e1093722. doi: 10.1080/2162402X.2015.1093722 27467926 PMC4910721

[153] Cupido AJ Asselbergs FW Natarajan P CHARGE Inflammation Working Group Ridker PM Hovingh GK. Dissecting the IL-6 pathway in cardiometabolic disease: A Mendelian randomization study on both IL6 and IL6R. Br J Clin Pharmacol (2022) 88(6):2875–84. doi: 10.1111/bcp.15191 PMC9303316 34931349

[154] Liao X Yu L Liu X Han C Yu T Qin W. Genome-wide association pathway analysis to identify candidate single nucleotide polymorphisms and molecular pathways associated with TP53 expression status in HBV-related hepatocellular carcinoma. Cancer Manage Res (2018) 10:953. doi: 10.2147/CMAR.S163209 PMC5937480 29760565

[155] Lundtoft C Seyfarth J Jacobsen M. IL7RA genetic variants differentially affect IL-7Rα expression and alternative splicing: a role in autoimmune and infectious diseases? Genes Immun (2020) 21(2):83–90. doi: 10.1038/s41435-019-0091-y 31929513

[156] Vudattu NK Magalhaes I Schmidt M Seyfert-Margolis V Maeurer MJ. Reduced numbers of IL-7 receptor (CD127) expressing immune cells and IL-7-signaling defects in peripheral blood from patients with breast cancer. Int J Cancer (2007) 121(7):1512–9. doi: 10.1002/ijc.22854 17546596

[157] Hoffmann M Enczmann J Balz V Kummer S Reinauer C Döing C. Interleukin-7 and soluble Interleukin-7 receptor levels in type 1 diabetes–Impact of IL7RA polymorphisms, HLA risk genotypes and clinical features. Clin Immunol (2022) 235:108928. doi: 10.1016/j.clim.2022.108928 35063672

[158] Guad RM Taylor-Robinson AW Wu YS Gan SH Zaharan NL Basu RC. Clinical and genetic risk factors for new-onset diabetes mellitus after transplantation (NODAT) in major transplant centres in Malaysia. BMC Nephrol (2020) 21:1–8. doi: 10.1186/s12882-020-02052-9 PMC7487857 32894076

[159] Mahajan R El-Omar EM Lissowska J Grillo P Rabkin CS Baccarelli A. Genetic variants in T helper cell type 1, 2 and 3 pathways and gastric cancer risk in a Polish population. Japanese J Clin Oncol (2008) 38(9):626–33. doi: 10.1093/jjco/hyn075 PMC2528891 18687755

[160] Van Dyke AL Cote ML Wenzlaff AS Chen W Abrams J Land S. Cytokine and cytokine receptor single-nucleotide polymorphisms predict risk for non-small cell lung cancer among women. Cancer Epidemiol Biomarkers Prev (2009) 18(6):1829–40. doi: 10.1158/1055-9965.EPI-08-0962 PMC3771080 19505916

[161] Chen Y Zheng T Lan Q Foss F Kim C Chen X. Cytokine polymorphisms in Th1/Th2 pathway genes, body mass index, and risk of non-Hodgkin lymphoma. Blood J Am Soc Hematol (2011) 117(2):585–90. doi: 10.1182/blood-2010-07-295097 PMC3031482 20952689

[162] Guzmán-Fulgencio M Berenguer J Jiménez-Sousa MA Pineda-Tenor D Aldámiz-Echevarria T García-Broncano P. Association between IL7R polymorphisms and severe liver disease in HIV/HCV coinfected patients: a cross-sectional study. J Trans Med (2015) 13(1):1–9. doi: 10.1186/s12967-015-0577-y PMC4487067 26123260

[163] Boguslawska J Kedzierska H Poplawski P Rybicka B Tanski Z Piekielko-Witkowska A. Expression of genes involved in cellular adhesion and extracellular matrix remodeling correlates with poor survival of patients with renal cancer. J Urol (2016) 195(6):1892–902. doi: 10.1016/j.juro.2015.11.050 26631499

[164] Zhang J Wang H Yuan C Wu J Xu J Chen S. ITGAL as a prognostic biomarker correlated with immune infiltrates in gastric cancer. Front Cell Dev Biol (2022) 10:808212. doi: 10.3389/fcell.2022.808212 35399517 PMC8987306

[165] Zhou Y Shi W Zhao D Xiao S Wang K Wang J. Identification of immune-associated genes in diagnosing aortic valve calcification with metabolic syndrome by integrated bioinformatics analysis and machine learning. Front Immunol (2022) 13:937886. doi: 10.3389/fimmu.2022.937886 35865542 PMC9295723

[166] Pençe HH Alsaadoni H Çaykara B Ötünçtemur A Kuraş S Tokat B. Investigation of LFA-1 rs2230433 variation in renal cell carcinoma tissues. Haydarpasa Numune Training Res Hosp Med J (2019) 59(3):216–9. doi: 10.14744/hnhj.2019.63325

[167] Fu Z Jiao M Zhang M Xu F Yuan W Pang D. LFA-1 gene polymorphisms are associated with the sporadic infiltrative duct breast carcinoma in Chinese Han women of Heilongjiang Province. Breast Cancer Res Treat (2011) 127(1):265–71. doi: 10.1007/s10549-010-1203-6 20953905

[168] Weber C Fraemohs L Dejana E. The role of junctional adhesion molecules in vascular inflammation. Nat Rev Immunol (2007) 7(6):467–77. doi: 10.1038/nri2096 17525755

[169] Verdino P Witherden DA Havran WL Wilson IA. The molecular interaction of CAR and JAML recruits the central cell signal transducer PI3K. Science (2010) 329(5996):1210–4. doi: 10.1126/science.1187996 PMC2951132 20813955

[170] Fang L Yu W Yu G Zhong F Ye B. Junctional adhesion molecule-like protein (JAML) is correlated with prognosis and immune infiltrates in lung adenocarcinoma. Med Sci Monitor (2022) 28:e933503. doi: 10.12659/MSM.933503 PMC8772237 35034089

[171] Fu Y Sun Y Wang M Hou Y Huang W Zhou D. Elevation of JAML promotes diabetic kidney disease by modulating podocyte lipid metabolism. Cell Metab (2020) 32(6):1052–62. doi: 10.1016/j.cmet.2020.10.019 33186558

[172] Huang W Wang B-O Hou Y-F Fu Y Cui S-J Zhu J-H. JAML promotes acute kidney injury mainly through a macrophage-dependent mechanism. JCI Insight (2022) 7(14):e158571. doi: 10.1172/jci.insight.158571 35708906 PMC9431718

[173] Spitali P Zaharieva I Bohringer S Hiller M Chaouch A Roos A. TCTEX1D1 is a genetic modifier of disease progression in Duchenne muscular dystrophy. Eur J Hum Genet EJHG (2020) 28(6):815–25. doi: 10.1038/s41431-019-0563-6 PMC7253478 31896777

[174] Koerber-Rosso I Brandt S von Schnurbein J Fischer-Posovszky P Hoegel J Rabenstein H. A fresh look to the phenotype in mono-allelic likely pathogenic variants of the leptin and the leptin receptor gene. Mol Cell Pediatr (2021) 8(1):10. doi: 10.1186/s40348-021-00119-7 34448070 PMC8390564

[175] Loos RJF Yeo GSH. The genetics of obesity: from discovery to biology. Nat Rev Genet (2022) 23(2):120–33. doi: 10.1038/s41576-021-00414-z PMC8459824 34556834

[176] Yadav A Deo N. Influence of leptin on immunity. Curr Immunol Rev (2013) 9(1):23–30. doi: 10.2174/1573395511309010004

[177] Li Z Yuan W Ning S Li J Zhai W Zhang S. Role of leptin receptor (LEPR) gene polymorphisms and haplotypes in susceptibility to hepatocellular carcinoma in subjects with chronic hepatitis B virus infection. Mol Diagnosis Ther (2012) 16(6):383–8. doi: 10.1007/s40291-012-0008-1 23090836

[178] Lin J Xie Z Lan B Guo Z Tang WF Liu C. Investigation of Leptin and its receptor (LEPR) for single nucleotide polymorphisms in colorectal cancer: a case-control study involving 2,306 subjects. Am J Trans Res (2020) 12(7):3613–28. doi: 10.1200/JCO.2020.38.15_suppl.e16100 PMC7407677 32774722

[179] Saukko M Kesäniemi YA Ukkola O. Leptin receptor Lys109Arg and Gln223Arg polymorphisms are associated with early atherosclerosis. Metab Syndrome Related Disord (2010) 8(5):425–30. doi: 10.1089/met.2010.0004 20874424

[180] Furusawa T Naka I Yamauchi T Natsuhara K Kimura R Nakazawa M. The Q223R polymorphism in LEPR is associated with obesity in Pacific Islanders. Hum Genet (2010) 127(3):287–94. doi: 10.1007/s00439-009-0768-9 20183928

[181] Hirayasu K Ohashi J Tanaka H Kashiwase K Ogawa A Takanashi M. Evidence for natural selection on leukocyte immunoglobulin-like receptors for HLA class I in northeast asians. Am J Hum Genet (2008) 82(5):1075–83. doi: 10.1016/j.ajhg.2008.03.012 PMC2427302 18439545

[182] Jiao Y Wang L Gu X Tao S Tian L Na R. LILRA3 is associated with benign prostatic hyperplasia risk in a Chinese population. Int J Mol Sci (2013) 14(5):8832–40. doi: 10.3390/ijms14058832 PMC3676759 23615473

[183] Singaraja RR Tietjen I Hovingh GK Franchini PL Radomski C Wong K. Identification of four novel genes contributing to familial elevated plasma HDL cholesterol in humans. J Lipid Res (2014) 55(8):1693–701. doi: 10.1194/jlr.M048710 PMC4109763 24891332

[184] Ryu M Chen Y Qi J Liu J Fan Z Nam G. LILRA3 binds both classical and non-classical HLA class I molecules but with reduced affinities compared to LILRB1/LILRB2: structural evidence. PloS One (2011) 6(4):e19245. doi: 10.1371/journal.pone.0019245 21559424 PMC3084784

[185] Argyriou E Roussos P Nezos A Venetsanopoulou A Boki KA Tzioufas A. Thu0204 association of lilra3 gene with lymphomagenesis risk in young ss patients. Ann Rheum Dis (2019) 78, 380. doi: 10.1136/annrheumdis-2019-eular.6054 30254034

[186] Cox AJ Zhang P Evans TJ Scott RJ Cripps AW West NP. Gene expression profiles in whole blood and associations with metabolic dysregulation in obesity. Obes Res Clin Pract (2018) 12(2):204–13. doi: 10.1016/j.orcp.2017.07.001 28755841

[187] Siddiqui MK. Genetic factors in statin intolerance. University of Dundee (2016).

[188] Mukherjee T Hovingh ES Foerster EG Abdel-Nour M Philpott DJ Girardin SE. NOD1 and NOD2 in inflammation, immunity and disease. Arch Biochem Biophysics (2019) 670:69–81. doi: 10.1016/j.abb.2018.12.022 30578751

[189] Wang D. NOD1 and NOD2 are potential therapeutic targets for cancer immunotherapy. Comput Intell Neurosci (2022) 2022. doi: 10.1155/2022/2271788 PMC9576356 36262606

[190] Carlos D Pérez MM Leite JA Rocha FA Martins LM Pereira CA. NOD2 deficiency promotes intestinal CD4+ T lymphocyte imbalance, metainflammation, and aggravates type 2 diabetes in murine model. Front Immunol (2020) 11:1265. doi: 10.3389/fimmu.2020.01265 32774333 PMC7381387

[191] Cavallari JF Pokrajac NT Zlitni S Foley KP Henriksbo BD Schertzer JD. NOD2 in hepatocytes engages a liver-gut axis to protect against steatosis, fibrosis, and gut dysbiosis during fatty liver disease in mice. Am J Physiol-Endocrinol Metab (2020) 319(2):E305–14. doi: 10.1152/ajpendo.00181.2020 32516028

[192] Cerhan JR Wang S Maurer MJ Ansell SM Geyer SM Cozen W. Prognostic significance of host immune gene polymorphisms in follicular lymphoma survival. Blood (2007) 109(12):5439–46. doi: 10.1182/blood-2006-11-058040 PMC1890834 17327408

[193] Barber GN. STING: infection, inflammation and cancer. Nat Rev Immunol (2015) 15(12):760–70. doi: 10.1038/nri3921 PMC5004891 26603901

[194] Zhang X Bai XC Chen ZJ. Structures and mechanisms in the cGAS-STING innate immunity pathway. Immunity (2020) 53(1):43–53. doi: 10.1016/j.immuni.2020.05.013 32668227

[195] Corrales L McWhirter SM Dubensky TW Jr. Gajewski TF. The host STING pathway at the interface of cancer and immunity. J Clin Invest (2016) 126(7):2404–11. doi: 10.1172/JCI86892 PMC4922692 27367184

[196] Oduro PK Zheng X Wei J Yang Y Wang Y Zhang H. The cGAS-STING signaling in cardiovascular and metabolic diseases: Future novel target option for pharmacotherapy. Acta Pharm Sin B (2022) 12(1):50–75. doi: 10.1016/j.apsb.2021.05.011 35127372 PMC8799861

[197] Jin L Xu L Yang IV Davidson EJ Schwartz DA Wurfel MM. Identification and characterization of a loss-of-function human MPYS variant. Genes Immun (2011) 12(4):263–9. doi: 10.1038/gene.2010.75 PMC3107388 21248775

[198] Yi G Brendel VP Shu C Li P Palanathan S Cheng Kao C. Single nucleotide polymorphisms of human STING can affect innate immune response to cyclic dinucleotides. PloS One (2013) 8(10):e77846. doi: 10.1371/journal.pone.0077846 24204993 PMC3804601

[199] Kennedy RB Haralambieva IH Ovsyannikova IG Voigt EA Larrabee BR Schaid DJ. Polymorphisms in STING Affect Human Innate Immune Responses to Poxviruses. Front Immunol (2020) 11:567348. 33154747 10.3389/fimmu.2020.567348 PMC7591719

[200] Straub RH. TRPV1, TRPA1, and TRPM8 channels in inflammation, energy redirection, and water retention: role in chronic inflammatory diseases with an evolutionary perspective. J Mol Med (2014) 92:925–37. doi: 10.1007/s00109-014-1175-9 24871046

[201] Kozyreva TV Khramova GM. Effects of activation of skin ion channels TRPM8, TRPV1, and TRPA1 on the immune response. Comparison with effects of cold and heat exposure. J Thermal Biol (2020) 93:102729. doi: 10.1016/j.jtherbio.2020.102729 33077140

[202] Yee NS. Roles of TRPM8 ion channels in cancer: proliferation, survival, and invasion. Cancers (2015) 7(4):2134–46. doi: 10.3390/cancers7040882 PMC4695882 26512697

[203] Liu Z Wu H Wei Z Wang X Shen P Wang S. TRPM8: a potential target for cancer treatment. J Cancer Res Clin Oncol (2016) 142:1871–81. doi: 10.1007/s00432-015-2112-1 PMC11819235 26803314

[204] Sanders OD Rajagopal JA Rajagopal L. Menthol to induce non-shivering thermogenesis via TRPM8/PKA signaling for treatment of obesity. J Obes Metab Syndrome (2021) 30(1):4–11. doi: 10.7570/jomes20038 PMC8017329 33071240

[205] Kozyreva TV Kozaruk VP Meyta ES. Effect of the peripheral TRPM8 ion channel activation on the cardiovascular parameters. Int Arch Clin Pharmacol (2019) 5:1–7. doi: 10.23937/2572-3987.1510019

[206] Naumov DE Perelman JM Kolosov VP Potapova TA Maksimov VN Zhou X. Transient receptor potential melastatin 8 gene polymorphism is associated with cold-induced airway hyperresponsiveness in bronchial asthma. Respirology (2015) 20(8):1192–7. doi: 10.1111/resp.12605 26272603

[207] Potapova TA Babenko VN Kobzev VF Romashchenko AG Maksimov VN Voevoda MI. Associations of cold receptor TRPM8 gene single nucleotide polymorphism with blood lipids and anthropometric parameters in Russian population. Bull Exp Biol Med (2014) 157(6):757–61. doi: 10.1007/s10517-014-2660-4 25348565

[208] Mangi MH Newland AC. Interleukin-3: promises and perspectives. Hematol (Amsterdam Netherlands) (1998) 3(1):55–66. doi: 10.1080/10245332.1998.11752123 27416283

[209] Wu C-H Lee T-H Yang S-F Tsao H-M Chang Y-J Chou C-H. Interleukin-3 polymorphism is associated with miscarriage of fresh in vitro fertilization cycles. Int J Environ Res Public Health (2019) 16(6):995. doi: 10.3390/ijerph16060995 30893922 PMC6466610

[210] Stomski FC Sun Q Bagley CJ Woodcock J Goodall G Andrews RK. Human interleukin-3 (IL-3) induces disulfide-linked IL-3 receptor alpha- and beta-chain heterodimerization, which is required for receptor activation but not high-affinity binding. Mol Cell Biol (1996) 16(6):3035–46. doi: 10.1128/MCB.16.6.3035 PMC231298 8649415

[211] Heeb LEM Egholm C Boyman O. Evolution and function of interleukin-4 receptor signaling in adaptive immunity and neutrophils. Genes Immun (2020) 21(3):143–9. doi: 10.1038/s41435-020-0095-7 PMC7274943 32139893

[212] Damen M Stankiewicz TE Park SH Helsley RN Chan CC Moreno-Fernandez ME. Non-hematopoietic IL-4Ralpha expression contributes to fructose-driven obesity and metabolic sequelae. Int J Obes (Lond) (2021) 45(11):2377–87. doi: 10.1038/s41366-021-00902-6 PMC8528699 34302121

[213] Zhu N Gong Y Chen XD Zhang J Long F He J. Association between the polymorphisms of interleukin-4, the interleukin-4 receptor gene and asthma. Chin Med J (Engl) (2013) 126(15):2943–51. 23924473

[214] ElKassar N Gress RE. An overview of IL-7 biology and its use in immunotherapy. J Immunotoxicol (2010) 7(1):1–7. doi: 10.3109/15476910903453296 20017587 PMC2826542

[215] Carrette F Surh CD. IL-7 signaling and CD127 receptor regulation in the control of T cell homeostasis. Semin Immunol (2012) 24(3):209–17. doi: 10.1016/j.smim.2012.04.010 PMC3367861 22551764

[216] Nguyen V Mendelsohn A Larrick JW. Interleukin-7 and immunosenescence. J Immunol Res (2017) 2017:4807853. doi: 10.1155/2017/4807853 28484723 PMC5397725

[217] Bossennec M Di Roio A Caux C Ménétrier-Caux C. MDR1 in immunity: friend or foe? Oncoimmunology (2018) 7(12):e1499388. doi: 10.1080/2162402X.2018.1499388 30524890 PMC6279327

[218] Wang BL Zhai HY Chen BY Zhai SP Yang HY Chen XP. Clinical relationship between MDR1 gene and gallbladder cancer. Hepatobiliary Pancreat Dis Int (2004) 3(2):296–9. 15138130

[219] Lai J Yang S Lin Z Huang W Li X Li R. Update on chemoresistance mechanisms to first-line chemotherapy for gallbladder cancer and potential reversal strategies. Am J Clin Oncol (2023) 46(4):131. doi: 10.1097/COC.0000000000000989 36867653 PMC10030176

[220] Ichihara S Yamada Y Kato K Hibino T Yokoi K Matsuo H. Association of a polymorphism of ABCB1 with obesity in Japanese individuals. Genomics (2008) 91(6):512–6. doi: 10.1016/j.ygeno.2008.03.004 18442890

[221] Wu J Wang X Chen H Yang R Yu H Wu Y. Type 2 diabetes risk and lipid metabolism related to the pleiotropic effects of an ABCB1 variant: A chinese family-based cohort study. Metabolites (2022) 12(9):875. doi: 10.3390/metabo12090875 36144279 PMC9502507

[222] Yan RJ Lou TT Wu YF Chen WS. Single nucleotide polymorphisms of ABCB1 gene and response to etanercept treatment in patients with ankylosing spondylitis in a Chinese Han population. Medicine (2017) 96(5):e5929. doi: 10.1097/MD.0000000000005929 28151874 PMC5293437

[223] Gervasini G Carrillo JA Garcia M San Jose C Cabanillas A Benitez J. Adenosine triphosphate-binding cassette B1 (ABCB1) (multidrug resistance 1) G2677T/A gene polymorphism is associated with high risk of lung cancer. Cancer (2006) 107(12):2850–7. doi: 10.1002/CNCR.22332 17120199

[224] Song F Zhang Y Pan Z Hu X Zhang Q Huang F. The role of alcohol dehydrogenase 1C in regulating inflammatory responses in ulcerative colitis. Biochem Pharmacol (2021) 192:114691. doi: 10.1016/j.bcp.2021.114691 34293286

[225] Chen Q Li F Gao Y Xu G Liang L Xu J. Identification of energy metabolism genes for the prediction of survival in hepatocellular carcinoma. Front Oncol (2020) 10:1210. doi: 10.3389/fonc.2020.01210 32903581 PMC7438573

[226] Liu X Li T Kong D You H Kong F Tang R. Prognostic implications of alcohol dehydrogenases in hepatocellular carcinoma. BMC Cancer (2020) 20(1):1204. doi: 10.1186/s12885-020-07689-1 33287761 PMC7720489

[227] Molinaro A Wahlström A Marschall HU. Role of bile acids in metabolic control. Trends Endocrinol Metab (2018) 29(1):31–41. doi: 10.1016/j.tem.2017.11.002 29195686

[228] McGlone ER Bloom SR. Bile acids and the metabolic syndrome. Ann Clin Biochem (2019) 56(3):326–37. doi: 10.1177/0004563218817798 30453753

[229] Li M Liu Z Song J Wang T Wang H Wang Y. Identification of down-regulated ADH1C is associated with poor prognosis in colorectal cancer using bioinformatics analysis. Front Mol Biosci (2022) 9:791249. doi: 10.3389/fmolb.2022.791249 35300114 PMC8921497

[230] Jaillon S Berthenet K Garlanda C. Sexual dimorphism in innate immunity. Clin Rev Allergy Immunol (2019) 56(3):308–21. doi: 10.1007/s12016-017-8648-x 28963611

[231] Wang P Zhang L Huang C Huang P Zhang J. Distinct prognostic values of alcohol dehydrogenase family members for non-small cell lung cancer. Med Sci Monit (2018) 24:3578–90. doi: 10.12659/MSM.910026 PMC6003262 29808834

[232] Shen XY Liu XP Song CK Wang YJ Li S Hu WD. Genome-wide analysis reveals alcohol dehydrogenase 1C and secreted phosphoprotein 1 for prognostic biomarkers in lung adenocarcinoma. J Cell Physiol (2019) 234(12):22311–20. doi: 10.1002/jcp.28797 31074035

[233] Xue Y Wang M Zhong D Tong N Chu H Sheng X. ADH1C Ile350Val polymorphism and cancer risk: evidence from 35 case–control studies. PloS One (2012) 7(5):e37227. doi: 10.1371/journal.pone.0037227 22675424 PMC3366713

[234] Oze I Matsuo K Suzuki T Kawase T Watanabe M Hiraki A. Impact of multiple alcohol dehydrogenase gene polymorphisms on risk of upper aerodigestive tract cancers in a Japanese population. Cancer Epidemiol Biomarkers Prev (2009) 18(11):3097–102. doi: 10.1158/1055-9965.Epi-09-0499 19861527

[235] Harada R Kimura M Sato Y Taniguchi T Tomonari T Tanaka T. APOB codon 4311 polymorphism is associated with hepatitis C virus infection through altered lipid metabolism. BMC Gastroenterol (2018) 18(1):24. doi: 10.1186/S12876-018-0747-5 29382324 PMC5791310

[236] Deng W Liu H Luo S Clarke J Glass C Su L. APOB genotypes and CDH13 haplotypes in the cholesterol-related pathway genes predict non-small cell lung cancer survival. Cancer Epidemiol Biomarkers Prev (2020) 29(6):1204–13. doi: 10.1158/1055-9965.EPI-19-1262 PMC7269811 32238407

[237] Jang SJ Tuan WL Hsu LA Er LK Teng MS Wu S. Pleiotropic effects of APOB variants on lipid profiles, metabolic syndrome, and the risk of diabetes mellitus. Int J Mol Sci (2022) 23(23):14963. doi: 10.3390/IJMS232314963/S1 36499290 PMC9735756

[238] Aceves-Ramírez M Valle Y Casillas-Muñoz F Martínez-Fernández DE Parra-Reyna B López-Moreno VA. Analysis of the APOB Gene and Apolipoprotein B Serum Levels in a Mexican Population with Acute Coronary Syndrome: Association with the Single Nucleotide Variants rs1469513, rs673548, rs676210, and rs1042034. Genet Res (Camb) (2022) 2022:4901090. doi: 10.1155/2022/4901090 35440891 PMC8991409

[239] Shishkova VN Adasheva TV Stakhovskaya LV Remennic AYU Valyaeva VV. Assessment of twenty-five variants of polymorphic genes for lipid and carbohydrate metabolism, vascular inflammation and the neurotransmitter system in the first non-cardioembolic ischemic stroke. Eur Heart J (2020) 41(Supplement_2):ehaa946.2408. doi: 10.1093/EHJCI/EHAA946.2408

[240] Yoshida T Kato K Yokoi K Watanabe S Metoki N Satoh K. Association of candidate gene polymorphisms with chronic kidney disease in Japanese individuals with hypertension. Hypertens Res (2009) 32(5):411–8. doi: 10.1038/hr.2009.22 19282863

[241] Walse B Dufe VT Svensson B Fritzson I Dahlberg L Khairoullina A. The structures of human dihydroorotate dehydrogenase with and without inhibitor reveal conformational flexibility in the inhibitor and substrate binding sites. Biochemistry (2008) 47(34):8929–36. doi: 10.1021/BI8003318 18672895

[242] Evers DL Wang X Huong SM Andreoni KA Huang ES. Inhibition of human cytomegalovirus signaling and replication by the immunosuppressant FK778. Antiviral Res (2005) 65(1):1–12. doi: 10.1016/j.antiviral.2004.03.007 15652966

[243] Luban J Sattler RA Mühlberger E Graci JD Cao L Weetall M. The DHODH inhibitor PTC299 arrests SARS-CoV-2 replication and suppresses induction of inflammatory cytokines. Virus Res (2021) 292:198246. doi: 10.1016/J.VIRUSRES.2020.198246 33249060 PMC7690341

[244] Mao C Liu X Zhang Y Lei G Yan Y Lee H. DHODH-mediated ferroptosis defence is a targetable vulnerability in cancer. Nature (2021) 593(7860):586–90. doi: 10.1038/S41586-021-03539-7 PMC8895686 33981038

[245] Zhou Y Tao L Zhou X Zuo Z Gong J Liu X. DHODH and cancer: promising prospects to be explored. Cancer Metab (2021) 9(1):22. doi: 10.1186/S40170-021-00250-Z 33971967 PMC8107416

[246] Zhang J Teran G Popa M Madapura H Ladds M Lianoudaki D. DHODH inhibition modulates glucose metabolism and circulating GDF15, and improves metabolic balance. iScience (2021) 24(5):102494. doi: 10.1016/j.isci.2021.102494 34113829 PMC8169992

[247] Liu K Jin X Zhang X Lian H Ye J. The mechanisms of nucleotide actions in insulin resistance. J Genet Genomics (2022) 49(4):299–307. doi: 10.1016/J.JGG.2022.01.006 35134563

[248] Pawlik A Herczynska M Kurzawski M Safranow K Dziedziejko V Drozdzik M. The effect of exon (19C>A) dihydroorotate dehydrogenase gene polymorphism on rheumatoid arthritis treatment with leflunomide. Pharmacogenomics (2009) 10(2):303–9. doi: 10.2217/14622416.10.2.303 19207032

[249] Soukup T Dosedel M Nekvindova J. Genetic polymorphisms in metabolic pathways of leflunomide in the treatment of rheumatoid arthritis. Clin Exp Rheumatol (2015) 33:426–32. 25664505

[250] Makarem YS Hareedy MS Hassanien M Ahmed EA Hetta HF Mohamed AAA. Frequency and impact of DHODH, ABCG2 and CYP2C19 SNPs on the therapeutic efficacy, tolerability and toxicity of leflunomide. Pharmacogenomics (2021) 22(18):1201–9. doi: 10.2217/PGS-2020-0146 34747629

[251] Wang H Li ZY Liu Y Persson J Beyer I Möller T. Desmoglein 2 is a receptor for adenovirus serotypes 3, 7, 11 and 14. Nat Med (2011) 17(1):96–104. doi: 10.1038/nm.2270 21151137 PMC3074512

[252] Wang J Hao S Gu J Rudd SG Wang Y. The prognostic and clinicopathological significance of desmoglein 2 in human cancers: a systematic review and meta-analysis. PeerJ (2022) 10:e13141. doi: 10.7717/peerj.13141 35345582 PMC8957267

[253] Myo Min KK Rojas-Canales D Penko D DeNichilo M Cockshell MP Ffrench CB. Desmoglein-2 is important for islet function and β-cell survival. Cell Death Dis (2022) 13(10):1–15. doi: 10.1038/s41419-022-05326-2 PMC9617887 36309486

[254] Choi H Koh HW Zhou L Cheng H Loh TP Parvaresh Rizi E. Plasma protein and microRNA biomarkers of insulin resistance: A network-based integrative-omics analysis. Front Physiol (2019) 10:379. doi: 10.3389/fphys.2019.00379 31024340 PMC6460474

[255] Liu K Wang YB Du JL Qu PF Ma L Tang X. Cardiac disease associated genetic variants in Yi nationality in regions with high incidence of Yunnan sudden unexplained death. Fa yi xue za zhi (2020) 36(4):497–501. doi: 10.12116/j.issn.1004-5619.2020.04.013 33047533

[256] Yamamura K Uruno T Shiraishi A Tanaka Y Ushijima M Nakahara T. The transcription factor EPAS1 links DOCK8 deficiency to atopic skin inflammation via IL-31 induction. Nat Commun (2017) 8(1):13946. doi: 10.1038/ncomms13946 28067314 PMC5228069

[257] Putra AC Eguchi H Lee KL Yamane Y Gustine E Isobe T. The A Allele at rs13419896 of EPAS1 is associated with enhanced expression and poor prognosis for non-small cell lung cancer. PloS One (2015) 10(8):e0134496. doi: 10.1371/journal.pone.0134496 26263511 PMC4532412

[258] Islam F Gopalan V Lu CT Pillai S Lam AK. Identification of novel mutations and functional impacts of EPAS1 in colorectal cancer. Cancer Med (2021) 10(16):5557–73. doi: 10.1002/cam4.4116 PMC8366083 34250767

[259] Gao F Yao Q Zhu J Chen W Feng X Feng B. Dyslipidemia and non-alcoholic fatty liver disease due to a somatic mutation in HIF2A. Lancet (2023). (pre-print). doi: 10.2139/ssrn.4401718

[260] Al-Khelaifi F Diboun I Donati F Botrè F Abraham D Hingorani A. Metabolic GWAS of elite athletes reveals novel genetically-influenced metabolites associated with athletic performance. Sci Rep (2019) 9(1):19889. doi: 10.1038/s41598-019-56496-7 31882771 PMC6934758

[261] Jiang LL Kobayashi A Matsuura H Fukushima H Hashimoto T. Purification and properties of human d-3-Hydroxyacyl-CoA dehydratase: Medium-chain enoyl-CoA hydratase is d-3-Hydroxyacyl-CoA Dehydratase1. J Biochem (1996) 120(3):624–32. doi: 10.1093/oxfordjournals.jbchem.a021458 8902629

[262] Rasiah KK Gardiner-Garden M Padilla EJ Möller G Kench JG Alles MC. HSD17B4 overexpression, an independent biomarker of poor patient outcome in prostate cancer. Mol Cell Endocrinol (2009) 301(1-2):89–96. doi: 10.1016/j.mce.2008.11.021 19100308

[263] Ferdinandusse S Denis S van Roermund CWT Wanders RJA Dacremont G. Identification of the peroxisomal β-oxidation enzymes involved in the degradation of long-chain dicarboxylic acids. J Lipid Res (2004) 45(6):1104–11. doi: 10.1194/jlr.M300512-JLR200 15060085

[264] Zhang X Yang H Zhang J Gao F Dai L. HSD17B4, ACAA1, and PXMP4 in peroxisome pathway are down-regulated and have clinical significance in non-small cell lung cancer. Front Genet (2020) 11:273. doi: 10.3389/fgene.2020.00273 32265992 PMC7103649

[265] Ferdinandusse S Houten SM. Peroxisomes and bile acid biosynthesis. Biochim Biophys Acta (BBA) - Mol Cell Res (2006) 1763(12):1427–40. doi: 10.1016/j.bbamcr.2006.09.001 17034878

[266] Cariaso M Lennon G. SNPedia: a wiki supporting personal genome annotation, interpretation and analysis. Nucleic Acids Res (2012) 40(Database issue):D1308–1312. doi: 10.1093/nar/gkr798 PMC3245045 22140107

[267] Karageorgi S McGrath M Lee IM Buring J Kraft P De Vivo I. Polymorphisms in genes hydroxysteroid-dehydrogenase-17b type 2 and type 4 and endometrial cancer risk. Gynecologic Oncol (2011) 121(1):54–8. doi: 10.1016/j.ygyno.2010.11.014 PMC3062639 21129770

[268] Ferlin A Ganz F Pengo M Selice R Frigo AC Foresta C. Association of testicular germ cell tumor with polymorphisms in estrogen receptor and steroid metabolism genes. Endocrine-Related Cancer (2010) 17(1):17. doi: 10.1677/ERC-09-0176 19776291

[269] Cui N Hu M Khalil RA. Chapter one - biochemical and biological attributes of matrix metalloproteinases. In: Khalil RA, editor. Progress in Molecular Biology and Translational Science, vol. 147. Cambridge, MA: Academic Press (2017). p. 1–73. 10.1016/bs.pmbts.2017.02.005 PMC5430303 28413025

[270] Shibuya M. VEGF-VEGFR system as a target for suppressing inflammation and other diseases. Endocr Metab Immune Disord-Drug Targets (2015) 15(2):135–44. doi: 10.2174/1871530315666150316121956 25772179

[271] Adamowicz M Radlwimmer B Rieker RJ Mertens D Schwarzbach M Schraml P. Frequent amplifications and abundant expression of TRIO, NKD2, and IRX2 in soft tissue sarcomas. Genes Chromosomes Cancer (2006) 45(9):829–38. doi: 10.1002/gcc.20343 16752383

[272] Liu T Zhou W Zhang F Shi G Teng H Xiao J. Knockdown of IRX2 inhibits osteosarcoma cell proliferation and invasion by the AKT/MMP9 signaling pathway. Mol Med Rep (2014) 10(1):169–74. doi: 10.3892/mmr.2014.2215 24806332

[273] Chen B Zhang Y Chen S Xuran L Dong J Chen W. The role of vascular endothelial growth factor in ischemic stroke. Pharmazie (2021) 76(4):127–31. doi: 10.1691/ph.2021.1315 33849695

[274] Si J Si Y Zhang B Lan G Wei J Huang B. Up-regulation of the IRX2 gene predicts poor prognosis in nasopharyngeal carcinoma. Int J Clin Exp Pathol (2018) 11(8):4073. 31949798 PMC6962795

[275] Gholipoorfeshkecheh R Agarwala S Krishnappa S Savitha MR Doddaiah N Ramachandra NB. Nuclear co-repressor 1: a potential candidate gene in the manifestation of congenital heart diseases. Int J Hum Genet (2020) 20(2):55–65. doi: 10.31901/24566330.2020/20.02.73

[276] Wu X Tsai CY Patam MB Zan H Chen JP Lipkin SM. A role for the MutL mismatch repair Mlh3 protein in immunoglobulin class switch DNA recombination and somatic hypermutation. J Immunol (2006) 176(9):5426–37. doi: 10.4049/JIMMUNOL.176.9.5426 PMC4621967 16622010

[277] Wu Y Berends MJ Sijmons RH Mensink RG Verlind E Kooi KA. A role for MLH3 in hereditary nonpolyposis colorectal cancer. Nat Genet (2001) 29(2):137–8. doi: 10.1038/ng1001-137 11586295

[278] Jing W Li L Zhang X Wu S Zhao J Hou Q. Genetic profiling of breast cancer with and without preexisting metabolic disease. Trans Oncol (2020) 13(2):245–53. doi: 10.1016/J.TRANON.2019.09.008 PMC6931193 31869749

[279] de Jong MM Hofstra RM Kooi KA Westra JL Berends MJ Wu Y. No association between two MLH3 variants (S845G and P844L) and colorectal cancer risk. Cancer Genet Cytogenetics (2004) 152(1):70–1. doi: 10.1016/j.cancergencyto.2003.10.008 15193445

[280] Ye F Cheng Q Shen J Zhou C Chen H. Mismatch repair gene MLH3 Pro844Leu and Thr942Ile polymorphisms and the susceptibility to cervical carcinoma and HPV infection: a case-control study in a Chinese population. PloS One (2014) 9(4):e96224. doi: 10.1371/journal.pone.0096224 24759751 PMC3997526

[281] Liu Y Zhang X Jia J Tang L Gao X Yan L. Correlation between polymorphisms in DNA mismatch repair genes and the risk of primary hepatocellular carcinoma for the Han population in northern China. Scand J Gastroenterol (2015) 50(11):1404–10. doi: 10.3109/00365521.2015.1045429 26027715

[282] Yang G Huang H Tang M Cai Z Huang C Qi B. Role of neuromedin B and its receptor in the innate immune responses against influenza A virus infection in *vitro* and in *vivo* . Vet Res (2019) 50(1):80. doi: 10.1186/s13567-019-0695-2 31601264 PMC6785861

[283] Zeng R Xiong X. Effect of NMB-regulated ERK1/2 and p65 signaling pathway on proliferation and apoptosis of cervical cancer. Pathol-Res Pract (2022) 238:154104. doi: 10.1016/j.prp.2022.154104 36095918

[284] Kirac D Kasimay Cakir O Avcilar T Deyneli O Kurtel H Yazici D. Effects of MC4R, FTO, and NMB gene variants to obesity, physical activity, and eating behavior phenotypes. IUBMB Life (2016) 68(10):806–16. doi: 10.1002/iub.1558 27634552

[285] Sagiv Y Hudspeth K Mattner J Schrantz N Stern RK Zhou D. Cutting edge: impaired glycosphingolipid trafficking and NKT cell development in mice lacking Niemann-Pick type C1 protein. J Immunol (2006) 177(1):26–30. doi: 10.4049/jimmunol.177.1.26 16785493

[286] O’Neill KI Kuo LW Williams MM Lind H Crump LS Hammond NG. NPC1 confers metabolic flexibility in Triple Negative Breast Cancer. Cancers (2022) 14(14):3543. doi: 10.3390/cancers14143543 35884604 PMC9319388

[287] Lamri A Pigeyre M Garver WS Meyre D. The extending spectrum of NPC1-related human disorders: From Niemann–Pick C1 disease to obesity. Endocr Rev (2018) 39(2):192–220. doi: 10.1210/er.2017-00176 29325023 PMC5888214

[288] Chiorean A Garver WS Meyre D. Signatures of natural selection and ethnic-specific prevalence of NPC1 pathogenic mutations contributing to obesity and Niemann–Pick disease type C1. Sci Rep (2020) 10(1):1–9. doi: 10.1038/s41598-020-75919-4 33139814 PMC7608643

[289] Carette JE Raaben M Wong AC Herbert AS Obernosterer G Mulherkar N. Ebola virus entry requires the cholesterol transporter Niemann–Pick C1. Nature (2011) 477(7364):340–3. doi: 10.1038/nature10348 PMC3175325 21866103

[290] Al-Daghri NM Cagliani R Forni D Alokail MS Pozzoli U Alkharfy KM. Mammalian NPC1 genes may undergo positive selection and human polymorphisms associate with type 2 diabetes. BMC Med (2012) 10(1):1–11. doi: 10.1186/1741-7015-10-140 23153210 PMC3520752

[291] Meyre D Delplanque J Chèvre JC Lecoeur C Lobbens S Gallina S. Genome-wide association study for early-onset and morbid adult obesity identifies three new risk loci in European populations. Nat Genet (2009) 41(2):157–9. doi: 10.1038/ng.301 19151714

[292] Afzali M Hashemi M Tabatabaei SP Fakheri KT Nakhaee A. Association between the rs1805081 polymorphism of Niemann−Pick type C1 gene and cardiovascular disease in a sample of an Iranian population. Biomed Rep (2017) 6(1):83–8. doi: 10.3892/br.2016.802 PMC5244780 28123713

[293] Tavanti A Campa D Bertozzi A Pardini G Naglik JR Barale R. Candida albicans isolates with different genomic backgrounds display a differential response to macrophage infection. Microbes Infect (2006) 8(3):791–800. doi: 10.1016/j.micinf.2005.09.016 16473540

[294] Zhang C Mei W Zeng C. Oncogenic Neuregulin 1 gene (NRG1) fusions in cancer: A potential new therapeutic opportunities. Biochim Biophys Acta (BBA)-Reviews Cancer (2022) 1877(3):188707. doi: 10.1016/j.bbcan.2022.188707 35247506

[295] Gumà A Díaz-Sáez F Camps M Zorzano A. Neuregulin, an effector on mitochondria metabolism that preserves insulin sensitivity. Front Physiol (2020) 11:696. doi: 10.3389/fphys.2020.00696 32655416 PMC7324780

[296] Yang JZ Si TM Ruan Y Ling YS Han YH Wang XL. Association study of neuregulin 1 gene with schizophrenia. Mol Psychiatry (2003) 8(7):706–9. doi: 10.1038/sj.mp.4001377 12874607

[297] Andre K Kampman O Viikki M Setälä-Soikkeli E Illi A Mononen N. BDNF and NRG1 polymorphisms and temperament in selective serotonin reuptake inhibitor-treated patients with major depression. Acta Neuropsychiatr (2018) 30(3):168–74. doi: 10.1017/neu.2017.37 29310728

[298] Hsu CG Fazal F Rahman A Berk BC Yan C. Phosphodiesterase 10A is a key mediator of lung inflammation. J Immunol (2021) 206(12):3010–20. 10.4049/jimmunol.2001026 PMC8664899 34117108

[299] Cai Y Cai Y Yan C Guzman RJ. Phosphodiesterase 10A regulates vascular inflammation and pathogenesis of abdominal aortic aneurysms. Circulation (2015) 132(Suppl 3):A18182–A.

[300] Borneman RM Gavin E Musiyenko A Richter W Lee KJ Crossman DK. Phosphodiesterase 10A (PDE10A) as a novel target to suppress β-catenin and RAS signaling in epithelial ovarian cancer. J Ovarian Res (2022) 15(1):120. 36324187 10.1186/s13048-022-01050-9 PMC9632086

[301] Kilanowska A Ziolkowska A. Role of phosphodiesterase in the biology and pathology of diabetes. Int J Mol Sci (2020) 21(21). doi: 10.3390/ijms21218244 PMC7662747 33153226

[302] Hu Y Deng L Zhang J Fang X Mei P Cao X. A pooling genome-wide association study combining a pathway analysis for typical sporadic Parkinson’s disease in the Han population of Chinese mainland. Mol Neurobiol (2016) 53:4302–18. doi: 10.1007/s12035-015-9331-y 26227905

[303] Pirola CJ Salatino A Sookoian S. Pleiotropy within gene variants associated with nonalcoholic fatty liver disease and traits of the hematopoietic system. World J Gastroenterol (2021) 27(4):305–20. doi: 10.3748/wjg.v27.i4.305 PMC7852588 33584064

[304] Trépo E Nahon P Bontempi G Valenti L Falleti E Nischalke HD. Association between the PNPLA3 (rs738409 C>G) variant and hepatocellular carcinoma: Evidence from a meta-analysis of individual participant data. Hepatology (2014) 59(6):2170–7. doi: 10.1002/hep.26767 24114809

[305] Bing H Wang W Li YL. [Correlation between PNPLA3 rs738409 and TM6SF2 rs58542926 gene polymorphism and primary liver cancer in the Han Population of China's Northeast region]. Zhonghua gan zang bing za zhi = Zhonghua ganzangbing zazhi = Chin J Hepatol (2021) 29(2):156–62. doi: 10.3760/cma.j.cn501113-20191021-00390 PMC12814721 33685085

[306] Zhang H Liu J Yang Z Zeng L Wei K Zhu L. TCR activation directly stimulates PYGB-dependent glycogenolysis to fuel the early recall response in CD8(+) memory T cells. Mol Cell (2022) 82(16):3077–3088 e3076. doi: 10.1016/j.molcel.2022.06.002 35738262

[307] Wang Z Han G Liu Q Zhang W Wang J. Silencing of PYGB suppresses growth and promotes the apoptosis of prostate cancer cells via the NF−κB/Nrf2 signaling pathway. Mol Med Rep (2018) 18(4):3800–8. doi: 10.3892/mmr.2018.9388 PMC6131497 30106110

[308] Xia B Zhang K Liu C. PYGB promoted tumor progression by regulating Wnt/β-catenin pathway in gastric cancer. Technol Cancer Res Treat (2020) 19:1533033820926592. doi: 10.1177/1533033820926592 32462986 PMC7257874

[309] Xiao L Wang W Huangfu Q Tao H Zhang J. PYGB facilitates cell proliferation and invasiveness in non-small cell lung cancer by activating the Wnt–β-catenin signaling pathway. Biochem Cell Biol (2020) 98(5):565–74. doi: 10.1139/bcb-2019-0445 32191839

[310] Newgard CB Littman DR van Genderen C Smith M Fletterick RJ. Human brain glycogen phosphorylase. Cloning, sequence analysis, chromosomal mapping, tissue expression, and comparison with the human liver and muscle isozymes. J Biol Chem (1988) 263(8):3850–7. 3346228

[311] Walton SJ. Clinical and cellular studies in the pathogenesis of desmoid tumours in familial adenomatous polyposis (FAP). Department of Surgery & Cancer, Imperial College, London (2016).

[312] Greulich W Wagner M Gaidt MM Stafford C Cheng Y Linder A. TLR8 is a sensor of RNase T2 degradation products. Cell (2019) 179(6):1264–1275 e1213. doi: 10.1016/j.cell.2019.11.001 31778653 PMC7116005

[313] Acquati F Mortara L De Vito A Baci D Albini A Cippitelli M. Innate immune response regulation by the human RNASET2 tumor suppressor gene. Front Immunol (2019) 10:2587. doi: 10.3389/fimmu.2019.02587 31749812 PMC6848152

[314] Sletten AC Peterson LR Schaffer JE. Manifestations and mechanisms of myocardial lipotoxicity in obesity. J Intern Med (2018) 284(5):478–91. doi: 10.1111/joim.12728 PMC6045461 29331057

[315] Zhang H Baldwin DA Bukowski RK Parry S Xu Y Song C. A genome-wide association study of early spontaneous preterm delivery. Genet Epidemiol (2015) 39(3):217–26. doi: 10.1002/gepi.21887 PMC4366311 25599974

[316] Sabui S Skupsky J Kapadia R Cogburn K Lambrecht NW Agrawal A. Tamoxifen-induced, intestinal-specific deletion of Slc5a6 in adult mice leads to spontaneous inflammation: involvement of NF-κB, NLRP3, and gut microbiota. Am J Physiol-Gastrointestinal Liver Physiol (2019) 317(4):G518–30. doi: 10.1152/ajpgi.00172.2019 PMC6842991 31369292

[317] Sun T Wang D Ping Y Sang Y Dai Y Wang Y. Integrated profiling identifies SLC5A6 and MFAP2 as novel diagnostic and prognostic biomarkers in gastric cancer patients. Int J Oncol (2020) 56(2):460–9. doi: 10.3892/ijo.2019.4944 PMC6959404 31894266

[318] Hsieh CH Chen PC Shieh CC. Novel SLC5A6 mutations lead to B lymphocyte maturation defects with metabolic abnormality rescuable by biotin replenishment. J Immunol (2022) 208(1_Supplement):168–03. doi: 10.4049/jimmunol.208.Supp.168.03 38036278

[319] Shin SY Fauman EB Petersen AK Krumsiek J Santos R Huang J. An atlas of genetic influences on human blood metabolites. Nat Genet (2014) 46(6):543–50. doi: 10.1038/ng.2982 PMC4064254 24816252

[320] Shu X Wu L Khankari NK Shu XO Wang TJ Michailidou K. Associations of obesity and circulating insulin and glucose with breast cancer risk: a Mendelian randomization analysis. Int J Epidemiol (2019) 48(3):795–806. doi: 10.1093/ije/dyy201 30277539 PMC6734940

[321] Zhang J Ali AM Lieu YK Liu Z Gao J Rabadan R. Disease-causing mutations in SF3B1 alter splicing by disrupting interaction with SUGP1. Mol Cell (2019) 76(1):82–95. doi: 10.1016/j.molcel.2019.07.017 31474574 PMC7065273

[322] Liu Z Zhang J Sun Y Perea-Chamblee TE Manley JL Rabadan R. Pan-cancer analysis identifies mutations in SUGP1 that recapitulate mutant SF3B1 splicing dysregulation. Proc Natl Acad Sci (2020) 117(19):10305–12. doi: 10.1073/pnas.1922622117 PMC7229667 32332164

[323] Strachan DP Rudnicka AR Power C Shepherd P Fuller E Davis A. Lifecourse influences on health among british adults: effects of region of residence in childhood and adulthood. Int J Epidemiol. (2007) 36(3):522–31. doi: 10.1093/ije/dyl309 17255346

[324] Heid IM Jackson AU Randall JC Winkler TW Qi L Steinthorsdottir V. Meta-analysis identifies 13 new loci associated with waist-hip ratio and reveals sexual dimorphism in the genetic basis of fat distribution. Nat Genet (2010) 42(11):949–60. doi: 10.1038/ng.685 PMC3000924 20935629

[325] Dupuis J Langenberg C Prokopenko I Saxena R Soranzo N Jackson AU. New genetic loci implicated in fasting glucose homeostasis and their impact on type 2 diabetes risk. Nat Genet (2010) 42(2):105–16. doi: 10.1038/ng.520 PMC3018764 20081858

[326] van Helvoort A Smith AJ Sprong H Fritzsche I Schinkel AH Borst P. MDR1 p-glycoprotein is a lipid translocase of broad specificity, while MDR3 p-glycoprotein specifically translocates phosphatidylcholine. Cell (1996) 87(3):507–17. 10.1016/s0092-8674(00)81370-7 8898203

[327] Edelmann L Edelmann W. Loss of DNA mismatch repair function and cancer predisposition in the mouse: animal models for human hereditary nonpolyposis colorectal cancer. Am J Med Genet Part C Semin Med Genet (2004) 129C(1):91–9. doi: 10.1002/AJMG.C.30021 15264277

[328] Khailany RA Ozaslan M. Exonic sequencing and MLH3 gene expression analysis of breast cancer patients. Cell Mol Biol (Noisy-Le-Grand France) (2021) 67(3):35–43. doi: 10.14715/CMB/2021.67.3.5 34933735

[329] Devaraj S Semaan JR Jialal I. Biochemistry, Apolipoprotein B. In: StatPearls. StatPearls Publishing (Treasure Island, FL) (2023). Available at: https://www.ncbi.nlm.nih.gov/books/NBK538139/. 30844166

[330] Fang J Uchiumi T Yagi M Matsumoto S Amamoto R Takazaki S. Dihydro-orotate dehydrogenase is physically associated with the respiratory complex and its loss leads to mitochondrial dysfunction. Biosci Rep (2013) 33(2):217–27. 10.1042/BSR20120097 PMC3564035 23216091

[331] Huang FC. The role of sphingolipids on innate immunity to intestinal salmonella infection. Int J Mol Sci (2017) 18(8):1720. doi: 10.3390/ijms18081720 28783107 PMC5578110

[332] Fusco JP Pita G Pajares MJ Andueza MP Patino-Garcia A de-Torres JP. Genomic characterization of individuals presenting extreme phenotypes of high and low risk to develop tobacco-induced lung cancer. Cancer Med (2018) 7(7):3474–83. doi: 10.1002/cam4.1500 PMC6051154 29766673

[333] Hicks AA Pramstaller PP Johansson A Vitart V Rudan I Ugocsai P. Genetic determinants of circulating sphingolipid concentrations in European populations. PloS Genet (2009) 5(10):e1000672. doi: 10.1371/journal.pgen.1000672 19798445 PMC2745562

[334] Shen ZJ Hu J Esnault S Dozmorov I Malter JS. RNA Seq profiling reveals a novel expression pattern of TGF-beta target genes in human blood eosinophils. Immunol Lett (2015) 167(1):1–10. doi: 10.1016/j.imlet.2015.06.012 26112417 PMC6208316

[335] Zhang N Di J Wang Z Gao P Jiang B Su X. Genomic profiling of colorectal cancer with isolated lung metastasis. Cancer Cell Int (2020) 20:281. doi: 10.1186/s12935-020-01373-x 32624706 PMC7329491

[336] Ganev M Balabanski L Serbezov D Karachanak-Yankova S Vazharova R Nesheva D. Prioritization of genetic variants predisposing to coronary heart disease in the bulgarian population using centenarian exomes. Biotechnol Biotechnol Equipment. (2019) 33(1):1757–65. doi: 10.1080/13102818.2019.1700164

[337] Al Gadban MM German J Truman JP Soodavar F Riemer EC Twal WO. Lack of nitric oxide synthases increases lipoprotein immune complex deposition in the aorta and elevates plasma sphingolipid levels in lupus. Cell Immunol (2012) 276(1-2):42–51. doi: 10.1016/j.cellimm.2012.03.007 22560558 PMC3399025

[338] Miura K Nagahashi M Prasoon P Hirose Y Kobayashi T Sakata J. Dysregulation of sphingolipid metabolic enzymes leads to high levels of sphingosine-1-phosphate and ceramide in human hepatocellular carcinoma. Hepatol Res (2021) 51(5):614–26. doi: 10.1111/hepr.13625 33586816

[339] Fernández M Alarcón GS Calvo-alén J Andrade R McGwin G Jr. Vilá LM. A multiethnic, multicenter cohort of patients with systemic lupus erythematosus (SLE) as a model for the study of ethnic disparities in SLE. Arthritis Care Res (2007) 57(4):576–84. doi: 10.1002/art.22672 17471524

[340] Takeda H Takai A Inuzuka T Marusawa H. Genetic basis of hepatitis virus-associated hepatocellular carcinoma: linkage between infection, inflammation, and tumorigenesis. J Gastroenterol (2017) 52(1):26–38. doi: 10.1007/s00535-016-1273-2 27714455

[341] Elmessaoudi-Idrissi M Windisch MP Kettani A Altawalah H Pineau P Benjelloun S. An integrative gene expression microarray meta-analysis identifies host factors and key signatures involved in hepatitis B virus infection. Infect Disord Drug Targets (2020) 20(5):698–707. doi: 10.2174/1871526519666190807153901 31389317

[342] Wolff RK Hoffman MD Wolff EC Herrick JS Sakoda LC Samowitz WS. Mutation analysis of adenomas and carcinomas of the colon: Early and late drivers. Genes Chromosomes Cancer (2018) 57(7):366–76. doi: 10.1002/gcc.22539 PMC5951744 29575536

[343] Liu Y Xia J McKay J Tsavachidis S Xiao X Spitz MR. Rare deleterious germline variants and risk of lung cancer. NPJ Precis Oncol (2021) 5(1):12. doi: 10.1038/s41698-021-00146-7 33594163 PMC7887261

[344] The UniProt Consortium. UniProt: the universal protein knowledgebase in 2023. Nucleic Acids Res (2023) 51(D1):D523–31. doi: 10.1093/nar/gkac1052 PMC9825514 36408920

[345] Lu W Liu CC Thottassery JV Bu G Li Y. Mesd is a universal inhibitor of Wnt coreceptors LRP5 and LRP6 and blocks Wnt/beta-catenin signaling in cancer cells. Biochemistry (2010) 49(22):4635–43. doi: 10.1021/bi1001486 PMC2882879 20446724

[346] Gay A Towler DA. Wnt signaling in cardiovascular disease: opportunities and challenges. Curr Opin Lipidol (2017) 28(5):387–96. doi: 10.1097/MOL.0000000000000445 PMC5773247 28723729

[347] Luck K Kim D-K Lambourne L Spirohn K Begg BE Bian W. A reference map of the human binary protein interactome. Nature (2020) 580(7803):402–8. doi: 10.1038/s41586-020-2188-x PMC7169983 32296183

[348] Xiao C Yang L Jin L Zhang F Liu J Yu C. MAGEA11 as a STAD prognostic biomarker associated with immune infiltration. Diagnostics (2022) 12(10):2506. doi: 10.3390/diagnostics12102506 36292195 PMC9600629

[349] Gonzalez-Pons M Torres-Cintrón CR Soto-Salgado M Vargas-Ramos Y Perez-Portocarrero L Morgan DR. Racial/ethnic disparities in gastric cancer: A 15-year population-based analysis. Cancer Med (2023) 12(2):1860–8. doi: 10.1002/cam4.4997 PMC9883558 35785449

[350] Tang Q Liu Q Li Y Mo L He J. CRELD2, endoplasmic reticulum stress, and human diseases. Front Endocrinol (Lausanne) (2023) 14:1117414. doi: 10.3389/fendo.2023.1117414 36936176 PMC10018036

[351] Klein RJ Zeiss C Chew EY Tsai JY Sackler RS Haynes C. Complement factor h polymorphism in age-related macular degeneration. Science (2005) 308(5720):385–9. doi: 10.1126/science.1109557 PMC1512523 15761122

[352] Jatoi I Sung H Jemal A. The emergence of the racial disparity in U.S. Breast-cancer mortality. New Engl J Med (2022) 386(25):2349–52. doi: 10.1056/NEJMp2200244 35713541

[353] Karakas C Wang C Deng F Huang H Wang D Lee P. Molecular mechanisms involving prostate cancer racial disparity. Am J Clin Exp Urol (2017) 5(3):34–48. 29181436 PMC5698597

[354] Dougan M Dranoff G Dougan SK. GM-CSF, IL-3, and IL-5 family of cytokines: regulators of inflammation. Immunity (2019) 50(4):796–811. doi: 10.1016/j.immuni.2019.03.022 30995500 PMC12512237

[355] Watanabe-Smith K Tognon C Tyner JW Meijerink JP Druker BJ Agarwal A. Discovery and functional characterization of a germline, CSF2RB-activating mutation in leukemia. Leukemia (2016) 30(9):1950–3. doi: 10.1038/leu.2016.95 PMC5014660 27118405

[356] Rashid M Ali R Almuzzaini B Song H AlHallaj A Abdulkarim AA. Discovery of a novel potentially transforming somatic mutation in CSF2RB gene in breast cancer. Cancer Med (2021) 10(22):8138–50. doi: 10.1002/cam4.4106 PMC8607246 34729943

[357] Puchenkova OA Nadezhdin SV Soldatov VO Zhuchenko MA Korshunova DS Kubekina MV. Study of antiatherosclerotic and endothelioprotective activity of peptide agonists of EPOR/CD131 heteroreceptor. Pharm Pharmacol (2020) 8(2):100–11. doi: 10.19163/2307-9266-2020-8-2-100-111

[358] Kim HY Hong S. Multi-faceted roles of DNAJB protein in cancer metastasis and clinical implications. Int J Mol Sci (2022) 23(23):14970. doi: 10.3390/ijms232314970 36499297 PMC9737691

[359] Plenge RM Seielstad M Padyukov L Lee AT Remmers EF Ding B. TRAF1-C5 as a risk locus for rheumatoid arthritis–a genomewide study. N Engl J Med (2007) 357(12):1199–209. doi: 10.1056/NEJMoa073491 PMC2636867 17804836

[360] Genest J Schwertani A Choi HY. Membrane microdomains and the regulation of HDL biogenesis. Curr Opin Lipidol (2018) 29(1):36–41. doi: 10.1097/MOL.0000000000000470 29135688

[361] Zhang Z Zhu H Hu J. CircRAB11FIP1 promoted autophagy flux of ovarian cancer through DSC1 and miR-129. Cell Death Dis (2021) 12(2):1–12. doi: 10.1038/s41419-021-03486-1 33637694 PMC7910449

[362] Wang Y Chen C Wang X Jin F Liu Y Liu H. Lower DSC1 expression is related to the poor differentiation and prognosis of head and neck squamous cell carcinoma (HNSCC). J Cancer Res Clin Oncol (2016) 142(12):2461–8. doi: 10.1007/s00432-016-2233-1 PMC11819397 27601166

[363] Myklebust MP Fluge Ø Immervoll H Skarstein A Balteskard L Bruland O. Expression of DSG1 and DSC1 are prognostic markers in anal carcinoma patients. Br J Cancer (2012) 106(4):756–62. doi: 10.1038/bjc.2011.548 PMC3322941 22333708

[364] Mei S Chelmow D Gecsi K Barkley J Barrows E Brooks R. Health disparities in ovarian cancer: report from the ovarian cancer evidence review conference. Obstet Gynecol (2023) 142(1):196–210. doi: 10.1097/AOG.0000000000005210 37348095 PMC10278570

[365] Hutchinson MA Park HS Zanotti KJ Alvarez-Gonzalez J Zhang J Zhang L. Auto-antibody production during experimental atherosclerosis in apoE(-/-) mice. Front Immunol (2021) 12:695220. doi: 10.3389/fimmu.2021.695220 34305930 PMC8299997

[366] Naderi A. Genomic and epigenetic aberrations of chromosome 1p36. 13 have prognostic implications in Malignancies. Chromosome Res (2020) 28(3):307–30. doi: 10.1007/s10577-020-09638-x 32816122

[367] Ma Y Kan C Qiu H Liu Y Hou N Han F. Transcriptomic analysis reveals the protective effects of empagliflozin on lipid metabolism in nonalcoholic fatty liver disease. Front Pharmacol (2021) 12. doi: 10.3389/fphar.2021.793586 PMC8724565 34992540

[368] Ruggiero AD Vemuri R Block M DeStephanis D Davis M Chou J. Macrophage phenotypes and gene expression patterns are unique in naturally occurring metabolically healthy obesity. Int J Mol Sci (2022) 23(20):12680. doi: 10.3390/ijms232012680 36293536 PMC9604193

[369] Rolland T Taşan M Charloteaux B Pevzner SJ Zhong Q Sahni N. A proteome-scale map of the human interactome network. Cell (2014) 159(5):1212–26. doi: 10.1016/j.cell.2014.10.050 PMC4266588 25416956

[370] He C Liu W Xiong Y Wang Y Pan L Luo L. VSNL1 promotes cell proliferation, migration, and invasion in colorectal cancer by binding with COL10A1. Ann Clin Lab Sci (2022) 52(1):60–72. 35181619

[371] Aiba T Hijiya N Akagi T Tsukamoto Y Hirashita Y Kinoshita K. Overexpression of VSNL1 enhances cell proliferation in colorectal carcinogenesis. Pathobiology (2023) 1–11. doi: 10.1159/000533877 37797604

[372] Dai QQ Wang YY Jiang YP Li L Wang HJ. VSNL1 promotes gastric cancer cell proliferation and migration by regulating P2X3/P2Y2 receptors and is a clinical indicator of poor prognosis in gastric cancer patients. Gastroenterol Res Pract (2020) 2020:7241942. doi: 10.1155/2020/7241942 33376484 PMC7744243

[373] Bui A Yang L Soroudi C May FP. Racial and ethnic disparities in incidence and mortality for the five most common gastrointestinal cancers in the United States. J Natl Med Assoc (2022) 114(4):426–9. doi: 10.1016/j.jnma.2022.04.001 35525822

[374] Liu Y Lin S Xie Y Zhao L Du H Yang S. ILDR1 promotes influenza A virus replication through binding to PLSCR1. Sci Rep (2022) 12(1):1–13. doi: 10.1038/s41598-022-12598-3 35595813 PMC9122930

[375] Wang L Zhai R Song G Wang Y. Analyses of the expression and prognosis of ILDR1 in human gastric cancer. Heliyon (2022) 8(9):e10253. doi: 10.1016/j.heliyon.2022.e10253 36091962 PMC9450077

[376] Chandra R Aryal DK Douros JD Shahid R Davis SJ Campbell JE. Ildr1 gene deletion protects against diet-induced obesity and hyperglycemia. PloS One (2022) 17(6):e0270329. doi: 10.1371/journal.pone.0270329 35749484 PMC9231709

[377] Deng S Wang Y Brosseau JP Wang Y Xu K Chi H. The ITPRIPL1-CD3ϵ axis: a novel immune checkpoint controlling T cells activation. bioRxiv (2022). doi: 10.1101/2022.02.25.481189

[378] Wang SC Liao LM Ansar M Lin SY Hsu WW Su CM. Automatic detection of the circulating cell-free methylated DNA pattern of GCM2, ITPRIPL1 and CCDC181 for detection of early breast cancer and surgical treatment response. Cancers (2021) 13(6):1375. doi: 10.3390/cancers13061375 33803633 PMC8002961

[379] Joshi H Vastrad B Joshi N Vastrad C. Integrated bioinformatics analysis reveals novel key biomarkers in diabetic nephropathy. SAGE Open Med (2022) 10:1–32. doi: 10.1177/20503121221137005 PMC9661593 36385790

[380] Choi JH Zhong X Zhang Z Su L McAlpine W Misawa T. Essential cell-extrinsic requirement for PDIA6 in lymphoid and myeloid development. J Exp Med (2020) 217(4):e20190006. doi: 10.1084/jem.20190006 31985756 PMC7144532

[381] Jordan PA Stevens JM Hubbard GP Barrett NE Sage T Authi KS. A role for the thiol isomerase protein ERP5 in platelet function. Blood (2005) 105(4):1500–7. doi: 10.1182/blood-2004-02-0608 15466936

[382] Mao L Wu X Gong Z Yu M Huang Z. PDIA6 contributes to aerobic glycolysis and cancer progression in oral squamous cell carcinoma. World J Surg Oncol (2021) 19:1–9. doi: 10.1186/s12957-021-02190-w 33761940 PMC7992853

[383] Bai Y Liu X Qi X Liu X Peng F Li H. PDIA6 modulates apoptosis and autophagy of non-small cell lung cancer cells via the MAP4K1/JNK signaling pathway. EBioMedicine (2019) 42:311–25. doi: 10.1016/j.ebiom.2019.03.045 PMC6491656 30922965

[384] Ramos FS Serino LT Carvalho CM Lima RS Urban CA Cavalli IJ. PDIA3 and PDIA6 gene expression as an aggressiveness marker in primary ductal breast cancer. Genet Mol Res (2015) 14(2):6960–7. doi: 10.4238/2015.June.26.4 26125904

[385] Cheng HP Liu Q Li Y Li XD Zhu CY. The inhibitory effect of PDIA6 downregulation on bladder cancer cell proliferation and invasion. Oncol Res Featuring Preclinical Clin Cancer Ther (2017) 25(4):587–93. doi: 10.3727/096504016X14761811155298 PMC7841030 27760590

[386] Yan C Song X Wang S Wang J Li L. Knockdown of PDIA6 inhibits cell proliferation and enhances the chemosensitivity in gastric cancer cells. Cancer Manage Res (2020) 12:11051. doi: 10.2147/CMAR.S267711 PMC7646476 33173338

[387] Ma Y Xia P Wang Z Xu J Zhang L Jiang Y. PDIA6 promotes pancreatic cancer progression and immune escape through CSN5-mediated deubiquitination of β-catenin and PD-L1. Neoplasia (2021) 23(9):912–28. doi: 10.1016/j.neo.2021.07.004 PMC8329431 34325342

[388] Liu N Wu T Ma Y Cheng H Li W Chen M. Identification and validation of RB1 as an immune-related prognostic signature based on tumor mutation burdens in bladder cancer. Anti-Cancer Drugs (2023) 34(2):269–80. doi: 10.1097/CAD.0000000000001399 PMC9815815 36206128

[389] Dyson NJ. RB1: a prototype tumor suppressor and an enigma. Genes Dev (2016) 30(13):1492–502. doi: 10.1101/gad.282145 PMC4949322 27401552

[390] Moreno-Navarrete JM Petrov P Serrano M Ortega F Garcia-Ruiz E Oliver P. Decreased RB1 mRNA, protein, and activity reflect obesity-induced altered adipogenic capacity in human adipose tissue. Diabetes (2013) 62(6):1923–31. doi: 10.2337/db12-0977 PMC3661645 23315497

[391] Janostiak R Torres-Sanchez A Posas F de Nadal E. Understanding retinoblastoma post-translational regulation for the design of targeted cancer therapies. Cancers (Basel) (2022) 14(5):1265. doi: 10.3390/cancers14051265 35267571 PMC8909233

[392] Zuniga J Buendia-Roldan I Zhao Y Jimenez L Torres D Romo J. Genetic variants associated with severe pneumonia in A/H1N1 influenza infection. Eur Respir J (2012) 39(3):604–10. doi: 10.1183/09031936.00020611 PMC3816497 21737555

[393] Mehrbod P Eybpoosh S Farahmand B Fotouhi F Khanzadeh Alishahi M. Association of the host genetic factors, hypercholesterolemia and diabetes with mild influenza in an Iranian population. Virol J (2021) 18(1):1–11. doi: 10.1186/s12985-021-01486-3 33766078 PMC7993858

[394] Chen H Luo J Guo J. Identification of an alternative splicing signature as an independent factor in colon cancer. BMC Cancer (2020) 20(1):1–11. doi: 10.1186/s12885-020-07419-7 PMC7510085 32962686

[395] Sachamitr P Ho JC Ciamponi FE Ba-Alawi W Coutinho FJ Guilhamon P. PRMT5 inhibition disrupts splicing and stemness in glioblastoma. Nat Commun (2021) 12(1):1–17. doi: 10.1038/s41467-021-21204-5 33579912 PMC7881162

[396] Wu J Wang M Han L Zhang H Lei S Zhang Y. RNA modification-related variants in genomic loci associated with body mass index. Hum Genomics (2022) 16(1):25. doi: 10.1186/s40246-022-00403-1 35879730 PMC9316745

[397] Speliotes EK Willer CJ Berndt SI Monda KL Thorleifsson G Jackson AU. Association analyses of 249,796 individuals reveal 18 new loci associated with body mass index. Nat Genet (2010) 42(11):937–48. doi: 10.1038/ng.686 PMC3014648 20935630

[398] Augustus GJ Ellis NA. Colorectal cancer disparity in African Americans: risk factors and carcinogenic mechanisms. Am J Pathol (2018) 188(2):291–303. doi: 10.1016/j.ajpath.2017.07.023 29128568 PMC5785537

[399] O'Connor BP Eun SY Ye Z Zozulya AL Lich JD Moore CB. Semaphorin 6D regulates the late phase of CD4+ T cell primary immune responses. Proc Natl Acad Sci (2008) 105(35):13015–20. doi: 10.1073/pnas.0803386105 PMC2529027 18728195

[400] Kanth SM Gairhe S Torabi-Parizi P. The role of semaphorins and their receptors in innate immune responses and clinical diseases of acute inflammation. Front Immunol (2021) 12:672441. doi: 10.3389/fimmu.2021.672441 34012455 PMC8126651

[401] Kang S Nakanishi Y Kioi Y Okuzaki D Kimura T Takamatsu H. Semaphorin 6D reverse signaling controls macrophage lipid metabolism and anti-inflammatory polarization. Nat Immunol (2018) 19(6):561–70. doi: 10.1038/s41590-018-0108-0 29777213

[402] Baxter DE Allinson LM Al Amri WS Poulter JA Pramanik A Thorne JL. MiR-195 and Its Target SEMA6D regulate chemoresponse in breast cancer. Cancers (2021) 13(23):5979. doi: 10.3390/cancers13235979 34885090 PMC8656586

[403] Lu Q Zhu L. The role of semaphorins in metabolic disorders. Int J Mol Sci (2020) 21(16):5641. doi: 10.3390/ijms21165641 32781674 PMC7460634

[404] Stokowski RP Pant PV Dadd T Fereday A Hinds DA Jarman C. A genomewide association study of skin pigmentation in a south asian population. Am J Hum Genet (2007) 81(6):1119–32. doi: 10.1086/522235 PMC2276347 17999355

[405] Kasperaviciūte D Catarino CB Heinzen EL Depondt C Cavalleri GL Caboclo LO. Common genetic variation and susceptibility to partial epilepsies: a genome-wide association study. Brain (2010) 133(Pt 7):2136–47. doi: 10.1093/brain/awq130 PMC2892941 20522523

[406] Moffatt MF Gut IG Demenais F Strachan DP Bouzigon E Heath S. A large-scale, consortium-based genomewide association study of asthma. N Engl J Med (2010) 363(13):1211–21. doi: 10.1056/NEJMoa0906312 PMC4260321 20860503

[407] Chen D Li Y Wang L Jiao K. SEMA6D expression and patient survival in breast invasive carcinoma. Int J Breast Cancer (2015) 2015:539721. doi: 10.1155/2015/539721 25973277 PMC4417987

[408] Attar H Bedard K Migliavacca E Gagnebin M Dupre Y Descombes P. Extensive natural variation for cellular hydrogen peroxide release is genetically controlled. PloS One (2012) 7(8):e43566. doi: 10.1371/journal.pone.0043566 22952707 PMC3430705

[409] Mendoza-Alvarez A Guillen-Guio B Baez-Ortega A Hernandez-Perez C Lakhwani-Lakhwani S Maeso MDC. Whole-exome sequencing identifies somatic mutations associated with mortality in metastatic clear cell kidney carcinoma. Front Genet (2019) 10:439. doi: 10.3389/fgene.2019.00439 31156702 PMC6529576

[410] Pena-Chilet M Esteban-Medina M Falco MM Rian K Hidalgo MR Loucera C. Using mechanistic models for the clinical interpretation of complex genomic variation. Sci Rep (2019) 9(1):18937. doi: 10.1038/s41598-019-55454-7 31831811 PMC6908734

[411] Wang Y Li J Li J Li P Wang L Di L. An enhancer-based analysis revealed a new function of androgen receptor in tumor cell immune evasion. Front Genet (2020) 11:595550. doi: 10.3389/fgene.2020.595550 33343635 PMC7738566

[412] Pettinelli P Arendt BM Schwenger KJP Sivaraj S Bhat M Comelli EM. Relationship between hepatic gene expression, intestinal microbiota, and inferred functional metagenomic analysis in NAFLD. Clin Transl Gastroenterol (2022) 13(7):e00466. doi: 10.14309/ctg.0000000000000466 35166723 PMC10476782

[413] Canhong W Jiarui LI Jiahui C Haizhu T. SIPA1L2 as a risk factor implicated in Alzheimer's disease. J Univ Sci Technol China (2021) 51(2):147. doi: 10.52396/JUST-2021-0008

[414] Wang Y Chang Q Li Y. Racial differences in urinary bladder cancer in the United States. Sci Rep (2018) 8(12521):12521. doi: 10.1038/s41598-018-29987-2 30131523 PMC6104025

[415] Ghosh P Campos VJ Vo DT Guccione C Goheen-Holland V Tindle C. AI-assisted discovery of an ethnicity-influenced driver of cell transformation in esophageal and gastroesophageal junction adenocarcinomas. JCI Insight (2022) 7(18):e161334. doi: 10.1172/jci.insight.161334 36134663 PMC9675486

[416] Muhimpundu S Conway RBN Warren Andersen S Lipworth L Steinwandel MD Blot WJ. Racial differences in hepatocellular carcinoma incidence and risk factors among a low socioeconomic population. Cancers (2021) 13(15):3710. doi: 10.3390/cancers13153710 34359611 PMC8345125

[417] Capmany A Gambarte Tudela J Alonso Bivou M Damiani MT. Akt/AS160 signaling pathway inhibition impairs infection by decreasing Rab14-controlled sphingolipids delivery to chlamydial inclusions. Front Microbiol (2019) 10:666. doi: 10.3389/fmicb.2019.00666 31001235 PMC6456686

[418] Jiang XH Sun JW Xu M Jiang XF Liu CF Lu Y. Frequent hyperphosphorylation of AS160 in breast cancer. Cancer Biol Ther (2010) 10(4):362–7. doi: 10.4161/cbt.10.4.12426 20574165

[419] Cheng JC McBrayer SK Coarfa C Dalva-Aydemir S Gunaratne PH Carpten JD. Expression and phosphorylation of the AS160_v2 splice variant supports GLUT4 activation and the Warburg effect in multiple myeloma. Cancer Metab (2013) 1(1):1–8. doi: 10.1186/2049-3002-1-14 24280290 PMC4178207

[420] Moltke I Grarup N Jørgensen ME Bjerregaard P Treebak JT Fumagalli M. A common Greenlandic TBC1D4 variant confers muscle insulin resistance and type 2 diabetes. Nature (2014) 512(7513):190–3. doi: 10.1038/nature13425 25043022

[421] Chen MH Raffield LM Mousas A Sakaue S Huffman JE Moscati A. Trans-ethnic and ancestry-specific blood-cell genetics in 746,667 individuals from 5 global populations. Cell (2020) 182(5):1198–213. e14. doi: 10.1016/j.cell.2020.06.045 32888493 PMC7480402

[422] Sakaue S Kanai M Tanigawa Y Karjalainen J Kurki M Koshiba S. A cross-population atlas of genetic associations for 220 human phenotypes. Nat Genet (2021) 53(10):1415–24. doi: 10.1038/s41588-021-00931-x PMC12208603 34594039

[423] Vuckovic D Bao EL Akbari P Lareau CA Mousas A Jiang T. The polygenic and monogenic basis of blood traits and diseases. Cell (2020) 182(5):1214–31.e11. doi: 10.1016/j.cell.2020.08.008 32888494 PMC7482360

[424] Kanapuru B Fernandes LL Fashoyin-Aje LA Baines AC Bhatnagar V Ershler R. Analysis of racial and ethnic disparities in multiple myeloma US FDA drug approval trials. Blood Adv (2022) 6(6):1684–91. doi: 10.1182/bloodadvances.2021005482 PMC8941450 35114691

[425] Lyu J Wang P Xu T Shen Y Cui Z Zheng M. Thymic-specific regulation of TCR signaling by Tespa1. Cell Mol Immunol (2019) 16(12):897–907. doi: 10.1038/s41423-019-0259-4 31316154 PMC6884599

[426] Liu L Liu Y. Pan-cancer analysis of the prognostic and immunological role of thymocyte-expressed positive selection-associated protein 1 (TESPA1) in human tumors. Research Square pre-print (2022). doi: 10.21203/rs.3.rs-1249485/v1

[427] Luan Y Luan Y Yuan RX Feng Q Chen X Yang Y. Structure and function of mitochondria-associated endoplasmic reticulum membranes (MAMs) and their role in cardiovascular diseases. Oxid Med Cell Longevity (2021) 2021:4578809. doi: 10.1155/2021/4578809 PMC8289621 34336092

[428] Anderson CA Boucher G Lees CW Franke A D'Amato M Taylor KD. Meta-analysis identifies 29 additional ulcerative colitis risk loci, increasing the number of confirmed associations to 47. Nat Genet (2011) 43(3):246–52. doi: 10.1038/ng.764 PMC3084597 21297633

[429] Mead S Uphill J Beck J Poulter M Campbell T Lowe J. Genome-wide association study in multiple human prion diseases suggests genetic risk factors additional to PRNP. Hum Mol Genet (2012) 21(8):1897–906. doi: 10.1093/hmg/ddr607 PMC3313791 22210626

[430] Kirtane K Lee SJ. Racial and ethnic disparities in hematologic Malignancies. Blood (2017) 130(15):1699–705. doi: 10.1182/blood-2017-04-778225 PMC5639484 28724539

[431] Fujimoto A Totoki Y Abe T Boroevich KA Hosoda F Nguyen HH. Whole-genome sequencing of liver cancers identifies etiological influences on mutation patterns and recurrent mutations in chromatin regulators. Nat Genet (2012) 44(7):760–4. doi: 10.1038/ng.2291 22634756

[432] Holm M Joenväärä S Saraswat M Mustonen H Tohmola T Ristimäki A. Identification of several plasma proteins whose levels in colorectal cancer patients differ depending on outcome. FASEB BioAdvances (2019) 1(12):723–30. doi: 10.1096/fba.2019-00062 PMC6996405 32123817

[433] Liu H Bai N Liu S Liu W Zhou Q Zhang H. Exploring feature genes for ischemic stroke based on bioinformatics and machine learning. Atherosclerosis (2022) 355:238. doi: 10.1016/j.atherosclerosis.2022.06.920

[434] Yucesan E Hatirnaz Ng O Yalniz FF Yilmaz H Salihoglu A Sudutan T. Copy-number variations in adult patients with chronic immune thrombocytopenia. Expert Rev Hematol (2020) 13(11):1277–87. doi: 10.1080/17474086.2020.1819786 32885695

[435] Liu G Tang H Li C Zhen H Zhang Z Sha Y. Prognostic gene biomarker identification in liver cancer by data mining. Am J Trans Res (2021) 13(5):4603. PMC8205730 34150040

[436] Seyres D Cabassi A Lambourne JJ Burden F Farrow S McKinney H. Transcriptional, epigenetic and metabolic signatures in cardiometabolic syndrome defined by extreme phenotypes. Clin Epigenet (2022) 14(1):1–24. doi: 10.1186/s13148-022-01257-z PMC8917653 35279219

[437] Masuda K Kornberg A Miller J Lin S Suek N Botella T. Multiplexed single-cell analysis reveals prognostic and nonprognostic T cell types in human colorectal cancer. JCI Insight (2022) 7(7):e154646. doi: 10.1172/jci.insight.154646 35192548 PMC9057629

[438] Liang WS Craig DW Carpten J Borad MJ Demeure MJ Weiss GJ. Genome-wide characterization of pancreatic adenocarcinoma patients using next generation sequencing. PloS One (2012) 7(10):e43192. doi: 10.1371/journal.pone.0043192 23071490 PMC3468610

[439] Lazare C Zhi W Dai J Cao C Sookha RR Wang L. A pilot study comparing the genetic molecular biology of gestational and non-gestational choriocarcinoma. Am J Transl Res (2019) 11(11):7049–62. PMC6895522 31814908

[440] Huang C Jia Y Yang S Chen B Sun H Shen F. Characterization of ZNF23, a KRAB-containing protein that is downregulated in human cancers and inhibits cell cycle progression. Exp Cell Res (2007) 313(2):254–63. doi: 10.1016/j.yexcr.2006.10.009 17137575

[441] Ruocco MR Avagliano A Granato G Vigliar E Masone S Montagnani S. Metabolic flexibility in melanoma: A potential therapeutic target. Semin Cancer Biol (2019) 59:187–207. doi: 10.1016/j.semcancer.2019.07.016 31362075

[442] Urrutia R. KRAB-containing zinc-finger repressor proteins. Genome Biol (2003) 4(10):1–8. doi: 10.1186/gb-2003-4-10-231 PMC328446 14519192

[443] Patel S Howard D Chowdhury N Derieux C Wellslager B Yilmaz O. Characterization of human genes modulated by porphyromonas gingivalis highlights the ribosome, hypothalamus, and cholinergic neurons. Front Immunol (2021) 12:646259. doi: 10.3389/fimmu.2021.646259 34194426 PMC8236716

[444] Schnabl B Valletta D Kirovski G Hellerbrand C. Zinc finger protein 267 is up-regulated in hepatocellular carcinoma and promotes tumor cell proliferation and migration. Exp Mol Pathol (2011) 91(3):695–701. doi: 10.1016/j.yexmp.2011.07.006 21840307 PMC3342774

[445] Long X Liu X Deng T Chen J Lan J Zhang S. LARP6 suppresses colorectal cancer progression through ZNF267/SGMS2-mediated imbalance of sphingomyelin synthesis. J Exp Clin Cancer Res (2023) 42(1):33. doi: 10.1186/s13046-023-02605-4 36691044 PMC9872320

[446] Yang H Wang L Zheng Y Hu G Ma H Shen L. Knockdown of zinc finger protein 267 suppresses diffuse large B-cell lymphoma progression, metastasis, and cancer stem cell properties. Bioengineered (2022) 13(1):1686–701. doi: 10.1080/21655979.2021.2014644 PMC8805851 35001816

[447] Schnabl B Hu K Mühlbauer M Hellerbrand C Stefanovic B Brenner DA. Zinc finger protein 267 is up-regulated during the activation process of human hepatic stellate cells and functions as a negative transcriptional regulator of MMP-10. Biochem Biophys Res Commun (2005) 335(1):87–96. doi: 10.1016/j.bbrc.2005.07.043 16054593

[448] Schnabl B Czech B Valletta D Weiss TS Kirovski G Hellerbrand C. Increased expression of Zinc finger protein 267 in non-alcoholic fatty liver disease. Int J Clin Exp Pathol (2011) 4(7):661. 22076166 PMC3209606

[449] Primm KM Malabay AJ Curry T Chang S. Who, where, when: Colorectal cancer disparities by race and ethnicity, subsite, and stage. Cancer Med (2023) 12(13):14767–80. doi: 10.1002/cam4.6105 PMC10358189 37212502

[450] Gustafson EA Seymour KA Sigrist K Rooij DGDE Freiman RN. ZFP628 is a TAF4b-interacting transcription factor required for mouse spermiogenesis. Mol Cell Biol (2020) 40(7):e00228-19. doi: 10.1128/MCB 31932482 PMC7076252

[451] Aspedon A Groisman EA. The antibacterial action of protamine: evidence for disruption of cytoplasmic membrane energization in Salmonella typhimurium. Microbiology (1996) 142(12):3389–97. doi: 10.1099/13500872-142-12-3389 9004502

[452] Pink DA Hasan FM Quinn BE Winterhalter M Mohan M Gill TA. Interaction of protamine with gram-negative bacteria membranes: possible alternative mechanisms of internalization in Escherichia coli, Salmonella typhimurium and Pseudomonas aeruginosa. J Pept Sci (2014) 20(4):240–50. doi: 10.1002/psc.2610 24453038

[453] Meklat F Zhang Y Shahriar M Ahmed SU Li W Voukkalis N. Identification of protamine 1 as a novel cancer-testis antigen in early chronic lymphocytic leukaemia. Br J Haematol (2009) 144(5):660–6. doi: 10.1111/j.1365-2141.2008.07502.x PMC2752686 19036087

[454] Ren S Chen X Tian X Yang D Dong Y Chen F. The expression, function, and utilization of Protamine1: a literature review. Transl Cancer Res (2021) 10(11):4947–57. doi: 10.21037/tcr-21-1582 PMC8799248 35116345

[455] Ramzan R Michels S Weber P Rhiel A Irqsusi M Rastan AJ. Protamine sulfate induces mitochondrial hyperpolarization and a subsequent increase in reactive oxygen species production. J Pharmacol Exp Ther (2019) 370(2):308–17. doi: 10.1124/jpet.119.257725 31160469

[456] Chen GY Muramatsu H Ichihara-Tanaka K Muramatsu T. ZEC, a zinc finger protein with novel binding specificity and transcription regulatory activity. Gene (2004) 340(1):71–81. doi: 10.1016/j.gene.2004.06.016 15556296

[457] Beck T Rowlands T Shorter T Brookes AJ. GWAS central: an expanding resource for finding and visualising genotype and phenotype data from genome-wide association studies. Nucleic Acids Res (2023) 51(D1):D986–D93. doi: 10.1093/nar/gkac1017 PMC9825503 36350644

[458] Hirata Y Yanaihara N Yanagida S Fukui K Iwadate K Kiyokawa T. Molecular genetic analysis of nongestational choriocarcinoma in a postmenopausal woman: a case report and literature review. Int J Gynecological Pathol (2012) 31(4):364–8. doi: 10.1097/PGP.0b013e318241d556 22653351

[459] Higashi T Tokuda S Kitajiri S Masuda S Nakamura H Oda Y. Analysis of the A’ngulin’ proteins LSR, ILDR1 and ILDR2–tricellulin recruitment, epithelial barrier function and implication in deafness pathogenesis. J Cell Sci (2013) 126(Pt 4):966–77. doi: 10.1242/jcs.116442 23239027

[460] Chandra R Wang Y Shahid RA Vigna SR Freedman NJ Liddle RA. Immunoglobulin-like domain containing receptor 1 mediates fat-stimulated cholecystokinin secretion. J Clin Invest (2013) 123(8):3343–52. doi: 10.1172/jci68587 PMC3726170 23863714

[461] Gong Y Himmerkus N Sunq A Milatz S Merkel C Bleich M. ILDR1 is important for paracellular water transport and urine concentration mechanism. Proc Natl Acad Sci U.S.A. (2017) 114(20):5271–6. doi: 10.1073/pnas.1701006114 PMC5441773 28461473

[462] Trapnell BC Nakata K Bonella F Campo I Griese M Hamilton J. Pulmonary alveolar proteinosis. Nat Rev Dis Primers (2019) 5(1):16. doi: 10.1038/s41572-019-0066-3 30846703

[463] Oughtred R Rust J Chang C Breitkreutz BJ Stark C Willems A. The BioGRID database: A comprehensive biomedical resource of curated protein, genetic, and chemical interactions. Protein Sci (2021) 30(1):187–200. doi: 10.1002/pro.3978 33070389 PMC7737760

[464] Del Toro N Shrivastava A Ragueneau E Meldal B Combe C Barrera E. The IntAct database: efficient access to fine-grained molecular interaction data. Nucleic Acids Res (2022) 50(D1):D648–53. doi: 10.1093/nar/gkab1006 PMC8728211 34761267

